# Nonclassical Mechanism in the Cyclodehydration of Diols Catalyzed by a Bifunctional Iridium Complex

**DOI:** 10.1002/chem.201805460

**Published:** 2019-01-23

**Authors:** Greco González Miera, Aitor Bermejo López, Elisa Martínez‐Castro, Per‐Ola Norrby, Belén Martín‐Matute

**Affiliations:** ^1^ Department of Organic Chemistry Stockholm University Stockholm 10691 Sweden; ^2^ Early Product Development, Pharmaceutical Sciences, IMED Biotech Unit AstraZeneca Gothenburg Sweden

**Keywords:** Hammett–Brown, hydride, hydrogen transfer, iridium, kinetic isotope effect

## Abstract

1,4‐ and 1,5‐diols undergo cyclodehydration upon treatment with cationic N‐heterocyclic carbene (NHC)–Ir^III^ complexes to give tetrahydrofurans and tetrahydropyrans, respectively. The mechanism was investigated, and a metal‐hydride‐driven pathway was proposed for all substrates, except for very electron‐rich ones. This contrasts with the well‐established classical pathways that involve nucleophilic substitution.

## Graphical abstract

**Hydrogen transfer vs. acid catalysis**: Mechanistic studies on the iridium‐catalyzed cyclodehydration of a series of diols are carried out. Hammett and Hammett–Brown analyses of the reactivity data are compared, along with kinetic isotope effect and crossover experiments. This analysis enabled the elucidation of the reaction mechanisms, which are found to be dependent on the electronic properties of the substrates (see picture).

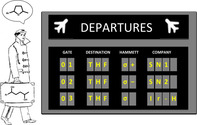

## Introduction

NHC–Ir complexes (NHC=N‐heterocyclic carbene) have proven to be excellent catalysts in numerous processes, particularly in dehydrogenations and transfer hydrogenations.^1^, ^2^, ^3^,  5a,5c,5d,5e NHCs can be relatively easily functionalized to provide the desired reactivity. Their versatility has recently been highlighted by Peris in a recent review article,^4^ in which the author refers to NHCs as “smart ligands”.

**Scheme 1:**
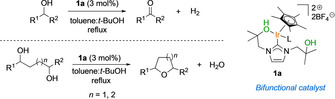
Acceptorless dehydrogenation of alcohols (top) and redox cyclization of diols (bottom) catalyzed by complex **1** **a**.

We have previously investigated the activity of Ir^III^ complexes that bear functionalized NHC ligands (**1**) in C−N bond‐forming reactions with anilines and alcohols. Mechanistic investigations indicated that the oxygen functionality on the NHC ligand was involved in proton transfer steps, which enables reactions to be performed under base‐free conditions.3b The binfunctional nature of the NHC–Ir complexes (**1**) was also explored in the acceptorless dehydrogenation of alcohols^2^ (Scheme 1, top). Here, we observed that, when two 1,4‐diols, 1‐phenyl‐1,4‐pentanediol (**2** **a**) and 1,4‐diphenyl‐1,4‐butanediol (**2** **j**), were reacted with catalyst **1** **a**, tetrahydrofuran products were formed in very good yields (Scheme 1, bottom) instead of the expected products derived from a dehydrogenation process (Scheme 1, top). The synthesis of this type of cyclic ether from diols is a well‐established procedure that can be mediated by Brønsted^5^ or Lewis acids,^6^ and mechanisms that involve nucleophilic substitution have been proposed.^7^ Cyclizations under basic conditions have also been reported.^8^ However, when transition‐metal complexes were used, the possibility that an alternative hydrogen‐borrowing (or hydrogen‐autotransfer) mechanism could be operating was not investigated; this motivated us to study the mechanism of these formal cyclodehydration reactions.^9^ We found that the mechanism for the dehydrogenation of benzylic alcohols by catalyst **1** **a** involved an initial hydrogen‐transfer step with concomitant formation of an iridium–hydride species.^2^ The hydroxy/alkoxide functionality on the carbene ligand participated in proton‐transfer steps. We were intrigued by the possibility that a similar hydrogen‐transfer mechanism could also be operating in the case of the diols, and we have now studied the cyclodehydration reactions of 1,4‐ and 1,5‐diols catalyzed by NHC–iridium complexes **1** **a**–**c**. In this paper, we propose mechanistic pathways that are dependent on the electronic properties of the diols as well as on whether the substrate is a 1,4‐ or a 1,5‐diol.

## Results

We tested a series of NHC–Ir^III^ complexes in the cyclodehydration reaction of 1‐phenyl‐1,4‐pentanediol (**2** **a**; Table 1).^2^ The optimized reaction conditions for the acceptorless alcohol dehydrogenation (AAD) reaction (Scheme 1, top) had previously been tested on diol **2** **a** (i.e., iridium complex **1** **a** in a mixture of toluene and *t*‐butanol (2.6:1, v/v) heated at reflux), and under these conditions, tetrahydrofuran **3** **a** was formed in excellent yield (91 %, Table 1, entry 1).^2^ In contrast, neutral iridium dichloride complex **1** **b** did not promote the cyclization; instead, mono‐ and dioxidized linear compounds **4** **a** and **5** **a**, as well as deoxygenated ketone **6** **a** (see the Supporting Information)^10^ were detected in the crude mixture at 80 % conversion of substrate **2** **a** (entry 2). Biscationic bifunctional catalyst **1 c**, which has an NHC ligand with only one hydroxy‐functionalized wingtip, gave the tetrahydrofuran product (**3** **a**) in a low yield of 31 % and a mixture of oxidized linear compounds (entry 3). The commercially available complex [Cp*IrCl_2_]_2_ (**1** **d**) was also tested, and this gave product **3** **a** in only 11 % yield (entry 4) along with higher yields of oxidized linear byproducts. In a control experiment carried out in the absence of any iridium complex under otherwise identical reaction conditions, diol **2** **a** did not undergo any reaction (entry 5). When toluene was used as the sole solvent, the catalytic activity of complex **1** **a** towards the formation of tetrahydrofuran **3** **a** decreased; this product was formed in a lower yield of 70 % (entry 6 vs. 1).

**Table 1:** Cyclodehydration of diols catalyzed by Ir^III^ complexes.^[a]^

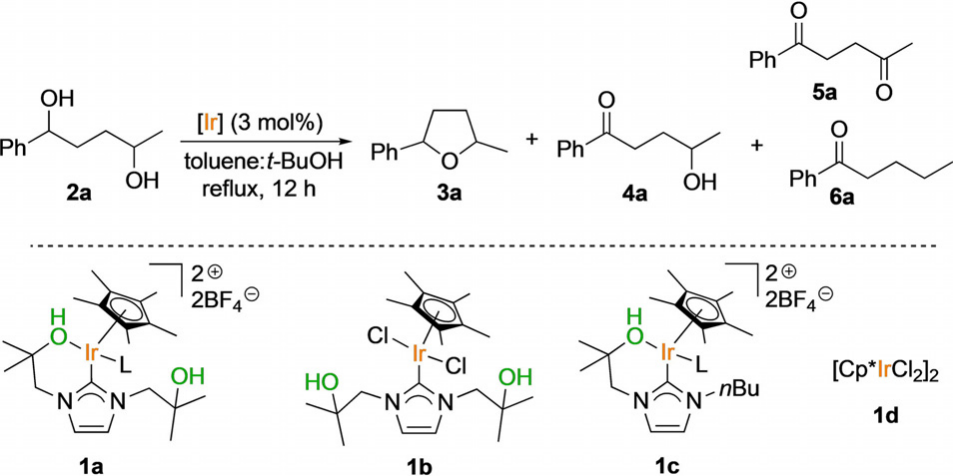						
Entry	[Ir]	**2** **a** [%]	**3** **a** [%]	**4** **a** [%]	**5** **a** [%]	**6** **a** [%]
1	**1** **a**	n.d.^[b]^	91	5	4	n.d.^[b]^
2	**1** **b**	20	n.d.^[b]^	45	13	23
3	**1** **c**	8	31	30	13	18
4	**1** **d**	10	17	26	12	35
5	–	>95	n.d.^[b]^	n.d.^[b]^	n.d.^[b]^	n.d.^[b]^
6^[c]^	**1** **a**	14	70	3	14	n.d.^[b]^

[a] Reaction conditions: diol (1 mmol), [Ir] (0.03 mmol, 3 mol %), toluene (2.6 mL), *t*BuOH (1 mL), 80 °C or heated at reflux, 24 h. Yield determined by ^1^H NMR spectroscopy. [b] n.d.=not detected. [c] In toluene as the sole solvent.

Iridium complex **1** **a** was then used as the catalyst in the cyclodehydration of a series of 1,4‐diols (**2** **a**–**l**) and 1,5‐diols (**2** **m**–**n**) by using the conditions of Table 1, entry 1 (Table 2). For 1,4‐diols that contained only *sec*‐alcohols, the corresponding tetrahydrofuran products **3** **a**–**k** were formed in good to excellent yields. The ^1^H NMR spectra of the products indicated the presence of diastereoisomeric mixtures (see the Supporting Information). The reaction even worked well for aliphatic biomass‐derived 2,5‐hexanediol (**2** **k**), which gave 2,5‐dimethyltetrahydrofuran (**3** **k**), an important industrial additive.^11^

**Table 2:** Cyclodehydration of diols catalyzed by complex **1** **a**.^[a]^

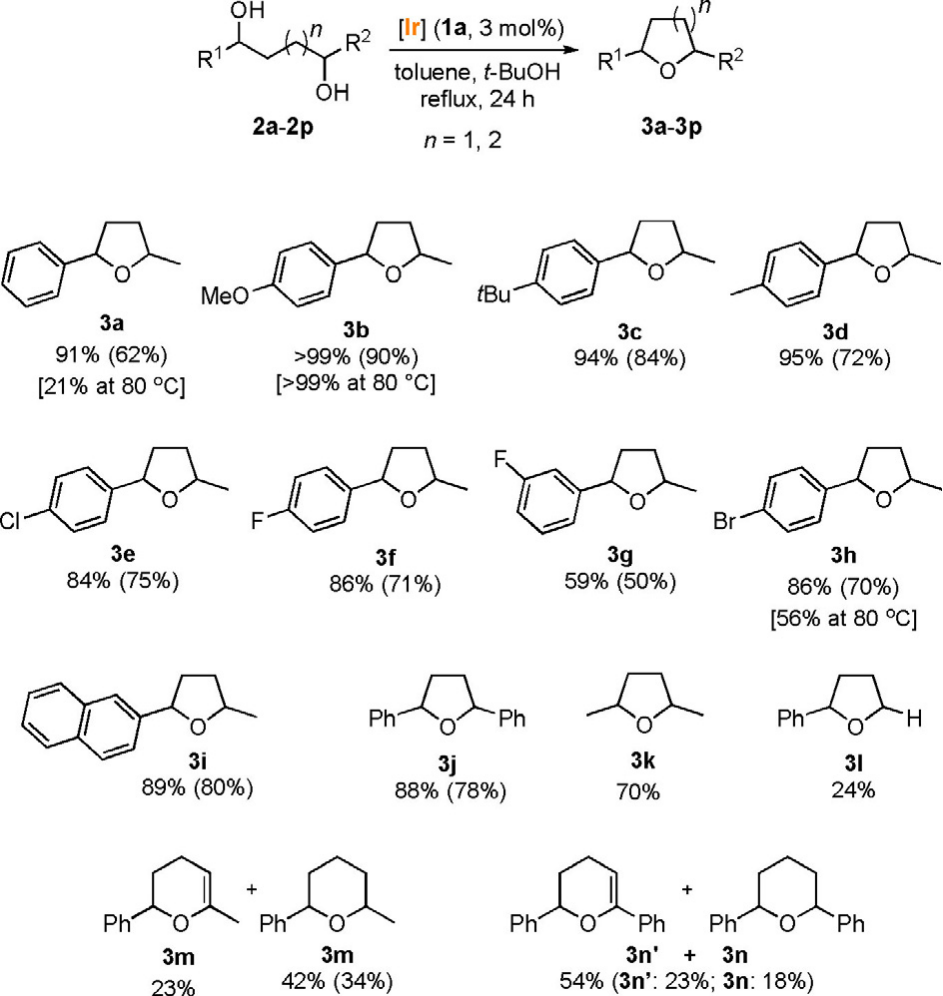

[a] Reaction conditions: diol (1 mmol), **1** **a** (0.03 mmol, 3 mol %), toluene (2.6 mL), *t*BuOH (1 mL), 80 °C or heated at reflux, 24 h. Yield determined by ^1^H NMR spectroscopy. Yields of isolated products in parentheses.

When 1,4‐diol **2** **l**, which contains a *sec‐* and a primary alcohol, was subjected to the reaction conditions, the yield of the product **3 l** dropped dramatically to only 24 %. This is consistent with our observations on the AAD reactions of primary alcohols catalyzed by complex **1** **a**.^2^ Unsaturated diols yielded not cyclic ether derivatives but mixtures of diketones and deoxygenated ketones (see the Supporting Information). Importantly, when the reaction was tested under milder reaction conditions (80 °C), good yields were only obtained for the very electron‐rich diol **2** **b** (to give **3** **b**; **3** **a** and **h** were formed in lower yields). Interestingly, 1,5‐diol substrates (**2** **m** and **n**) reached full conversion to give mixtures of products; the major products were six‐membered‐ring compounds: saturated cyclic ethers (**3** **m** and **n**) and 2,3‐dihydropyrans (**3** **m′** and **n′**). The presence of the unsaturated products suggests a net loss of dihydrogen for this family of substrates. Dihydropyran **3** **m′** was transformed into the corresponding tetrahydropyran **3** **m** in a subsequent hydrogenation step (see the Supporting Information).

**Scheme 2:**
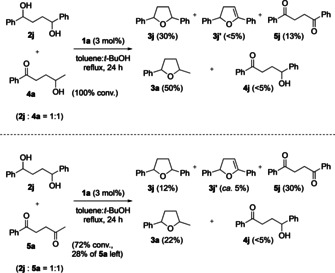
Crossover experiments for the cyclization of 1,4‐diol **2** **j** in the presence of ketoalcohol **4** **a** (top) or diketone **5** **a** (bottom).

Crossover experiments were carried out to gain some insight into the overall redox‐neutral reaction of diols. When a 1:1 mixture of diol **2** **j** and ketoalcohol **4** **a** was subjected to the reaction conditions, cyclic structures **3** **j** and **a** were obtained (Scheme 2, top). The reaction mixture also contained oxidized intermediates 2,3‐dihydrofuran **3** **j′**, ketoalcohol **4** **j**, and diketone **5** **j**. Similarly, a 1:1 mixture of diol **2** **j** and diketone **5** **a** was subjected to the reaction conditions (Scheme 2, bottom), and after 24 h, tetrahydrofurans **3** **j** and **a** were obtained, along with the corresponding oxidized intermediates **3** **j′**, **4** **j**, and **5** **j**.

**Figure 1:**
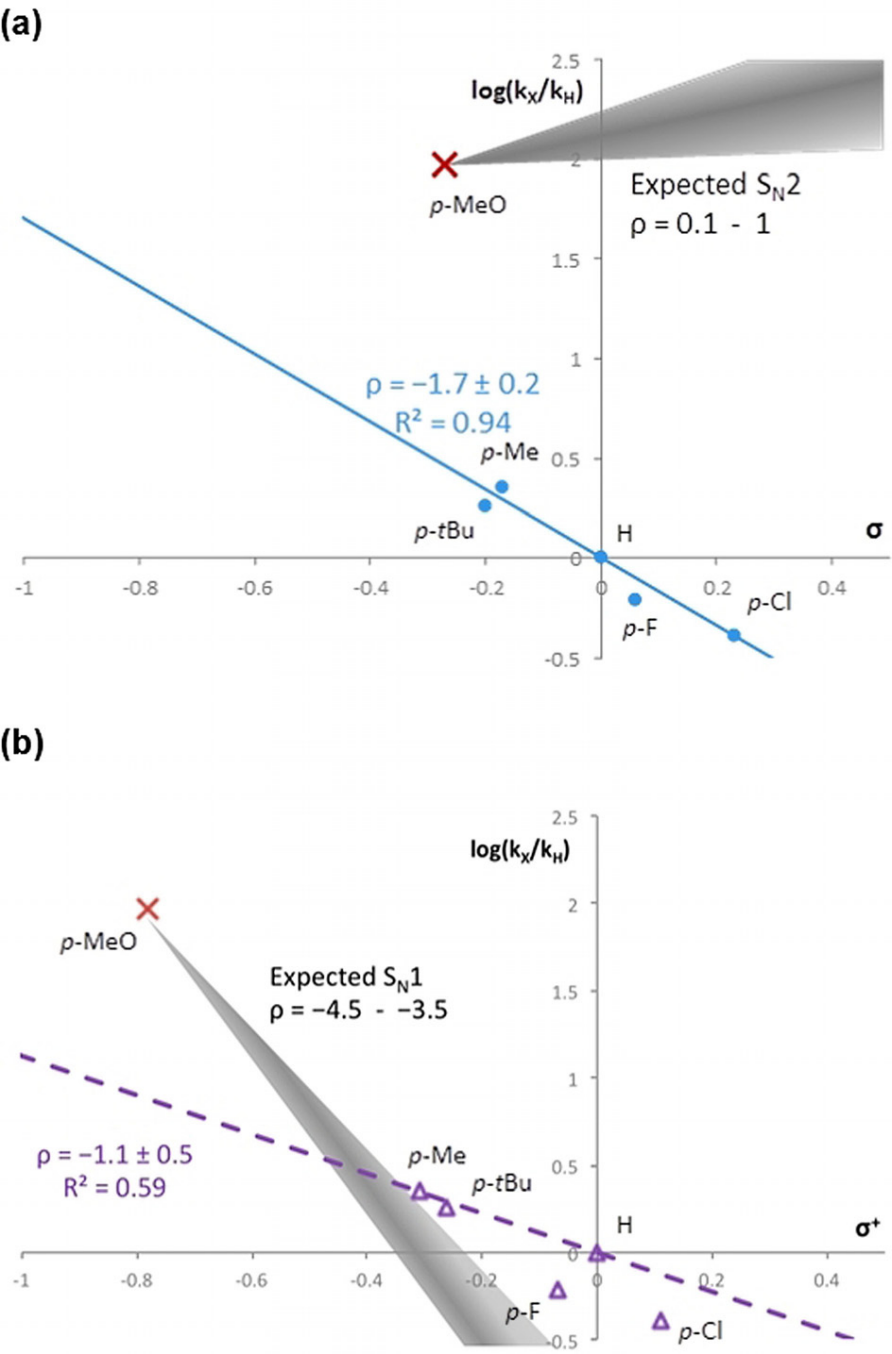
Hammett plots for the cyclodehydration of diols **2** **a**–**f**: a) log(*k*_X_/*k*_H_) versus *σ*, log(*k*_X_/*k*_H_)=(−1.7±0.2)*σ*, *R* ^2^=0.94; b) log(*k*_X_/*k*_H_) versus *σ* ^+^_,_ log(*k*_X_/*k*_H_)=(−1.1±0.5)*σ* ^+^, *R* ^2^=0.59. The shaded regions show the expected areas for log(*k*_X_/*k*_H_) if the substrates were to follow a) an S_N_2 or b) an S_N_1 mechanism. Each point corresponds to an average of three experiments. Note: **2** **b** (red cross) is not used for the correlations (see the Discussion).

Hammett studies on the cyclization of five different *para*‐functionalized 1‐aryl‐1,4‐pentanediol substrates **2 a–f** are shown in Figure 1 (see the Supporting Information).^12^ The conversions were monitored by in situ ^1^H NMR spectroscopy. For electron‐poor 1,4‐diols and for 1,4‐diols with moderately electron‐rich substituents, plots of [log(*k*_X_/*k*_H_)] versus *σ* (Figure 1a) show a linear relationship with a negative slope of −1.73±0.22. The electron‐rich *para*‐methoxy‐substituted diol **2 b** deviates from this Hammett correlation, as it reacted about 10^4^ times faster than extrapolated (Figure 1a).^13^

Figure 1b also shows a plot of [log(*k*_X_/*k*_H_)] versus the Hammett–Brown *σ* ^+^ constants instead of the *σ* constants (see the Supporting Information).^14^

Kinetic isotope effect (KIE) studies were then carried out.^15^ The cyclodehydration rate of diol **2** **a** was compared to that of [D_2_]**2** **a**, and a KIE of 2.94±0.14 was observed (see the Supporting Information). This value suggests that the cleavage of the C−H(D) bond at the benzylic position occurs in the rate‐determining step. In contrast, a negligible KIE of 1.14±0.08 was obtained for the *p*‐methoxy‐substituted diols **2** **b** and [D_2_]**2** **b** (see the Supporting Information).

## Discussion

**Scheme 3:**
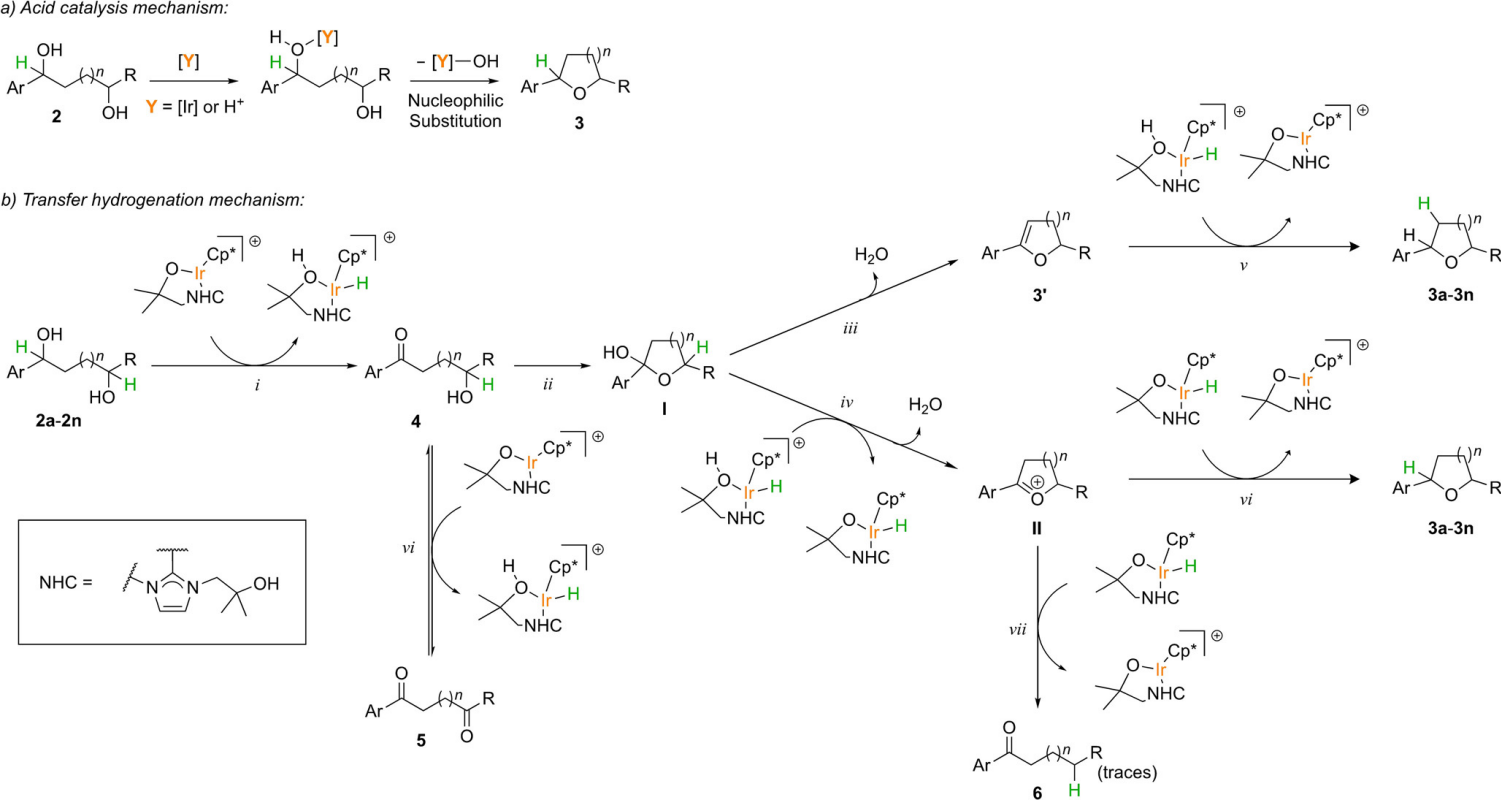
Proposed mechanism for the formation of cyclic ethers **3** (*n=*1,2).

Two possible mechanistic pathways are shown in Scheme 3. Scheme 3a shows a mechanism that proceeds through acid catalysis,^16^ which involves nucleophilic substitution (S_N_1 or S_N_2). Scheme 3b shows a redox‐neutral mechanism with carbonyl compounds and iridium hydrides as key intermediates. The functionalized NHC ligand of complex **1** **a** participates in proton‐shuffling steps.^3^ The iridium complex acts, in the first instance, as an acid catalyst, and in the second, as a hydrogen‐transfer catalyst. When we investigated the scope of this reaction (see above, Table 2), we found that diol **2** **b**, which has an electron‐rich *p*‐MeOC_6_H_4_ substituent, gave the tetrahydrofuran product **3** **b** in excellent yield, even when a lower temperature of 80 °C was used. Neither diols **2** **a** nor **b** gave any product when the reaction was carried out in the absence of an iridium catalyst (see above, Table 1, entry 5), under otherwise identical reaction conditions.

The Hammett plots (Figure 1a,b) clearly show that the *p*‐MeO‐substituted substrate **2** **b** reacts at a rate that is orders of magnitude higher than what would be predicted based on the log(*k*_X_/*k*_H_) of the other substrates. Owing to the excellent fitting of all substrates, excluding **2** **b**, in the Hammett plot (Figure 1a, substituent constants *σ*, *R* ^2^=0.94) compared with the Hammett–Brown plot (Figure 1b, substituent constants *σ*+, *R* ^2^=0.59), the S_N_1 pathway (i.e., through a fully developed positive charge in direct conjugation with the *para* substituent) can already be ruled out for these substrates. Closer analysis of Figure 1b gives further support to the absence of an S_N_1 pathway for diols **2** **a**,**c**–**f**. In general, for an S_N_1 mechanism, we would expect a linear fit with the *σ* ^*+*^ values, and a *ρ* value of around −4.^14^ In Figure 1b, the shaded area shows the range of gradients for typical *ρ* values in S_N_1 reactions, which range from −3.5 to −4.5 (by using the data point of diol **2** **b** as a reference point). If diols **2** **a**,**c**–**f** followed an S_N_1 pathway, their data points would fall within this shaded region (Figure 1b), and this is in clear disagreement with the experimental data. All substrates except **2** **b** lie above the expected S_N_1 plot bracket that is based on diol **2** **b** (Figure 1b, shaded region). In short, we can conclude that all the substrates except *p*‐MeO diol **2** **b** follow a faster neutral pathway instead of the alternative S_N_1 mechanism.

Therefore, we now have to consider which of the alternative neutral mechanisms, the S_N_2 or redox pathways (Scheme 3), is operating for diols **2** **a**,**c**–**f**. If the reaction followed an S_N_2 mechanism, we would expect to see a correlation with *σ* and a small positive *ρ* value (typical *ρ* values for S_N_2 reactions range from 0.1 to 1; Figure 1a, shaded area, determined by using the data point of diol **2** **b** as a reference point).^14^ Thus, diols **2** **a**,**c**–**f** would all be expected to have reactivities equal to or higher than that of *p*‐MeO diol **2** **b** (i.e., a positive *ρ* value for substrates with electron‐withdrawing substituents that have higher rates). This is, once again, in clear disagreement with the observed results. In fact, excluding cyclic ether **3** **b**, which is obviously formed by a different mechanism (c.f., KIE), the opposite reactivity trend was observed, as the data fit well to standard Hammett *σ* values (Figure 1a) with a negative *ρ* value of −1.7. This is very similar to what we reported before for a rate‐limiting Ir‐catalyzed hydrogen transfer from benzylic alcohols.3b

Therefore, we may conclude that there are two competing mechanisms. Normally, this situation results in a Hammett plot with two linear regions that show an upwards break, a so‐called “V” shape.^17^ In the peculiar case described here, this should instead be represented with two different Hammett plots, as the S_N_1 pathway correlates with *σ* ^*+*^ values, and the neutral‐redox pathway correlates with the neutral substituent constants *σ*. The inflection point can be estimated by looking into Figure 1b at a *σ* ^*+*^ value of around −0.3 to −0.4 at the intersection between the shaded region, which represents an S_N_1 mechanism from diol **2** **b** and the experimental Hammett–Brown plot (purple dashed line constructed from **2** **a**,**c**–**f**).

The substantial difference obtained in the KIE studies on diols **2** **a** versus **b** (2.94±0.14 vs. 1.14±0.08, respectively) also supports the operation of two distinct mechanistic pathways, which depend on the electronic properties of the substrates. Thus, in the case of diol **2** **a**, the C−H bond is broken in the rate‐determining step, in contrast to diol **2** **b**.

Further support for the redox pathway (Scheme 3b) for substrate **2** **a** was obtained in the crossover experiments (Scheme 2), as hydrogen was transferred between the diol substrates and the diketone or ketoalcohol additives. Furthermore, the cyclodehydration of 1,5‐diols **2** **m**–**n** gave mixtures of 2,3‐dihydropyrans **3′** and tetrahydropyrans **3**. The former products **3′** could only be formed through a mechanism that involves hydrogen transfer.^18^

In an attempt to obtain further evidence for the formation of carbocationic species in the cyclodehydration of diol **2** **b**, we carried out a number of experiments in the presence of nucleophiles (see the Supporting Information).^19^ With diol **2** **b** as a substrate, these experiments only resulted in the formation of tetrahydrofuran **3** **b**. However, when a model alcohol with identical electronic properties that is unable to undergo intramolecular cyclization, namely 1‐(*p*‐methoxyphenyl)‐1‐pentanol (**13** **b**), was subjected to the same reaction conditions, this substrate did react with the added nucleophiles (e.g., MeOH, 5 equiv). This result clearly supports the idea of carbocationic intermediates in the cyclization of diol **2** **b**.

## Conclusions

We have reported the acid‐ and base‐free cyclodehydration of 1,4‐ and 1,5‐diols catalyzed by NHC–iridium(III) complex **1** **a**. Supported by Hammett studies, KIE investigations, and crossover and trapping experiments, we found that the mechanism of the cyclization is highly dependent on the electronic properties of the diol substrates. Very electron‐rich aromatic substrates follow an acid‐catalyzed mechanistic pathway, whereas substrates with either no substituents or electron‐withdrawing substituents on the aromatic ring follow a hydrogen‐transfer mechanism. Both mechanisms may be operating simultaneously for moderately electron‐rich substrates. From a synthetic point of view, the protocol reported here, using bifunctional NHC–iridium(III) complexes, can be used for the preparation of functionalized 2,6‐disubstituted dihydropyran or 2,5‐disubstituted tetrahydrofuran building blocks from diols under neutral reaction conditions.

## Experimental Section

### Synthesis of 1,4‐diols

Commercially available 1,4‐diols **2** **k** and **l** were purchased from Sigma‐Aldrich and used as received. Non‐commercially available 1,4‐diols were obtained by reduction of 1,4‐diketone precursors. Commercially available 1,4‐diketones **5** **a** and **j**, precursors of 1,4‐diols **2** **a** and **j**, respectively, were purchased from Sigma‐Aldrich and used as received. Non‐commercially available 1,4‐diketones **5** were synthesized following reported procedures:

**Synthetic route A**: Cu(OTf)_2_ (5 mol %), MnCl_2_ **⋅**4 H_2_O (5 mol %), 1,8‐diazabicyclo[5.4.0]undec‐7‐ene (DBU; 7.5 mmol, 1.5 equiv), and aqueous *tert*‐butyl hydroperoxide (TBHP; 20 mmol, 4 equiv, 70 % in water) were added to a round‐bottom flask, equipped with a condenser, that contained a mixture of the corresponding vinylarene **7** (5 mmol) and acetone (**8**, 30 mL) (see the Supporting Information). The reaction mixture was heated at reflux, and the reaction progress was monitored by TLC. When the reaction was complete, the mixture was diluted with CH_2_Cl_2_ (125 mL) and washed with water. The aqueous phase was further extracted with CH_2_Cl_2_. The combined organic phases were dried with MgSO_4_, filtered, and concentrated under vacuum. The residue was purified by column chromatography (petroleum ether/ethyl acetate, 9:1, v/v) to give the desired diketone **5**.^20^

**Synthetic route B**: In a sealed glass tube equipped with a stirrer bar, the corresponding benzaldehyde precursor **9** (0.09 mol), triethylamine (19.5 mL, 0.14 mol), methyl vinyl ketone (**10**, 0.09 mol), and 3‐ethyl‐5‐(2‐hydroxyethyl)‐4‐methylthiazolium bromide (**11**, 3.53 g, 0.014 mol) were mixed together (see the Supporting Information). The flask was heated in the cavity of a microwave reactor for 15 min (150 W, internal temperature=70 °C, internal pressure=60 psi). After this time, the resulting mixture was stirred with aqueous HCl (2 m, 10 mL) for 30 min. The mixture was extracted with EtOAc. The organic layers were washed with aqueous sodium bicarbonate and brine. The organic fractions were dried over Na_2_SO_4_, filtered, and concentrated to give a crude orange liquid. Column chromatography (cyclohexane/ethyl acetate, 3:1, v/v) gave the desired diketone **5**.^21^

### Synthesis of 1,5‐diols

1,5‐Diols **2** **m** and **n** were obtained by reduction of 1,5‐diketones **5** **m** and **n**, respectively. 1,5‐Diketone **5** **n** is commercially available and was used as received from Sigma‐Aldrich. The synthesis of 1,5‐diketone **5** **m** was carried out by following a reported procedure.^22^ Methyl vinyl ketone (**10**) and iodine were added to a solution of the corresponding silyl enol ether **12** **m** in acetonitrile. When the reaction was complete, methanol and sodium thiosulfate were added. The mixture was extracted with EtOAc, and the crude product was purified by column chromatography (petroleum ether : EtOAc, 9:1) to give 1,5‐diol **2** **m**.

### General procedure for the cyclodehydration of diols

An oven‐dried microwave vial containing complex **1** **a** (0.03 mmol) was flushed with a stream of argon. Toluene (2.6 mL), *tert*‐butanol (1 mL), and the corresponding diol **2** (1 mmol) were added. The reaction mixture was stirred and heated at reflux for 24 h. After this time, the mixture was cooled down. The yield was quantified by ^1^H NMR spectroscopic analysis of the crude mixture or after purification by column chromatography. For 1,5‐diol substrates **2** **m** and **n**, an additional independent hydrogenation step with Pd/C was carried out to give the tetrahydropyrans (see the Supporting Information).

### General procedure for NMR‐scale experiments

Iridium complex **1** **b** (0.045 mmol, 27.5 mg) and anhydrous, degassed CH_2_Cl_2_ (4 mL) were added to a vial that contained AgBF_4_ (0.0945 mmol, 18.4 mg). The reaction mixture was stirred for 2 h at room temperature. The mixture was filtered through a pad of Celite® to remove the AgCl precipitate, and the filtrate was distributed into 20 NMR tubes. The solvent was evaporated under vacuum, and the NMR tubes were stored under an inert atmosphere. [D_8_]toluene (0.2 mL), *tert*‐butanol (0.05 mL), and a stock solution of a 1,4‐diol **2** (0.075 mmol) were added to an NMR tube that contained complex **1** **a** (0.00225 mmol). The NMR tube was then put into an NMR spectrometer, which was preheated to 100 °C. ^1^H NMR spectra were recorded every 2 min.

## Conflict of interest

The authors declare no conflict of interest.

## Supporting information

As a service to our authors and readers, this journal provides supporting information supplied by the authors. Such materials are peer reviewed and may be re‐organized for online delivery, but are not copy‐edited or typeset. Technical support issues arising from supporting information (other than missing files) should be addressed to the authors.

Supplementary
