# Supplementary material for: Nonclassical Mechanism in the Cyclodehydration of Diols Catalyzed by a Bifunctional Iridium Complex
Source: Chemistry. 2019 Jan 23;25(10):2631–6. doi: 10.1002/chem.201805460 (PMC7379557; doi:10.1002/chem.201805460)
Supplement: Supplementary file 1 — Supplementary [file CHEM-25-2631-s001.pdf]

CHEMISTRY
A European Journal

Supporting Information

Nonclassical Mechanism in the Cyclodehydration of Diols Catalyzed by a Bifunctional Iridium Complex

Greco González Miera+,[a] Aitor Bermejo López+,[a] Elisa Martínez-Castro,[a] Per-Ola Norrby,[b] and Belén Martín-Matute*[a]

chem_201805460_sm_miscellaneous_information.pdf

SUPPORTING INFORMATION

Nonclassical mechanism in the cyclodehydration of diols catalyzed by a bifunctional iridium complex

Greco González Miera, #[a] Aitor Bermejo López, #[a] Elisa Martínez-Castro,[a] Per-Ola Norrby,[b] and Belén Martín-Matute*[a]

[a] Dr. G. González Miera, A. Bermejo López, Dr. E. Martínez-Castro, Prof. B. Martín-Matute

Department of Organic Chemistry

Stockholm University

Stockholm 10691, Sweden

E-mail: belen.martin.matute@su.se

[b] Prof. P.-O. Norrby,

Early Product Development, Pharmaceutical Sciences, IMED Biotech Unit, AstraZeneca, Gothenburg, Sweden.

# Equal contribution

e-mail: belen.martin.matute@su.se

Contents

1. General 3

2. Preparation of catalysts 4

3. Optimization of reaction conditions in the cyclodehydration of diols 5

4. General procedure (a) for the synthesis of 1,4-diols 6

5. General procedure (b) for the synthesis of 1,4-diols 7

6. General procedure (c) for the synthesis of 1,5-diols 8

7. Spectral data of synthesized substrates 9

8. General procedure (d) for the cyclodehydration of 1,4- and 1,5-diols 16

9. Spectral data of synthesized products 16

10. Identification of oxidized linear products 26

11. Study of the transient products in the cyclodehydration of diols catalyzed by **1a** 27

12. Oxidative process of unsaturated 1,4-diols 27

13. Mechanistic investigations 28

14. Carbocation trapping experiments with nucleophiles 31

15. NMR spectra of synthesized substrates and products 38

1. General

All reactions were carried out under an atmosphere of argon in oven-dried Biotage® microwave vials unless otherwise specified. Reagents were of analytical grade, obtained from commercial suppliers and used as purchased. Anhydrous toluene and dichloromethane were obtained using a VAC solvent purification system. Flash chromatography was carried out on Davisil 60 Å (35-70 μm) silica gel. Analytical TLC was performed on aluminum plates pre-coated (0-25 mm) with silica gel (Merck, Silica Gel 60 F254). Compounds were detected by exposure to UV light or by revealing the plates in a solution of 5% $KMnO_4$ in water. Nuclear magnetic resonance (NMR) spectra were recorded at 400 or 500 MHz for ^{1}H NMR, and at 100 or 125 MHz for ^{13}C NMR, on a Bruker 400 or on a Bruker AV 500 spectrometer, respectively. ^{1}H and ^{13}C NMR chemical shifts (δ) are reported in ppm relative to the residual non-deuterated solvent peaks (Chloroform-*d₁*: δ_H 7.26 (s) ppm, and δ_C 77.0 (t) ppm. Acetone-d_6: δ_H 2.05 (quint) ppm, and δ_C 206.3 (m) and 29.9 (sept) ppm. Toluene-d_8: δ_H 7.09 (m), 7.01 (s), 6.97 (m), 2.08 (quin) ppm, and δ_C 137.9 (s), 128.9 (t), 128.0 (1:1:1 t), 125.1 (1:1:1 t), 20.4 (sept) ppm. Methanol-d_4: δ_H 4.78 (s), 3.31 (quin) ppm, and δ_C 49.0 (sept) ppm). Coupling constants (*J*) are given in Hz. Data are reported as follows: chemical shift, multiplicity, coupling constants and integration. ^{1}H NMR spectra were recorded using a relaxation delay T1 = 5 s (important integral regions of spectra with T1 > 5 s were equal to integral regions when T1 = 5 s). High-resolution mass spectra (HRMS) were obtained on a Bruker MicroTOF ESI-TOF spectrometer. Low-resolution mass spectra (LRMS) were recorded on a Shimadzu GC-MS (EI) spectrometer.

2. Preparation of catalysts

Complexes **1a**–**1c** were synthesized following procedures previously reported in the literature by our group.[1,2]

2.1. Complex 1a

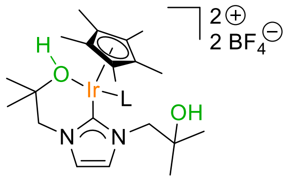

1a

^{1}H NMR (500 MHz, CD_3COCD_3, 248 K): 7.76 (d, 1 H, *J* = 1.8 Hz, NC*H*CHN$_{\text{imidazole backbone}}$), 7.64 (d, 1 H, *J* = 1.8 Hz, NCHC*H*N$_{\text{imidazole backbone}}$), 4.69 (d, 1 H, *J* = 14.2 Hz, NC*H*HC(OH)$(CH_3)_2$), 4.45 (d, 1 H, *J* = 14.9 Hz, NCH*H*C(OH)$(CH_3)_2$), 4.27 (d, 1 H, *J* = 14.2 Hz, NC*H*HC(OH)$(CH_3)_2$), 3.94 (d, 1 H, *J* = 14.9 Hz, NCH*H*C(OH)$(CH_3)_2$), 1.77 (s, 15 H, $C_5(C\mathit{H_3})_5$), 1.61 (s, 3 H, $NCH_2C(OH)(C\mathit{H_3})_2$), 1.49 (s, 3 H, $NCH_2C(OH)(C\mathit{H_3})_2$), 1.44 (s, 3 H, $NCH_2C(OH)(C\mathit{H_3})_2$), 0.94 (s, 3 H, $NCH_2C(OH)(C\mathit{H_3})_2$).

2.2. Complex 1b

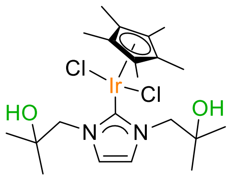

1b

^{1}H NMR (500 MHz, $CDCl_3$, 298 K): δ = 7.35 (s, 2 H, NC*H*C*H*N$_{\text{imidazole backbone}}$), 5.14 (d, 2 H, *J* = 13.5 Hz, NC*H*HC(OH)$(CH_3)_2$), 3.64 (d, 2 H, *J* = 13.4 Hz, NCH*H*C(OH)$(CH_3)_2$), 1.49 (s, 15 H, $C_5(C\mathit{H_3})_5$), 1.35 (s, 6 H, $NCH_2C(OH)(C\mathit{H_3})_2$), 1.31 (s, 6 H, $NCH_2C(OH)(C\mathit{H_3})_2$).

2.3. Complex 1c

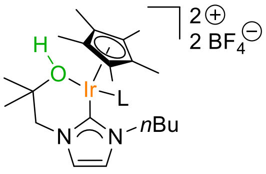

1c

^{1}H NMR (500 MHz, CD_3COCD_3, 298 K): δ = 7.73 (br s, 1 H, NC*H*CHN$_{\text{imidazole backbone}}$), 7.65 (br s, 1 H, NCHC*H*N$_{\text{imidazole backbone}}$), 4.49-4.46 (m, 1 H, NCH*H*C(OH)CH_3), 4.37-4.36 (m, 1 H, CH*H* $_{n\text{-butyl}}$), 4.22 (br s, 1 H, C*H*H $_{n\text{-butyl}}$), 4.01-3.98 (m, 1 H, NC*H*HC(OH)CH_3), 2.06-2.03 (m, 2 H, $C\mathit{H_2}$ $_{n\text{-butyl}}$), 1.83 (s, 15 H, $C_5(C\mathit{H_3})_5$), 1.57-1.53 (m, 2 H, $C\mathit{H_2}$ $_{n\text{-butyl}}$), 1.10 (s, 3 H, NCHHC(OH)$C\mathit{H_3}$), 1.03 (t, 3 H, *J* = 7.5 Hz, $C\mathit{H_3}$ $_{n\text{-butyl}}$).

3. Optimization of reaction conditions in the cyclodehydration of diols

Table S1. Catalyst scope for the cyclodehydration of **2b**.[a]

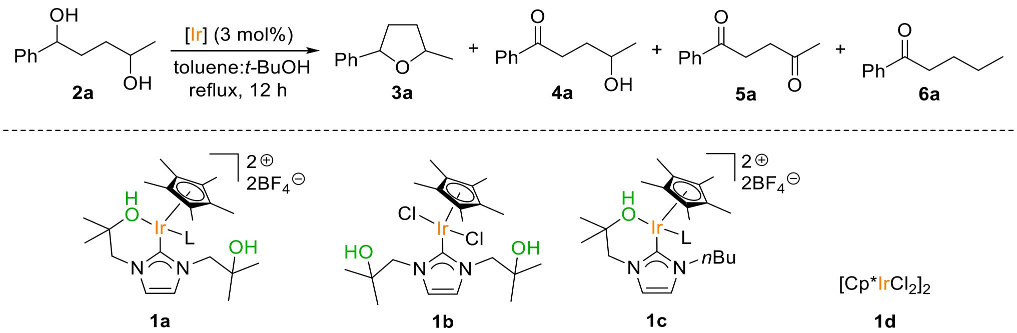

Entry	[Ir]	**3b** (%)[b]	**4b** (%)[b]	**5b** (%)[b]	**6b** (%)[b]	**7b** (%)[b]
1	**1a**	<1	91	5	4	<1
2	**1b**	20	<1	45	13	23
3	**1c**	8	31	30	13	18
4	**1d**	10	17	26	12	35
5	**-**	>99	<1	<1	<1	<1
6[c]	**1a**	14	70	3	14	<1

[a] Reaction conditions: diol (0.5 mmol), [Ir] (0.015 mmol, 3 mol%), toluene (1.3 mL), *tert*-butanol (0.5 mL), reflux, 12 h. [b] Yield determined by ^{1}H NMR spectroscopy. [c] Reaction conditions: diol (0.5 mmol), [Ir] (0.015 mmol, 3 mol%), toluene (1.8 mL), reflux, 12 h.

4. General procedure (a) for the synthesis of 1,4-diols

The synthesis of 1,4-diols **2c**, **2d**, **2f** and **2i** was performed following a reported procedure.[3] $Cu(OTf)_2$ (5 mol%), $MnCl_2{\cdot}4H_2O$ (5 mol%), 1,8-diazabicyclo[5.4.0]undec-7-ene (DBU, 7.5 mmol, 1.5 equiv) and aqueous *tert*-butyl hydroperoxide (TBHP, 20 mmol, 4 equiv, 70% in water) were added to a round bottom flask equipped with a condenser containing a mixture of the corresponding vinylarene **7** (5 mmol) and acetone (**8**, 30 mL). The reaction was stirred at reflux and monitored by TLC. After completion, the reaction mixture was diluted by using dichloromethane (125 mL). After extraction, the combined organic phases were dried over $MgSO_4$, filtered off, concentrated under vacuum, and the residue was purified by column chromatography using petroleum ether and ethyl acetate (9:1 v/v) as eluent. The resulting diketone **5** (1 equiv) was added to a flask containing ethanol (95%) in an ice bath at 0 °C. $NaBH_4$ (5 equiv) was added and the mixture was kept stirring at 0 °C for 1 h. After that time, the ice bath was removed and the mixture was stirred at room temperature overnight. Water (40 mL) and aqueous HCl (1 M) were added to the mixture until pH 7 was reached. The resulting aqueous phase was washed with ethyl acetate (3 x 100 mL) and the combined organic phases were dried over $MgSO_4$ and filtered off. The organic solvent was removed under vacuum and the residue was purified by column chromatography using petroleum ether and ethyl acetate (9:1 v/v) as eluent. The yields obtained after the reduction were almost quantitative.

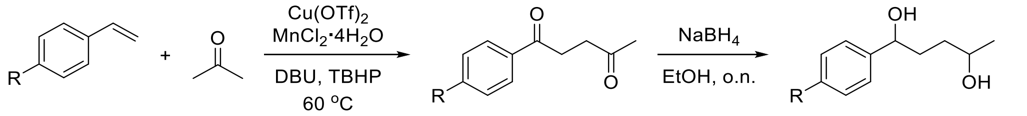

7c (R = *p(t*Bu)) **8** **5c** (R = *p*(*t*Bu)) **2c** (R = *p*(*t*Bu))
7d (R = *p*Me) **5d** (R = *p*Me) **2d** (R = *p*Me)
7f (R = *p*F) **5f** (R = *p*F) **2f** (R = *p*F)
7i (R = *p*(2-naphthyl)) **5i** (R = *p*(2-naphthyl)) **2i** (R = *p*(2-naphthyl))

Scheme S1. Synthesis of 1,4-diols **2c**, **2d**, **2f** and **2i**.

5. General procedure (b) for the synthesis of 1,4-diols

The synthesis of 1,4-diols **2b**, **2e**, **2g** and **2h** was performed following a published protocol.[4] In a sealed glass tube equipped with a stirring bar, the corresponding precursor benzaldehyde (**9**, 0.09 mol), triethylamine (19.5 mL, 0.14 mol), methyl vinyl ketone (**10**, 0.09 mol), and 3-ethyl-5-(2-hydroxyethyl)-4-methylthiazolium bromide (**11**, 3.53 g, 0.014 mol) were mixed together. The flask was heated in the cavity of a microwave reactor for 15 min (150 W, internal temperature 70 °C, and internal pressure 60 psi). At the end, the obtained crude residue was stirred with 10 ml of aqueous HCl (2 M) for 30 min. After extraction with ethyl acetate, the organic layers were washed with aqueous sodium bicarbonate and brine. Following the second extraction, the combined organic phases were dried over $MgSO_4$, filtered off, concentrated under vacuum, and the residue was purified by column chromatography using cyclohexane and ethyl acetate (3:1 v/v) as eluent. The resulting diketone **5** (1 equiv) was added to a flask containing ethanol (95%) in an ice bath at 0 °C. $NaBH_4$ (5 equiv) was added and the mixture was kept stirring at 0 °C for 1 h. After that time, the ice bath was removed and the mixture was stirred at room temperature overnight. Water (40 mL) and aqueous HCl (1 M) were added to the mixture until pH 7 was reached. The resulting aqueous phase was washed with ethyl acetate (3 x 100 mL) and the combined organic phases were dried over $MgSO_4$ and filtered off. The organic solvent was removed under vacuum and the residue was purified by column chromatography using petroleum ether and ethyl acetate (9:1 v/v) as eluent. The yields obtained after the reduction were almost quantitative.

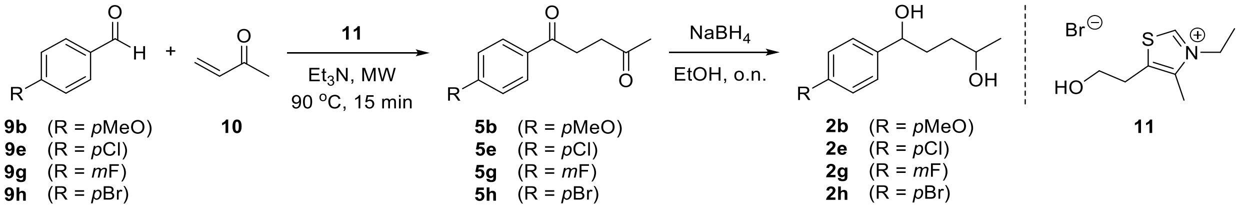

Scheme S2. Synthesis of 1,4-diketones **5b**, **5e**, **5g** and **5h**.

6. General procedure (c) for the synthesis of 1,5-diols

1,5-Diketone **5n** is commercially available and was used as received. The synthesis of 1,5-diketone **5m** was performed in accordance to a reported procedure.[5] A solution of 1-phenyl-1-trimethylsiloxyethylene (**12**, 1.1 mmol) in acetonitrile was added to a solution of methyl vinyl ketone (**10**, 1 mmol) and iodine (0.1 mmol) and stirred at room temperature. After completion of the reaction monitored by TLC, methanol and sodium thiosulfate were added consecutively. The mixture was extracted with ethyl acetate, and the crude product was purified by column chromatography. The corresponding diketone **5** (1 equiv) was added to a flask containing ethanol (95%) in an ice bath at 0 °C. $NaBH_4$ (5 equiv) was added and the mixture was kept stirring at 0 °C for 1 h. After that time, the ice bath was removed and the mixture was stirred at room temperature overnight. Water (40 mL) and aqueous HCl (1 M) were added to the mixture until pH 7 was reached. The resulting aqueous phase was washed with ethyl acetate (3 x 100 mL) and the combined organic phases were dried over $MgSO_4$ and filtered off. The organic solvent was removed under vacuum and the residue was purified by column chromatography using petroleum ether and ethyl acetate (9:1 v/v) as eluent. The yields obtained after the reduction were almost quantitative for 1,5-diol **2m**.

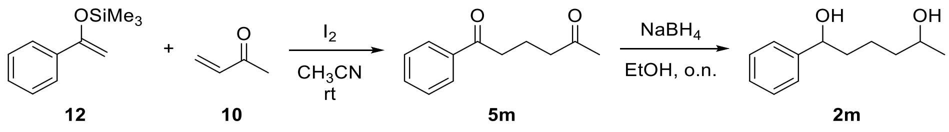

Scheme S3. Synthesis of 1,5-diol **2m**.

7. Spectral data of synthesized substrates

7.1. 1-Phenylpentane-1,4-diol (2a)

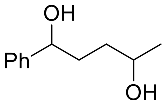

2a

Diol **2a** was obtained from the reduction of the commercially available 1-phenylpentane-1,4-dione **5a** with $NaBH_4$. The product was purified by column chromatography using petroleum ether and ethyl acetate (9:1 v/v) (dr: 0.7:1). The spectroscopy data was in accordance with the data reported in the literature.[6]

^{1}H NMR (400 MHz, $CDCl_3$, 298 K, mixture of two isomers): δ = 7.38-7.27 (m, 5 H, Ar (both diast.)), 4.77-4.71 (m, 1 H, ArC*H*OH (both diast.)), 3.93-3.83 (m, 1 H, C*H*(OH)CH_3 (both diast.)), 2.19 (br s, 2 H, CHO*H* (both diast.)), 1.96-1.82 (m, 2 H, CH_2 (both diast.)), 1.69-1.46 (m, 2 H, CH_2 (both diast.)), 1.23 (d, 3 H, *J* = 6.2 Hz, CH_3 (one diast.)), 1.22 (d, 3 H, *J* = 6.2 Hz, CH_3 (one diast.)).

7.2. 1-(*p*-Methoxyphenyl)pentane-1,4-diol (2b)

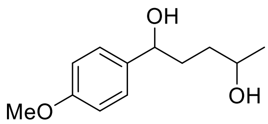

2b

The general procedure (b) was applied using *p*-anisaldehyde **9b**. Product **2b** was purified by column chromatography using petroleum ether and ethyl acetate (9:1 v/v) (dr: 1:1). The spectroscopy data was in accordance with the data reported in the literature.[3]

^{1}H NMR (400 MHz, $CDCl_3$, 298 K, mixture of two isomers): δ = 7.28-7.26 (m, 2 H, Ar (both diast.)), 6.90-6.86 (m, 2 H, Ar (both diast.)), 4.69-4.64 (m, 1 H, ArC*H*OH (both diast.)), 3.88-3.79 (m, 1 H, C*H*(OH)CH_3 (both diast.)), 3.80 (s, 3 H, CH_3 (both diast.)), 2.21 (br s, 2 H, CHO*H* (both diast.)), 1.93-1.76 (m, 2 H, CH_2 (both diast.)), 1.65-1.41 (m, 2 H, CH_2 (both diast.)), 1.19 (d, 3 H, *J* = 6.2 Hz, CH_3 (both diast.)).

^{13}C NMR (100 MHz, $CDCl_3$, 298 K, mixture of two isomers): δ = 158.9, 136.9, 127.2, 127.0, 126.9, 113.8, 113.7, 80.8, 80.0, 74.3, 73.9, 68.2, 67.8, 55.3, 36.0, 35.6, 35.0, 34.3, 33.1, 23.7, 23.4, 21.6, 21.4.

7.3. 1-(*p*-*tert*-Butylphenyl)pentane-1,4-diol (2c)

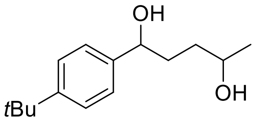

2c

The general procedure (a) was applied using 1-(*tert*-butyl)-4-vinylbenzene **7c**. Product **2c** was purified by column chromatography using petroleum ether and ethyl acetate (9:1 v/v) (dr: 1:1).

^{1}H NMR (400 MHz, $CDCl_3$, 298 K, mixture of two isomers): δ = 7.38 (d, 2 H, *J* = 8.2 Hz, Ar (both diast.)), 7.29 (d, 2 H, *J* = 8.3 Hz, Ar (both diast.)), 4.72-4.66 (m, 1 H, ArC*H*OH (both diast.)), 3.92-3.81 (m, 1 H, C*H*(OH)CH_3 (both diast.)), 2.50 (br s, 2 H, CHO*H* (both diast.)), 1.93-1.83 (m, 2 H, CH_2 (both diast.)), 1.69-1.46 (m, 2 H, CH_2 (both diast.)), 1.34 (s, 9 H, *J* = 6.4 Hz, (CH_3)$_3$ (both diast.)), 1.20 (d, 3 H, *J* = 6.2 Hz, CH_3 (both diast.)).

^{13}C NMR (100 MHz, $CDCl_3$, 298 K, mixture of two isomers): δ = 150.4, 150.4, 141.8, 141.7, 125.5, 125.34, 74.6, 74.2, 68.2, 67.9, 36.1, 35.9, 35.3, 35.0, 31.4, 23.7, 23.5.

HRMS-ESI calcd for $C_{15}H_{24}O_2Na$ [M+Na]$^+$: 259.1674, found: 259.1328.

7.4. 1-(*p*-Tolyl)pentane-1,4-diol (2d)

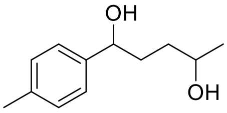

2d

The general procedure (a) was applied using 1-methyl-4-vinylbenzene **7d**. Product **2d** was purified by column chromatography using petroleum ether and ethyl acetate (9:1 v/v) (dr: 1:0.67).

^{1}H NMR (400 MHz, $CDCl_3$, 298 K, mixture of two isomers): δ = 7.25 (d, 2 H, *J* = 8.0 Hz, Ar (both diast.)), 7.17 (d, 2 H, *J* = 7.8 Hz, Ar (both diast.)), 4.72-4.65 (m, 1 H, ArC*H*OH (both diast.)), 3.91-3.80 (m, 1 H, C*H*(OH)CH_3 (both diast.)), 2.46 (s, 2 H, CHO*H* (both diast.)), 2.36 (s, 3 H, ArCH_3 (both diast.)), 1.93-1.78 (m, 2 H, CH_2 (both diast.)), 1.66-1.43 (m, 2 H, CH_2 (both diast.)), 1.20 (d, 3 H, *J* = 6.1 Hz, CH_3 (one diast.)), 1.19 (d, 3 H, *J* = 6.2 Hz, CH_3 (one diast.)).

^{13}C NMR (100 MHz, $CDCl_3$, 298 K, mixture of two isomers): δ = 141.9, 137.1, 129.1, 125.8, 125.7, 125.3, 74.7, 74.2, 69.2, 67.9, 36.0, 35.1, 31.4, 23.7, 23.4, 21.1.

HRMS-ESI calcd for $C_{12}H_{18}O_2Na$ [M+Na]$^+$: 217.1204, found: 217.1171.

7.5. 1-(*p*-Chlorophenyl)pentane-1,4-diol (2e)

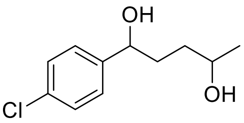

2e

The general procedure (b) was applied using *p*-chlorobenzaldehyde **9e**. Product **2e** was purified by column chromatography using petroleum ether and ethyl acetate (9:1 v/v) (dr: 0.97:1).

^{1}H NMR (400 MHz, $CDCl_3$, 298 K, mixture of two isomers): δ = 7.34-7.29 (m, 4 H, Ar (both diast.)), 4.75-4.69 (m, 1 H, ArC*H*OH (both diast.)), 3.93-3.84 (m, 1 H, C*H*(OH)CH_3 (both diast.)), 2.37 (s, 2 H, CHO*H* (both diast.)), 1.89-1.82 (m, 2 H, CH_2 (both diast.)), 1.66-1.43 (m, 2 H, CH_2 (both diast.)), 1.22 (d, 3 H, *J* = 6.2 Hz, CH_3 (one diast.)), 1.21 (d, 3 H, *J* = 6.2 Hz, CH_3 (one diast.)).

^{13}C NMR (100 MHz, $CDCl_3$, 298 K, mixture of two isomers): δ = 143.4, 143.2, 133.0, 128.5, 127.2, 127.1, 74.0, 73.6, 69.3, 67.9, 36.2, 35.7, 35.2, 34.8, 23.8, 23.6.

HRMS-ESI calcd for $C_{11}H_{15}O_2ClNa$ $[M+Na]^+$: 237.0658, found: 237.0811.

7.6. 1-(*p*-Fluorophenyl)pentane-1,4-diol (2f)

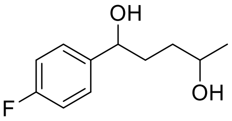

2f

The general procedure (a) was applied using 1-fluoro-4-vinylbenzene **7f**. Product **2f** was purified by column chromatography using petroleum ether and ethyl acetate (9:1 v/v) (dr: 0.89:1). The spectroscopy data was in accordance with the data reported in the literature.[3]

^{1}H NMR (400 MHz, $CDCl_3$, 298 K, mixture of two isomers): δ = 7.34-7.29 (m, 4 H, Ar (both diast.)), 4.75-4.68 (m, 1 H, ArC*H*OH (both diast.)), 3.93-3.82 (m, 1 H, C*H*(OH)CH_3 (both diast.)), 2.36 (br s, 2 H, CHO*H* (both diast.)), 1.89-1.82 (m, 2 H, CH_2 (both diast.)), 1.66-1.43 (m, 2 H, CH_2 (both diast.)), 1.22 (d, 3 H, *J* = 6.2 Hz, CH_3 (one diast.)), 1.21 (d, 3 H, *J* = 6.2 Hz, CH_3 (one diast.)).

^{13}C NMR (100 MHz, $CDCl_3$, 298 K, mixture of two isomers): δ = 183.2(d, $J(^{13}C, ^{19}F)$ = 2.2 Hz), 160.8 (d, $J(^{13}C, ^{19}F)$ = 2.2 Hz), 140.7 (d, $J(^{13}C, ^{19}F)$ = 3.1 Hz), 140.4 (d, $J(^{13}C, ^{19}F)$ = 3.1 Hz), 127.5 (d, $J(^{13}C, ^{19}F)$ = 3.6 Hz), 127.4 (d, $J(^{13}C, ^{19}F)$ = 3.6 Hz), 115.2, 73.9, 73.3, 68.2, 67.6, 36.3, 35.9, 35.0, 34.7, 23.6, 23.2.

7.7. 1-(*m*-Fluorophenyl)pentane-1,4-diol (2g)

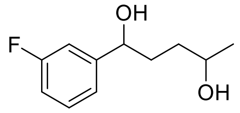

2g

The general procedure (b) was applied using *m*-fluorobenzaldehyde **9g**. Product **2g** was purified by column chromatography using petroleum ether and ethyl acetate (9:1) (dr: 1:0.98).

^{1}H NMR (400 MHz, $CDCl_3$, 298 K, mixture of two isomers): δ = 7.33-7.27 (m, 1 H, Ar (both diast.)), 7.12-7.07 (m, 2 H, Ar (both diast.)), 6.98-6.93 (m, 1 H, Ar (both diast.)), 4.75-4.67 (m, 1 H, ArC*H*OH (both diast.)), 3.91-3.80 (m, 1 H, C*H*(OH)CH_3 (both diast.)), 3.06 (br s, 2 H, CHO*H* (both diast.)), 1.89-1.81 (m, 2 H, C*H₂* (both diast.)), 1.65-1.43 (m, 2 H, C*H₂* (both diast.)), 1.21-1.18 (m, 3 H, C*H₃* (both diast.)).

^{13}C NMR (100 MHz, $CDCl_3$, 298 K, mixture of two isomers): δ = 164.2 (d, $J(^{13}C, ^{19}F)$ = 53.7 Hz), 161.7 (d, $J(^{13}C, ^{19}F)$ = 1.7 Hz), 147.7 (d, $J(^{13}C, ^{19}F)$ = 6.6 Hz), 147.5 (d, $J(^{13}C, ^{19}F)$ = 6.6 Hz), 129.9(d, $J(^{13}C, ^{19}F)$ = 8.1 Hz), 121.4 (d, $J(^{13}C, ^{19}F)$ = 1.2 Hz), 121.3 (d, $J(^{13}C, ^{19}F)$ = 1.2 Hz), 114.2 (d, $J(^{13}C, ^{19}F)$ = 4.1 Hz), 114.1 (d, $J(^{13}C, ^{19}F)$ = 4.1 Hz), 112.9 (d, $J(^{13}C, ^{19}F)$ = 4.3 Hz), 112.7 (d, $J(^{13}C, ^{19}F)$ = 4.3 Hz), 74.0 (d, $J(^{13}C, ^{19}F)$ = 1.7 Hz), 73.4 (d, $J(^{13}C, ^{19}F)$ = 1.7 Hz), 69.3, 67.9, 36.2, 35.8, 35.1, 34.7, 23.7, 23.5.

HRMS-ESI calcd for $C_{11}H_{15}O_2FNa$ $[M+Na]^+$: 221.0954, found: 221.1346.

7.8. 1-(*p*-Bromophenyl)pentane-1,4-diol (2h)

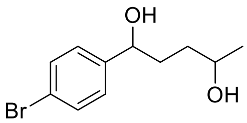

2h

The general procedure (b) was applied using *p*-bromobenzaldehyde **9h**. Product **2h** was purified by column chromatography using petroleum ether and ethyl acetate (9:1 v/v) (dr: 0.90:1). The spectroscopy data was in accordance with the data reported in the literature.[3]

^{1}H NMR (400 MHz, $CDCl_3$, 298 K, mixture of two isomers): δ = 7.48-7.46 (m, 2 H, Ar (both diast.)), 7.25-7.22 (m, 2 H, Ar (both diast.)), 4.73-4.67 (m, 1 H, ArC*H*OH (both diast.)), 3.92-3.82 (m, 1 H, C*H*(OH)CH_3 (both diast.)), 2.56 (br s, 2 H, CHO*H* (both diast.)), 1.87-1.81 (m, 2 H, C*H₂* (both diast.)), 1.65-1.42 (m, 2 H, C*H₂* (both diast.)), 1.21 (d, 3 H, *J* = 6.2 Hz, C*H₃* (one diast.)), 1.20 (d, 3 H, *J* = 6.2 Hz, C*H₃* (one diast.)).

^{13}C NMR (100 MHz, $CDCl_3$, 298 K, mixture of two isomers): δ = 143.7, 131.5, 127.5, 121.1, 74.1, 73.6, 69.3, 67.9, 36.1, 35.7, 35.2, 34.8, 23.8, 23.6.

7.9. 1-(2-Naphthalenyl)-1,4-pentanediol (2i)

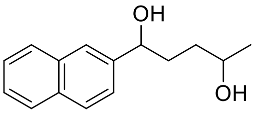

2i

The general procedure (a) was applied using 2-vinylnaphthalene **7i**. Product **2i** was purified by column chromatography using petroleum ether and ethyl acetate (9:1 v/v) (dr: 1:0.90). The spectroscopy data was in accordance with the data reported in the literature.[3]

^{1}H NMR (400 MHz, $CDCl_3$, 298 K, mixture of two isomers): δ = 7.84-7.80 (m, 4 H, Ar (both diast.)), 7.50-7.44 (m, 3 H, Ar (both diast.)), 4.93-4.86 (m, 1 H, ArC*H*OH (both diast.)), 3.93-3.83 (m, 1 H, C*H*(OH)CH_3 (both diast.)), 2.82 (br s, 1 H, CHO*H* (one diast.)), 2.59 (br s, 1 H, CHO*H* (one diast.)), 2.08 (br s, 1 H, CHO*H* (one diast.)), 2.01-1.80 (m, 2 H, CH_2 (both diast.)), 1.82 (br s, 1 H, CHO*H* (one diast.)), 1.70-1.46 (m, 2 H, CH_2 (both diast.)), 1.21 (d, 3 H, J = 6.2 Hz, CH_3 (one diast.)), z1.20 (d, 3 H, J = 6.2 Hz, CH_3 (one diast.)).

^{13}C NMR (100 MHz, $CDCl_3$, 298 K, mixture of two isomers): δ = 142.2, 133.3, 133.0, 128.2, 128.0, 127.7, 126.1, 125.8, 124.5, 124.4, 124.0, 74.9, 74.5, 68.3, 68.8, 35.9, 35.1, 23.8, 23.6.

7.10. 1,4-Diphenylbutane-1,4-diol (2j)

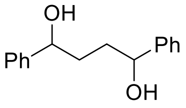

2j

Diol **2j** was obtained from the reduction of the commercially available 1,4-diphenylbutane-1,4-dione **5j** with $NaBH_4$. The product was purified by column chromatography using petroleum ether and ethyl acetate (9:1 v/v). The spectroscopy data was in accordance with the data reported in the literature.[7]

^{1}H NMR (400 MHz, $CDCl_3$, 298 K): δ = 7.38-7.24 (m, 10 H, Ar), 4.78-4.69 (m, 2 H, ArC*H*OH), 2.27 (s, 2 H, CHO*H*), 1.98-1.78 (m, 4 H, CH_2).

^{13}C NMR (100 MHz, $CDCl_3$, 298 K): δ = 144.5, 128.5, 127.5, 125.8, 74.7, 74.3, 35.9, 35.2.

7.11. 1-Phenylhexane-1,5-diol (2m)

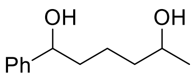

2m

The general procedure (c) was applied using trimethylvinyloxysilane **9m**. Product **2m** was purified by column chromatography using petroleum ether and ethyl acetate (9:1 v/v) (dr: 1:1).

^{1}H NMR (400 MHz, $CDCl_3$, 298 K, mixture of two isomers): δ = 7.37-7.27 (m, 5 H, Ar (both diast.)), 4.70-4.66 (m, 1 H, ArC*H*OH (both diast.)), 3.82-3.74 (m, 1 H, C*H*(OH)CH_3 (both diast.)), 1.88-1.62 (m, 4 H, CH_2 (both diast.), CHO*H* (both diast.)), 1.55-1.23 (m, 4 H, CH_2 (both diast.), CH_2 (both diast.)), 1.17 (d, 3 H, J = 6.2 Hz, CH_3 (both diast.)).

^{13}C NMR (100 MHz, $CDCl_3$, 298 K, mixture of two isomers): δ = 144.8, 144.7, 128.7, 127.6, 125.9, 125.8, 74.5, 67.9, 39.0, 38.9, 23.6, 23.5, 22.1, 22.0.

HRMS-ESI calcd for $C_{12}H_{18}O_2Na$ $[M+Na]^+$: 217.1204, found: 217.1316.

7.12. 1,5-Diphenylpentane-1,5-diol (2n)

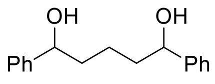

2n

Diol **2n** was obtained from the reduction of the commercially available 1,5-diphenylpentane-1,5-dione **5n** with $NaBH_4$. Product **2n** was purified by column chromatography using petroleum ether and ethyl acetate (9:1 v/v). The spectroscopy data was in accordance with the data reported in the literature.[8]

^{1}H NMR (400 MHz, $CDCl_3$, 298 K): δ = 7.38-7.29 (m, 10 H, Ar), 4.67-4.64 (m, 2 H, ArC*H*OH), 2.20 (s, 2 H, CHO*H*), 1.87-1.28 (m, 6 H, CH_2).

^{13}C NMR (100 MHz, $CDCl_3$, 298 K): δ = 144.8, 128.5, 127.5, 125.8, 74.4, 74.3, 38.8, 22.3, 22.1.

7.13. 1-Phenylpentane-1,4-d_2-1,4-diol (2a-d_2)

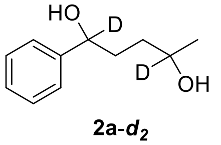

2a-d_2

Diol **2a-d_2** was obtained from the reduction of the commercially available 1-phenylpentane-1,4-dione **5a** with $NaBD_4$. The product was purified by column chromatography using petroleum ether and ethyl acetate (9:1 v/v) (dr: 1:0.94).

^{1}H NMR (400 MHz, $CDCl_3$, 298 K, mixture of two isomers): δ = 7.35-7.24 (m, 5 H, Ar (both diast.)), 2.61 (s, 2 H, CHO*H*), 1.90-1.77 (m, 2 H, CH_2 (both diast.)), 1.63-

1.42 (m, 2 H, CH_2 (both diast.)), 1.17 (s, 3 H, CH_3 (one diast.)), 1.16 (s, 3 H, CH_3 (one diast.)).

^{13}C NMR (100 MHz, $CDCl_3$, 298 K, mixture of two isomers): δ = 144.8, 144.7, 128.4, 127.5, 127.4, 125.8, 35.9, 74.06 (q, $J(^{13}C, ^{2}H)$ = 21.9 Hz), 67.4 (q, $J(^{13}C, ^{2}H)$ = 21.7 Hz), 34.9, 23.5, 23.3, 14.2.

HRMS-ESI calcd for $C_{11}H_{14}D_2O_2Na$ $[M+Na]^+$: 205.1174, found: 205.1173.

7.14. 1-(*p*-Methoxyphenyl)pentane-1,4-*d₂*-1,4-diol (2b-*d₂*)

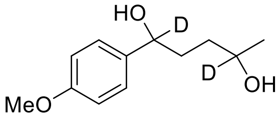

2b-*d₂*

Diol **2b-*d₂*** was obtained from the reduction of **5b** with $NaBD_4$. The product was purified by column chromatography using petroleum ether and ethyl acetate (9:1 v/v) (dr: 1:1).

^{1}H NMR (400 MHz, $CDCl_3$, 298 K, mixture of two isomers): δ = 7.28-7.26 (m, 2 H, Ar (both diast.)), 6.89-6.87 (m, 2 H, Ar (both diast.)), 3.80 (s, 3 H, OCH_3 (both diast.)), 1.91-1.77 (m, 2 H, CH_2 (both diast.)), 1.63-1.42 (m, 2 H, CH_2 (both diast.)), 1.19 (s, 3 H, CH_3 (both diast.)).

^{13}C NMR (100 MHz, $CDCl_3$, 298 K, mixture of two isomers): δ = 159.1, 136.8, 127.1, 127.1, 113.9, 55.3, 35.8 (d, $J(^{13}C, ^{2}H)$ = 20.6 Hz), 35.0 (d, $J(^{13}C, ^{2}H)$ = 20.9 Hz), 23.5 (d, $J(^{13}C, ^{2}H)$ = 21.9 Hz).

HRMS-ESI calcd for $C_{12}H_{16}D_2O_3Na$ $[M+Na]^+$: 235.1279, found: 235.1286.

8. General procedure (d) for the cyclodehydration of 1,4- and 1,5-diols

A microwave vial impregnated with **1a** (0.03 mmol) and flushed with a current of argon was loaded with toluene (2.6 mL), *tert*-butanol (1 mL) and the corresponding diol (**2**, 1 mmol). The reaction mixture was stirred and refluxed for 24 h. After completion of the reaction time, the mixture was cooled down and the yield was quantified by ^{1}H NMR or after purification by column chromatography.

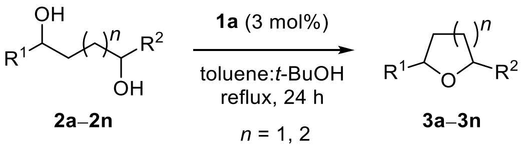

Scheme S4. Cyclodehydration of diols catalyzed by **1a**.

9. Spectral data of synthesized products

9.1. 2-Phenyl-5-methyltetrahydrofuran (3a)

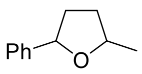

3a

The general procedure (d) was applied using 1-phenyl-1,4-pentanediol (**2a**, 180 mg, 1 mmol). The product was purified by column chromatography using petroleum ether and ethyl acetate (20:1 v/v) as eluent. (101 mg, 0.62 mmol). The spectroscopic data was in accordance with the data reported in the literature.[3]

Yield: 62%, dr: 0.45:1

^{1}H NMR (400 MHz, $CDCl_3$, 298 K, mixture of two isomers): δ = 7.39-7.23 (m, 5 H, Ar *cis+trans* isomers), 5.06 (dd, 1 H, *J* = 8.2, 6.5 Hz, H-5 *trans* isomer), 4.89 (t, 1 H, *J* = 7.3 Hz, H-5 *cis* isomer), 4.41-4.33 (m, 1 H, H-2 *trans* isomer), 4.23-4.15 (m, 1 H, H-2 *cis* isomer), 2.44-2.36 (m, 1 H, H-4 *trans* isomer), 2.34-2.27 (m, 1 H, H-4 *cis* isomer), 2.21-2.15 (m, 1 H, H-4 *trans* isomer), 2.13-2.06 (m, 1 H, H-4 *cis* isomer), 1.94-1.83 (m, 1 H, H-3 *cis+trans* isomers), 1.68-1.58 (m, 1 H, H-3 *cis+trans* isomers), 1.39 (d, 3 H, *J* = 6.1 Hz, CH_3 *cis* isomer), 1.34 (d, 3 H, *J* = 6.1 Hz, CH_3 *trans* isomer).

^{13}C NMR (100 MHz, $CDCl_3$, 298 K): δ = 144.1, 143.6, 128.4, 128.4, 127.2, 127.1, 126.0, 125.7, 81.2, 80.4, 76.8, 76.1, 76.0, 35.7, 34.8, 34.4, 33.2, 21.7, 21.5.

9.2. 2-(*p*-Methoxyphenyl)-5-methyltetrahydrofuran (3b)

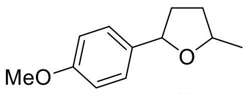

3b

The general procedure (d) was applied using 1-(4-methoxyphenyl)pentane-1,4-diol (**2b**, 210 mg, 1 mmol). The product was purified by column chromatography using petroleum ether and ethyl acetate (20:1 v/v) as eluent. (173 mg, 0.90 mmol). The spectroscopic data was in accordance with the data reported in the literature.[9]

Yield: 90%, dr: 0.86:1

^{1}H NMR (400 MHz, $CDCl_3$, 298 K, mixture of two isomers): δ = 7.31-7.27 (m, 2 H, Ar *cis*+*trans* isomers), 6.90-6.86 (m, 2 H, Ar *cis*+*trans* isomers), 4.99 (dd, 1 H, J = 8.3, 6.3 Hz, H-2 *trans* isomer), 4.83 (t, 1 H, J = 7.3 Hz, H-2 *cis* isomer), 4.38-4.31 (m, 1 H, H-5 *trans* isomer), 4.18−4.14 (m, 1 H, H-5 *cis* isomer), 3.80 (s, 3 H, OCH_3 *trans* isomer), 3.80 (s, 3 H, OCH_3 *cis* isomer), 2.37-2.32 (m, 1 H, H-4 *trans* isomer), 2.29-2.22 (m, 1 H, H-4 *cis* isomer), 2.20-2.14 (m, 1 H, H-4 *trans* isomer), 2.13-2.06 (m, 1 H, H-4 *cis isomer*), 1.91-1.80 (m, 1 H, H-3 *cis*+*trans* isomers), 1.66-1.58 (m, 1 H, H-3 *cis*+*trans* isomers), 1.37 (d, 3 H, J = 6.1 Hz, CH_3 *cis* isomer), 1.32 (d, 3 H, J = 6.1 Hz, CH_3 *trans* isomer).

^{13}C NMR (100 MHz, $CDCl_3$, 298 K): δ = 158.9, 158.8, 136.0, 135.5, 127.2, 127.2, 127.0, 114.0, 113.8, 114.0, 113.7, 113.7, 100.0, 80.9, 80.0, 75.8, 75.7, 55.3, 35.6, 34.6, 34.4, 33.2, 29.8, 21.7, 21.5.

9.3. 2-(*p*-*tert*-Butylphenyl)-5-methyltetrahydrofuran (3c)

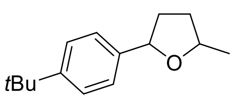

3c

The general procedure (d) was applied using 1-(4-*tert*-butylphenyl)pentane-1,4-diol (**2c**, 236 mg, 1 mmol). The product was purified by column chromatography using petroleum ether and ethyl acetate (20:1 v/v) as eluent. (184 mg, 0.84 mmol).

Yield: 84%, dr: 0.47:1

^{1}H NMR (400 MHz, $CDCl_3$, 298 K, mixture of two isomers): δ = 7.38-7.35 (m, 2 H, Ar *cis*+*trans* isomers), 7.32-7.26 (m, 2 H, Ar *cis*+*trans* isomers), 5.05-5.01 (m, 1 H, H-2 *trans* isomer), 4.86 (t, 1 H, J = 7.3 Hz, H-2 *cis* isomer), 4.39-4.30 (m, 1 H, H-5 *trans* isomer), 4.20-4.12 (m, 1 H, H-5 *cis* isomer), 2.41-2.34 (m, 1 H, H-4 *trans* isomer), 2.33-2.24 (m, 1 H, H-4 *cis* isomer), 2.20-2.13 (m, 1 H, H-4 *trans* isomer), 2.14-2.06 (m, 1 H, H-4 *cis isomer*), 1.97-1.84 (m, 1 H, H-3 *cis*+*trans* isomers), 1.67-

1.58 (m, 1 H, H-3 *cis+trans* isomers), 1.37 (d, 3 H, J = 6.1 Hz, CH_3 *cis* isomer), 1.33-1.32 (m, 12H, CH_3 *trans* isomer, (CH_3)$_3$ *cis+trans* isomers).

^{13}C NMR (100 MHz, $CDCl_3$, 298 K, mixture of two isomers): δ = 156.9, 151.2, 151.1, 150.0, 137.4, 137.3, 129.5, 128.1, 125.7, 125.4, 114.2, 114.0, 80.9, 80.1, 75.9, 75.7, 38.5, 37.1, 35.1, 34.9, 34.6, 34.3, 33.2, 32.4, 31.4, 31.1, 23.8, 22.4, 21.4, 20.1.

HRMS-ESI calcd for $C_{15}H_{22}ONa$ [M+Na]$^+$: 241.1568, found: 241.1596.

9.4. 2-(*p*-Methylphenyl)-5-methyltetrahydrofuran (3d)

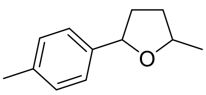

3d

The general procedure (d) was applied using 1-(4-methylphenyl)pentane-1,4-diol (**2d**, 194 mg, 1 mmol). The product was purified by column chromatography using petroleum ether and ethyl acetate (20:1 v/v) as eluent. (127 mg, 0.72 mmol). The spectroscopic data was in accordance with the data reported in the literature.[10]

Yield: 72%, dr: 0.52:1

^{1}H NMR (400 MHz, $CDCl_3$, 298 K, mixture of two isomers): δ = 7.28-7.23 (m, 2 H, Ar *cis+trans* isomers), 7.16-7.13 (m, 2 H, Ar *cis+trans* isomers), 5.04-5.01 (m, 1 H, H-2 *trans* isomer), 4.86 (t, 1 H, J = 7.3 Hz, H-2 *cis* isomer), 4.39-4.31 (m, 1 H, H-5 *trans* isomer), 4.21−4.13 (m, 1 H, H-5 *cis* isomer), 2.35 (s, 3 H, ArCH_3, *cis+trans* isomers), 2.42-2.24 (m, 1 H, H-4 *cis+trans* isomers), 2.19-2.14 (m, 1 H, H-4 *trans* isomers), 2.13-2.05 (m, 1 H, H-4 *cis isomer*), 1.91-1.80 (m, 1 H, H-3 *cis+trans* isomers), 1.67-1.56 (m, 1 H, H-3 *cis+trans* isomers), 1.37 (d, 3 H, J = 6.1 Hz, CH_3 *cis* isomer), 1.33 (d, 3 H, J = 6.3 Hz, CH_3 *trans* isomer).

^{13}C NMR (100 MHz, $CDCl_3$, 298 K, mixture of two isomers): δ = 140.9, 140.5, 136.7, 136.6, 130.0, 129.9, 125.8, 125.6, 81.0, 80.1, 75.9, 75.8, 35.6, 34.6, 34.3, 33.1, 31.4, 21.6, 21.4, 21.1.

9.5. 2-(*p*-Chlorophenyl)-5-methyltetrahydrofuran (3e)

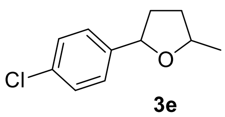

3e

The general procedure (d) was applied using 1-(4-chlorophenyl)pentane-1,4-diol (**2e**, 214 mg, 1 mmol). The product was purified by column chromatography using petroleum ether and ethyl acetate (9:1 v/v) as eluent. (148 mg, 0.75 mmol).

Yield: 75%, dr: 0.48:1

^{1}H NMR (400 MHz, $CDCl_3$, 298 K, mixture of two isomers): δ = 7.30-7.25 (m, 4 H, Ar *cis+trans* isomers), 5.00 (dd, 1 H, *J* = 8.1, 6.5 Hz, H-2 *trans* isomer), 4.84 (t, 1 H, *J* = 7.3 Hz, H-2 *cis* isomer), 4.37-4.29 (m, 1 H, H-5 *trans* isomer), 4.20-4.11 (m, 1 H, H-5 *cis* isomer), 2.41-2.34 (m, 1 H, H-4 *trans* isomer), 2.33-2.25 (m, 1 H, H-4 *cis* isomer), 2.18-2.10 (m, 1 H, H-4 *trans* isomer), 2.12-2.04 (m, 1 H, H-4 *cis* isomer), 1.85-1.74 (m, 1 H, H-3 *cis+trans* isomers), 1.66-1.54 (m, 1 H, H-3 *cis+trans* isomers), 1.36 (d, 3 H, *J* = 6.1 Hz, CH_3 *cis* isomer), 1.31 (d, 3 H, *J* = 6.1 Hz, CH_3 *trans* isomer).

^{13}C NMR (100 MHz, $CDCl_3$, 298 K, mixture of two isomers): δ = 165.4, 165.0, 139.6, 134.9, 131.5, 130.9, 129.5, 128.9, 128.7, 73.3, 70.0, 67.0, 49.6, 37.0, 32.3, 30.0, 28.9, 20.2.

HRMS-ESI calcd for $C_{11}H_{13}ClONa$ $[M+Na]^+$: 219.0553, found: 219.0547.

9.6. 2-(*p*-Fluorophenyl)-5-methyltetrahydrofuran (3f)

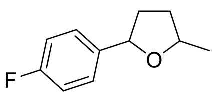

3f

The general procedure (d) was applied using 1-(4-fluorophenyl)pentane-1,4-diol (**2f**, 198 mg, 1 mmol). The product was purified by column chromatography using petroleum ether and ethyl acetate (9:1 v/v) as eluent. (128 mg, 0.71 mmol). The spectroscopic data was in accordance with the data reported in the literature.[8]

Yield: 71%, dr: 0.44:1

^{1}H NMR (400 MHz, $CDCl_3$, 298 K, mixture of two isomers): δ = 7.34-7.27 (m, 2 H, Ar *cis+trans* isomers), 7.04-6.98 (m, 2 H, Ar *cis+trans* isomers), 5.00 (dd, 1 H, *J* = 8.2, 6.4 Hz, H-5 *trans* isomer), 4.84 (t, 1 H, *J* = 7.3 Hz, H-5 *cis* isomer), 4.38-4.30 (m, 1 H, H-2 *trans* isomer), 4.19-4.09 (m, 1 H, H-2 *cis* isomer), 2.40-2.33 (m, 1 H, H-4 *trans* isomer), 2.32-2.24 (m, 1 H, H-4 *cis* isomer), 2.20-2.13 (m, 1 H, H-4 *trans* isomer), 2.12-2.05 (m, 1 H, H-4 *cis* isomer), 1.87-1.75 (m, 1 H, H-3 *cis+trans* isomers), 1.67-1.55 (m, 1 H, H-3 *cis+trans* isomers), 1.36 (d, 3 H, *J* = 6.1 Hz, CH_3 *cis* isomer), 1.31 (d, 3 H, *J* = 6.1 Hz, CH_3 *trans* isomer).

^{13}C NMR (100 MHz, $CDCl_3$, 298 K, mixture of two isomers): δ = 162.0 (d, $J(^{13}C, ^{19}F)$ = 244.6 Hz), 161.96 (d, $J(^{13}C, ^{19}F)$ = 244.6 Hz), 139.6 (d, $J(^{13}C, ^{19}F)$ = 3.0 Hz),

139.2 (d, $J(^{13}C, ^{19}F)$ = 3.0 Hz), 127.5 (d, $J(^{13}C, ^{19}F)$ = 8.0 Hz), 127.1 (d, $J(^{13}C, ^{19}F)$ = 8.0 Hz), 115.2 (d, $J(^{13}C, ^{19}F)$ = 21.3 Hz), 80.4, 79.7, 76.0, 75.9, 35.7, 34.7, 34.3, 33.0, 21.5, 21.4.

9.7. 2-(*m*-Fluorophenyl)-5-methyltetrahydrofuran (3g)

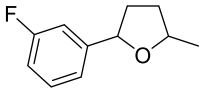
3g

The general procedure (d) was applied using 1-(3-fluorophenyl)pentane-1,4-diol (**2g**, 198 mg, 1 mmol). The product was purified by column chromatography using petroleum ether and ethyl acetate (20:1 v/v) as eluent. (90 mg, 0.50 mmol). The spectroscopic data was in accordance with the data reported in the literature.[3,8]

Yield: 50%, dr: 0.64:1

^{1}H NMR (400 MHz, $CDCl_3$, 298 K, mixture of two isomers): δ = 7.29-7.24 (m, 1 H, Ar *cis+trans* isomers), 7.11-7.04 (m, 2 H, Ar *cis+trans* isomers), 6.94-6.89 (m, 1 H, Ar *cis+trans* isomers), 5.04-5.01 (m, 1 H, H-2 *trans* isomer), 4.87 (t, 1 H, *J* = 7.31 Hz, H-2 *cis* isomer), 4.37-4.30 (m, 1 H, H-5 *trans* isomer), 4.19-4.13 (m, 1 H, H-5 *cis* isomer), 2.42-2.36 (m, 1 H, H-4 *trans* isomer), 2.32-2.27 (m, 1 H, H-4 *cis* isomer), 2.16-2.10 (m, 1 H, H-4 *trans* isomer), 2.09-2.04 (m, 1 H, H-4 *cis isomer*), 1.86-1.77 (m, 1 H, H-3 *cis+trans* isomers), 1.65-1.54 (m, 1 H, H-3 *cis+trans* isomers), 1.35 (d, 3 H, *J* = 6.1 Hz, CH_3 *cis* isomer), 1.31 (d, 3 H, *J* = 6.1 Hz, CH_3 *trans* isomer).

^{13}C NMR (100 MHz, $CDCl_3$, 298 K): δ = 164.1, 164.0, 162.1, 162.1, 147.1, 147.1, 146.6, 146.6, 129.8, 129.8, 121.4, 121.4, 121.2, 121.1, 114.0, 113.9, 113.9, 113.9, 113.8, 112.8, 112.7, 112.5, 112.4, 80.4, 80.4, 79.7, 76.3, 76.2, 35.7, 34.8, 34.8, 34.2, 33.0, 29.8, 21.5, 21.4.

9.8. 2-(*p*-Bromophenyl)-5-methyltetrahydrofuran (3h)

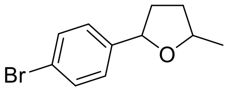
3h

The general procedure (d) was applied using 1-(4-bromophenyl)pentane-1,4-diol (**2h**, 259 mg, 1 mmol). The product was purified by column chromatography using petroleum ether and ethyl acetate (20:1 v/v) as eluent. (169 mg, 0.70 mmol).

Yield: 70%, dr: 0.52:1

^{1}H NMR (500 MHz, $CDCl_3$, 298 K, mixture of two isomers): δ = 7.46-7.42 (m, 2 H, Ar *cis+trans* isomers), 7.24-7.19 (m, 2 H, Ar *cis+trans* isomers), 5.00-4.97 (m, 1 H,

H-2 *trans* isomer), 4.83 (t, 1 H, *J* = 7.3 Hz, H-2 *cis* isomer), 4.36-4.30 (m, 1 H, H-5 *trans* isomer), 4.19-4.13 (m, 1 H, H-5 *cis* isomer), 2.41-2.35 (m, 1 H, H-4 *trans* isomer), 2.33-2.26 (m, 1 H, H-4 *cis* isomer), 2.17-2.12 (m, 1 H, H-4 *trans* isomer), 2.12-2.05 (m, 1 H, H-4 *cis isomer*), 1.84-1.74 (m, 1 H, H-3 *cis+trans* isomers), 1.65-1.54 (m, 1 H, H-3 *cis+trans* isomers), 1.36 (d, 3 H, *J* = 6.1 Hz, CH_3 *cis* isomer), 1.31 (d, 3 H, *J* = 6.1 Hz, CH_3 *trans* isomer).

^{13}C NMR (125 MHz, $CDCl_3$, 298 K): δ = 143.3, 142.8, 131.5, 131.4, 127.7, 127.7, 127.7, 127.4, 120.9, 120.8, 80.4, 79.7, 76.2, 76.2, 35.7, 34.8, 34.3, 33.1, 33.1, 33.1, 21.6, 21.4.

HRMS-ESI calcd for $C_{11}H_{13}BrOK$ $[M+K]^+$: 278.9787, found: 278.9794.

9.9. 2-(2-naphthalenyl)-5-methyltetrahydrofuran (3i)

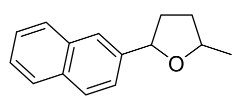

3i

The general procedure (d) was applied using 1-(2-naphtyl)-pentane-1,4-diol (**2i**, 230 mg, 1 mmol). The product was purified by column chromatography using petroleum ether and ethyl acetate (20:1 v/v) as eluent. (170 mg, 0.80 mmol). The spectroscopic data was in accordance with the data reported in the literature.[8]

Yield: 80%, dr: 0.44:1

^{1}H NMR (400 MHz, $CDCl_3$, 298 K, mixture of two isomers): δ = 7.86-7.81 (m, 2 H, Ar *cis+trans* isomers), 7.51-7.43 (m, 2 H, Ar *cis+trans* isomers), 5.25-5.21 (m, 1 H, H-2 *trans* isomer), 5.07 (t, 1 H, *J* = 7.3 Hz, H-2 *cis* isomer), 4.49-4.41 (m, 1 H, H-5 *trans* isomer), 4.29-4.21 (m, 1 H, H-5 *cis* isomer), 2.50-2.43 (m, 1 H, H-4 *trans* isomer), 2.43-2.34 (m, 1 H, H-4 *cis* isomer), 2.24-2.19 (m, 1 H, H-4 *trans* isomer), 2.18-2.10 (m, 1 H, H-4 *cis isomer*), 2.01-1.89 (m, 1 H, H-3 *cis+trans* isomers), 1.73-1.62 (m, 1 H, H-3 *cis+trans* isomers), 1.45 (d, 3 H, *J* = 6.1 Hz, CH_3 *cis* isomer), 1.38 (d, 3 H, *J* = 6.1 Hz, CH_3 *trans* isomer).

^{13}C NMR (100 MHz, $CDCl_3$, 298 K, mixture of two isomers): δ = 141.4, 141.0, 133.4, 132.9, 132.8, 128.1, 127.9, 127.7, 126.0, 125.6, 124.3, 124.2, 124.0, 123.9, 81.8, 80.4, 40.9, 35.6, 34.6, 34.3, 33.2, 23.9, 21.6, 21.4, 20.8, 17.5, 17.3, 14.7.

9.10. 2,5-Diphenyltetrahydrofuran (3j)

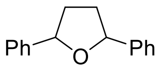

3j

The general procedure (d) was applied using 1,4-diphenylbutane-1,4-diol (**2j**, 242 mg, 1 mmol). The product was purified by column chromatography as a mixture of diastereoisomers using petroleum ether and ethyl acetate (20:1 v/v) (174 mg, 0.78 mmol). The spectroscopic data was in accordance with the data reported in the literature.[11]

Yield: 78%, dr: 0.55:1

^{1}H NMR (400 MHz, $CDCl_3$, 298 K, mixture of two isomers): δ = 7.49-7.29 (m, 10 H, Ar *cis+trans* isomers), 5.30-5.28 (m, 1 H, C*H*Ar, *trans* isomer), 5.09-5.07 (m, 1 H, C*H*Ar, *cis* isomer), 2.53-2.45 (m, 2 H, CH_2 *cis+trans* isomers), 2.06-1.97 (m, 2 H, CH_2 *cis+trans* isomers).

^{13}C NMR (100 MHz, $CDCl_3$, 298 K): δ = 143.7, 143.0, 128.4, 128.4, 127.3, 127.2, 126.0, 125.6, 81.4, 81.3, 35.6, 34.4.

9.11. 2,5-Dimethyltetrahydrofuran (3k)

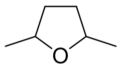

3k

The general procedure (d) was applied using 2,3-hexanediol (**2k**, 118 mg, 1 mmol). The product yield was obtained by ^{1}H NMR spectroscopy using 1,3,5-trimethoxybenzene as an internal standard. The spectroscopic data was in accordance with the data reported in the literature.[12]

Yield: 70%, dr: 0.69:1

Selected ^{1}H NMR signals (400 MHz, $CDCl_3$, 298 K, mixture of two isomers): δ = 4.37 (m, 1 H, C*H*CH_3, *trans* isomer), 4.16 (m, 1 H, C*H* CH_3, *cis* isomer).

9.12. 2-Phenyltetrahydrofuran (3l)

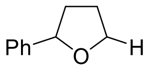

3l

The general procedure (d) was applied using 1-phenyl-1,4-butanediol (**2l**, 166 mg, 1 mmol). The product yield was obtained by ^{1}H NMR spectroscopy using 1,3,5-trimethoxybenzene as an internal standard. The spectroscopic data was in accordance with the data reported in the literature.[13]

Yield: 24%

Selected ^{1}H NMR signals (400 MHz, $CDCl_3$, 298 K): δ = 4.83 (dd, 1 H, *J* = 14.4, *J* = 7.2 Hz, H-2), 4.12 (dd, 1 H, *J* = 14.4, 7.2 Hz, H-5).

9.13. 2-Methyl-5-phenyl-methyltetrahydrofuran-2,5-*d*$_2$ (3a-*d*$_2$)

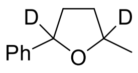

3a-*d*$_2$

The general procedure was applied using 1-phenylpentane-1,4-*d*$_2$-1,4-diol (**2a-*d*$_2$** 182 mg, 1 mmol) with 24 h reaction time. Product **3a-*d*$_2$** was purified by column chromatography using petroleum ether and ethyl acetate (9:1 v/v) as eluent. (133 mg, 0.81 mmol).

Yield: 81% yield, dr: 1:1

^{1}H NMR (400 MHz, $CDCl_3$, 298 K, mixture of two isomers): δ = 7.36-7.26 (m, 5 H, Ar *cis+trans* isomers), 1.92-1.80 (m, 2 H, H-4 *cis+trans* isomers), 1.66-1.44 (m, 2 H, H-3 *cis+trans* isomers), 1.19 (s, 3 H, C*H*$_3$ *cis+trans* isomers).

^{13}C NMR (100 MHz, $CDCl_3$, 298 K, mixture of two isomers): δ = 144.8, 144.7, 128.5, 128.4, 127.6, 125.8, 125.7, 74.4 (t, *J*(13C, 2H) = 21.6 Hz), 74.0 (t, *J*(13C, 2H) = 22.1 Hz), 67.9 (t, *J*(13C, 2H) = 21.9 Hz), 67.5 (t, *J*(13C, 2H) = 21.9 Hz), 35.9, 35.8, 35.0, 23.7, 23.5.

HRMS-ESI calcd for $C_{12}H_{16}D_2O_3Na$ $[M+Na]^+$: 187.2319, found: 187.2025.

9.14. 2-(*p*-Methoxyphenyl)-5-methyltetrahydrofuran-2,5-*d*$_2$ (3b-*d*$_2$)

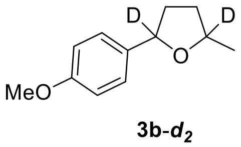

3b-*d*$_2$

The general procedure was applied using 1-(*p*-methoxyphenyl)pentane-1,4-*d*$_2$-1,4-diol (**2b-*d*$_2$**, 212 mg, 1 mmol) with 24 h reaction time. Product **3b-*d*$_2$** was purified by column chromatography using petroleum ether and ethyl acetate (9:1 v/v) as eluent. (166 mg, 0.85 mmol).

Yield: 85%, dr: 0.76:1

^{1}H NMR (400 MHz, $CDCl_3$, 298 K, mixture of two isomers): δ = 7.30-7.24 (m, 2 H, Ar *cis+trans* isomers), 6.89-6.85 (m, 2 H, Ar *cis+trans* isomers), 3.79 (s, 3 H, OC*H*$_3$ *cis+trans* isomers), 2.35-2.29 (m, 1 H, H-4 *trans* isomer), 2.27-2.21 (m, 1 H, H-4 *cis* isomer), 2.18-2.12 (m, 1 H, H-4 *trans* isomer), 2.11-2.04 (m, 1 H, H-4 *cis* isomer), 1.89-1.78 (m, 1 H, H-3 *cis+trans* isomers), 1.64-1.56 (m, 1 H, H-3 *cis+trans* isomers), 1.34 (s, 3 H, C*H*$_3$ *cis* isomer), 1.30 (s, 3 H, C*H*$_3$ *trans* isomer).

^{13}C NMR (100 MHz, $CDCl_3$, 298 K, mixture of two isomers): δ = 135.8, 135.4, 127.07 (d, *J*(13C, 2H) = 2.6 Hz), 113.7, 55.3, 35.4, 34.4, 34.2, 23.55 (d, *J* = 23.6 Hz), 21.5, 21.3.

HRMS-ESI calcd for $C_{12}H_{16}D_2O_3Na$ $[M+Na]^+$: 217.2554, found: 217.2540.

9.15. 2-Phenyl-6-methyltetrahydropyran (3m)

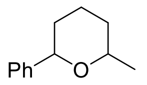

3m

The general procedure (d) was applied using 1-phenylhexane-1,5-diol (**2n**, 194 mg, 1 mmol). After an additional hydrogenation step with Pd/C (10 mol%) under 1 bar of H_2 overnight, and a column chromatography using petroleum ether and ethyl acetate (20:1 v/v) as eluent, the desired tetrahydropyran **3m** was obtained (60 mg, 0.34 mmol). The spectroscopic data was in accordance with the data reported in the literature.[14,15]

Yield: 34%, dr: 0.32:1

^{1}H NMR (400 MHz, $CDCl_3$, 298 K, mixture of two isomers): δ = 7.41-7.22 (m, 5 H, Ar *cis+trans* isomers), 4.87 (t, 1 H, *J* = 5.4 Hz, H-6 *trans* isomer), 4.37 (dd, 1 H, *J* = 11.2 Hz, *J* = 2.2 Hz, H-6 *cis* isomer), 4.00-3.93 (m, 1 H, H-2 *trans* isomer), 3.68-3.60 (m, 1 H, H-2 *cis* isomer), 1.94-1.90 (m, 1 H, H-5 *cis+trans* isomers), 1.82-1.78 (m, 1 H, H-5 *cis+trans* isomers), 1.72-1.62 (m, 2 H, H-4 *cis+trans* isomers), 1.52-1.29 (m, 2 H, H-3 *cis+trans* isomers), 1.28-1.25 (m, 3 H, C*H$_3$* *cis+trans* isomers).

^{13}C NMR (100 MHz, $CDCl_3$, 298 K, mixture of two isomers): δ = 143.5, 142.4, 128.3, 128.2, 127.2, 126.9, 126.4, 125.9, 79.9, 74.4, 72.3, 67.9, 33.5, 33.1, 31.4, 30.3, 24.2, 22.3, 19.4, 18.8.

9.16. 2,6-Diphenyl-tetrahydropyran (3n), 2,6-diphenyl-2,3-dihydropyran (3n')

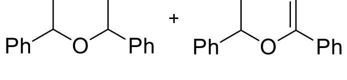

3n **3n'**

The general procedure (d) was applied using 1,5-diphenylpentane-1,5-diol (**2n**, 256 mg, 1 mmol). After an additional hydrogenation step with Pd/C (10 mol%) under 1 bar of H_2 overnight, and a column chromatography using petroleum ether and ethyl acetate (20:1 v/v) as eluent, a non-separable mixture of the 2,6-diphenyl-tetrahydropyran (**3n**) and 2,6-diphenyl-2,3-dihydropyran (**3n'**) (0.9 : 1 ratio) was obtained (98 mg, 0.41 mmol). The spectroscopic data was in accordance with the data reported in the literature.[16]

Yield: 19% (**3n**, dr: 0.15:1), 22% (**3n’**)

Selected NMR signals of compound **3n**:

^{1}H NMR (400 MHz, $CDCl_3$, 298 K, mixture of two isomers): δ = 4.88 (dd, *J* = 6.5, 4.2 Hz, C*H*Ar *cis* isomer), 4.59 (dd, 2 H, *J* = 11.4, 2.0 Hz, C*H*Ar *trans* isomer).

Selected NMR signals of compound **3n’**:

^{1}H NMR (400 MHz, $CDCl_3$, 298 K): δ = 7.65-7.63 (m, 2 H, *o*-Ph-C6), 5.6 (ddd, 1 H, *J* = 5.2, 3.0, 1.0 Hz, C2=C*H*), 5.04 (dd, 1 H, *J* = 10.1, 2.3 Hz, C*H*Ar).

10. Identification of oxidized linear products

The general procedure was applied using 1-phenyl-1,4-pentanediol (180 mg, 1 mmol) and **1c** as the catalyst.

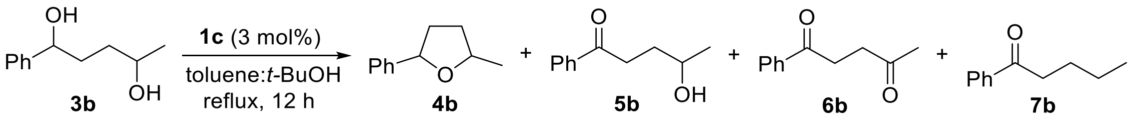

Scheme S5. Cyclodehydration of diol **2a** catalyzed by **1c**.

Selected NMR signals of compound **5b**:[17]

^{1}H NMR (400 MHz, $CDCl_3$, 298 K): δ = 3.17 (m, 2 H), 1.26 (d, 3 H, *J* = 6.2 Hz).

Selected NMR signals of compound **6b**:[18]

^{1}H NMR (400 MHz, $CDCl_3$, 298 K): δ = 3.28 (t, 2 H, *J* = 6.4 Hz), 2.89 (t, 2 H, *J* = 6.3 Hz), 2.26 (s, 3 H).

Selected NMR signals of compound **7b**:[19]

^{1}H NMR (400 MHz, $CDCl_3$, 298 K): δ = 2.97 (t, 2 H, *J* = 7.3 Hz), 1.73 (m, 2 H), 1.42 (m, 2 H), 0.96 (t, 3 H, *J* = 7.3 Hz).

11. Study of the transient products in the cyclodehydration of diols

When the reactions were run at lower temperatures, the amount of these transient intermediates in the crude reaction mixtures increased. Importantly, upon further heating at higher temperature, they were transformed into the desired final cyclic ethers.

Table S2. Effect of the temperature in the cyclodehydration of **2j**.[a]

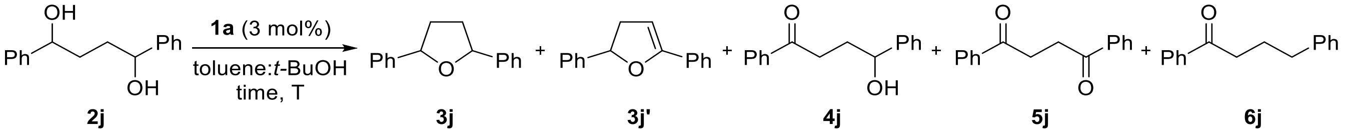

entry	t (h)	T (°C)	**2j** (%)[b]	**3j** (%)[b]	**3j'** (%)[b]	**4j** (%)[b]	**5j** (%)[b]	**6j** (%)[b]
1	4	80	76	6	<1	17	<1	<1
2	24	80	39	37	<1	2	2	<1
3	4	130	<1	59	17	17	7	<1
4	24	130	<1	78	14	<1	2	5

[a] Reaction conditions: **2j** (0.5 mmol), **1a** (0.015 mmol, 3 mol%), toluene (1.3 mL), *t*-BuOH (0.5 mL), 80-130 °C, 4-24 h. [b] Yields determined by ^{1}H NMR spectroscopy.

12. Oxidative process of unsaturated 1,4-diols

The unsaturated derivatives of 1,4-diol **12a**, i.e. ***E*-12a** and ***Z*-12a**,[20] did not undergo cyclization processes. Instead, double oxidation and elimination products were observed in the crude NMR spectra.

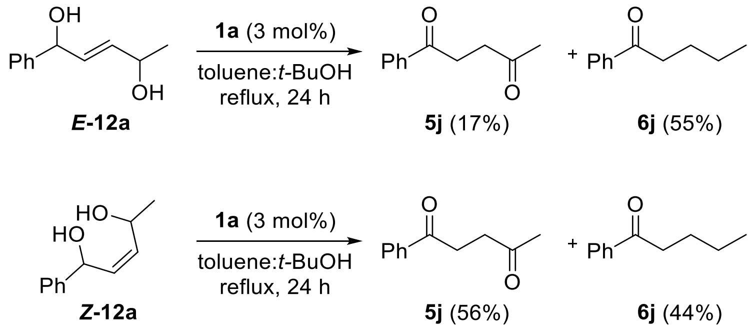

Scheme S6. Oxidative process of unsaturated diols catalyzed by **1a**.

13. Mechanistic investigations

13.1. Preparation of the stock solution of catalyst

To a microwave vial containing $AgBF_4$ (0.0945 mmol, 18.4 mg), iridium complex **1a** (0.045 mmol, 27.5 mg) and anhydrous and degassed CH_2Cl_2 (4 mL) were added. The reaction mixture was stirred for 2 h at room temperature. The mixture was filtered-off through a pad of cotton to remove the AgCl precipitate and distributed to 20 NMR tubes. The solvent was evaporated under vacuum, and the NMR tubes could then be stored under inert atmosphere. The activity of the catalyst is slightly different from solution to solution, thus for each mechanistic study the same batch of catalyst stock solution was used.

13.2. NMR scale mechanistic experiments procedure

An NMR tube impregnated with **1a** was loaded with *tert*-butanol (0.05 mL) and a stock solution of 1,4-diol in toluene-d_8 (0.2 mL, 0,075 mmol). The total reaction volume was adjusted to 500 µL by addition of toluene-d_8. The tube was introduced in an NMR spectrometer preheated at 100 °C.

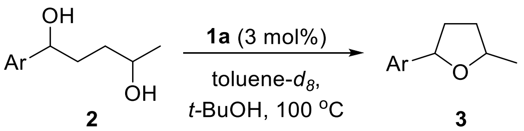

2 3

Scheme S7. Cyclodehydration of diols catalyzed by **1a** in NMR scale.

13.3. Hammett studies

Six parallel reactions were carried out with 1,4-diols **2a**–**2f** following the general procedure for mechanistic studies. The tube was transferred to the NMR spectrometer with the probe preheated at 100 ºC. ^{1}H NMR spectra were recorded every 2 min. Each experiment was done thrice. Changes in the ratio between the integration of the signals from the protons in the carbon atoms in alpha position to the alcohol moieties in substrates **2a**–**2f** and from the protons in positions C2 and C5 in the tetrahydrofuran products **3a**–**3f** were used to monitor the consumption of substrate. Each experiment was done three times. The results are presented in Figure S1.

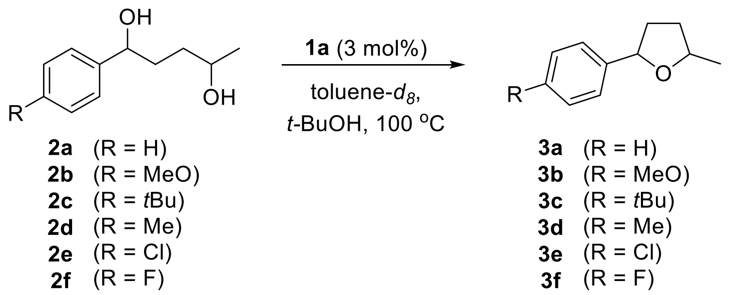

Scheme S8. Non-competition Hammett studies using diols **2a**–**2f**.

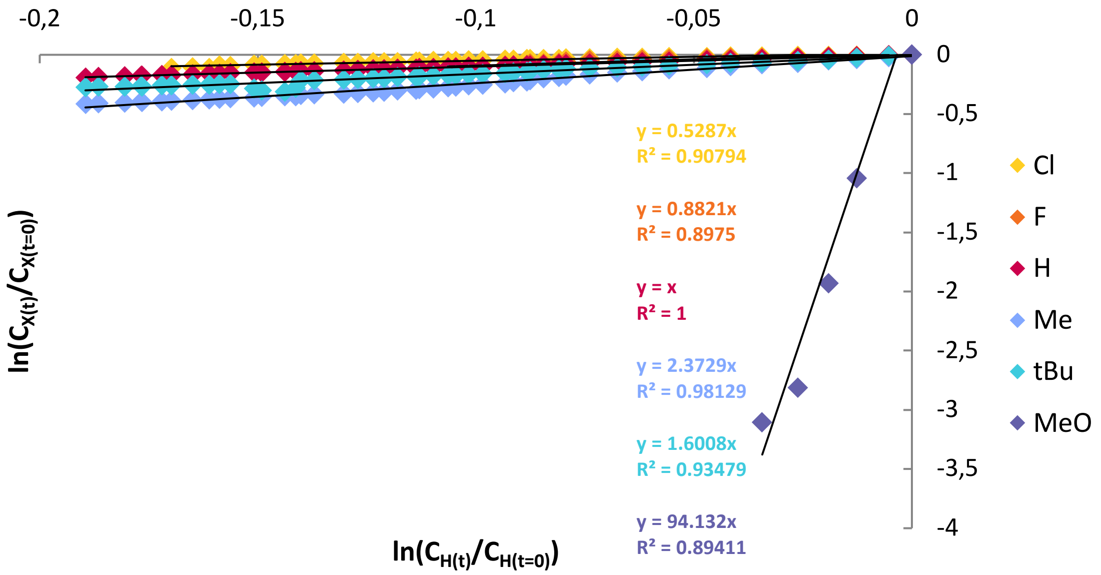

Figure S1. Average of non-competitive parallel reaction using diols **2a**–**2f**.

13.4. Kinetic isotope effect (KIE)

Deuterated 1,4-diols were synthesized following the general procedure for the reduction of diketones to diols (Section S7, *vide supra*) using $NaBD_4$.

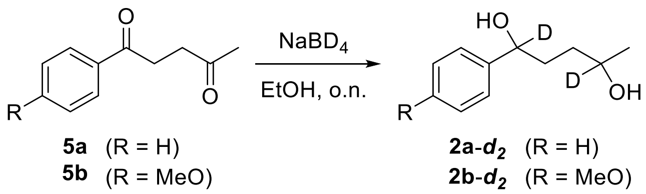

Scheme S9. Synthesis of deuterated 1,4-diols.

Four parallel reactions were carried out with 1,4-diols **2a**, **2b**, **2a-*d*₂** and **2b-*d*₂**, following the general procedure for mechanistic studies with variations. The reaction solvent was toluene for substrates **2a** and **2b**, while toluene-d_8 was used for deuterated

substrates **2a**-***d*₂** and **2b**-***d*₂**. A stock solution (200 μL) containing the appropriate diol (0.375 mmol) in toluene, and *tert*-butanol (100 μL) were loaded to an NMR tube impregnated with catalyst **1a**. The total reaction volume was adjusted to 400 μL by addition of toluene or toluene-d_8. The tube was transferred to the NMR spectrometer with the probe preheated at 100 ºC. ^{1}H NMR spectra were recorded every 2 min. Changes in the ratio between the integration of the signals from the terminal methyl groups of diols **2** and tetrahydrofurans **3** were used to monitor the consumption of substrate. Each experiment was done thrice.

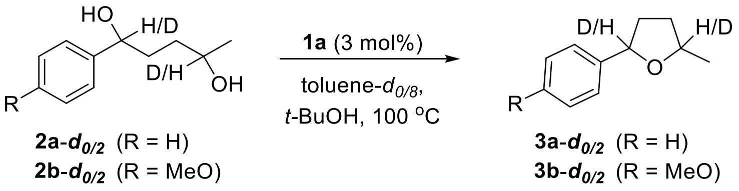

Scheme S10. Cyclodehydration of diols **2a**, **2b**, **2a**-$\boldsymbol{d_2}$ and **2b**-$\boldsymbol{d_2}$.

The initial rate plots for the experiments with alcohols **2a**-$\boldsymbol{d_0}$, **2a**-$\boldsymbol{d_2}$ are given in Figure S2. The measurement of the kinetic isotope effect for the cyclodehydration of diols **2a**-$\boldsymbol{d_{0/2}}$ resulted in a KIE value of 2.94 ± 0.14.

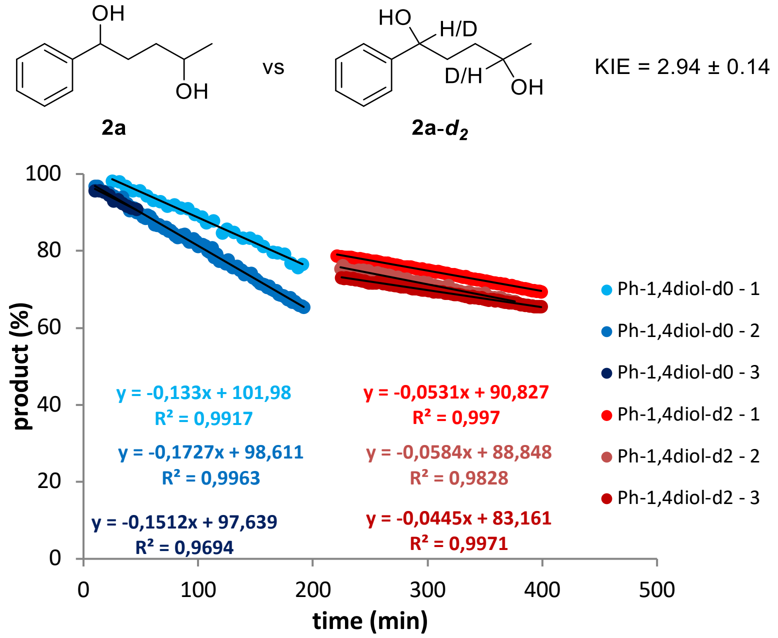

Figure S2. Kinetic Isotope Effect for diols **2a**-$\boldsymbol{d_{0/2}}$.

The initial rate plots for the experiments with alcohols **2b**, **2b-*d*$_2$** are given in Figure S3. The measurement of the kinetic isotope effect for the cyclodehydration of diols **2a-*d*$_{0/2}$** resulted in a KIE value of 1.14 ± 0.08.

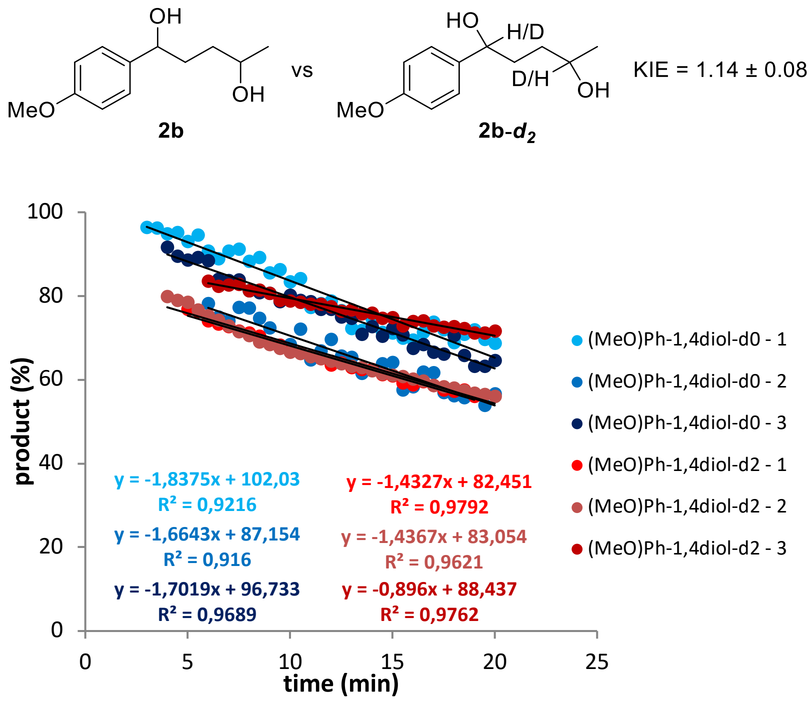

Figure S3. Kinetic Isotope Effect for diols **2a-*d*$_{0/2}$**.

14. Carbocation trapping experiments with nucleophiles

Our first attempt to trap the envisioned intermediate carbocation within the acid mediated mechanism did not afford any clear conclusion. The addition of a nucleophile, i.e. methanol, indole, pyrrole or *N*,*N*-dimethylaniline, to the electron-rich 1,4-diol **2b** under the general procedure (d) did only yield the corresponding tetrahydrofuran **3b** (Figure S4–Figure S7).[21] In contrast, methanol and indole succeeded in trapping the positively charged intermediate of the oxidative process of 1-(*p*-methoxyphenyl)-1-pentanol (**13b**) yielding the corresponding product **14b** as the major product of the reaction (Figure S8 and Figure S9), being further confirmed by LMRS (Figure S10).

The disparity in products obtained starting from **2b** and **13b** could appointed to the quick cyclization (See step *ii*, Scheme 3b) that the former undergoes and the latter, lacking the second alcohol moiety, does not. A reference experiment using 1-phenyl-1-pentanol (**13a**) only gave the oxidized product **6a** (Figure S11), confirming the difference in the reaction mechanism followed by very electron rich substrates, or mild electron rich and electron poor alcohols.

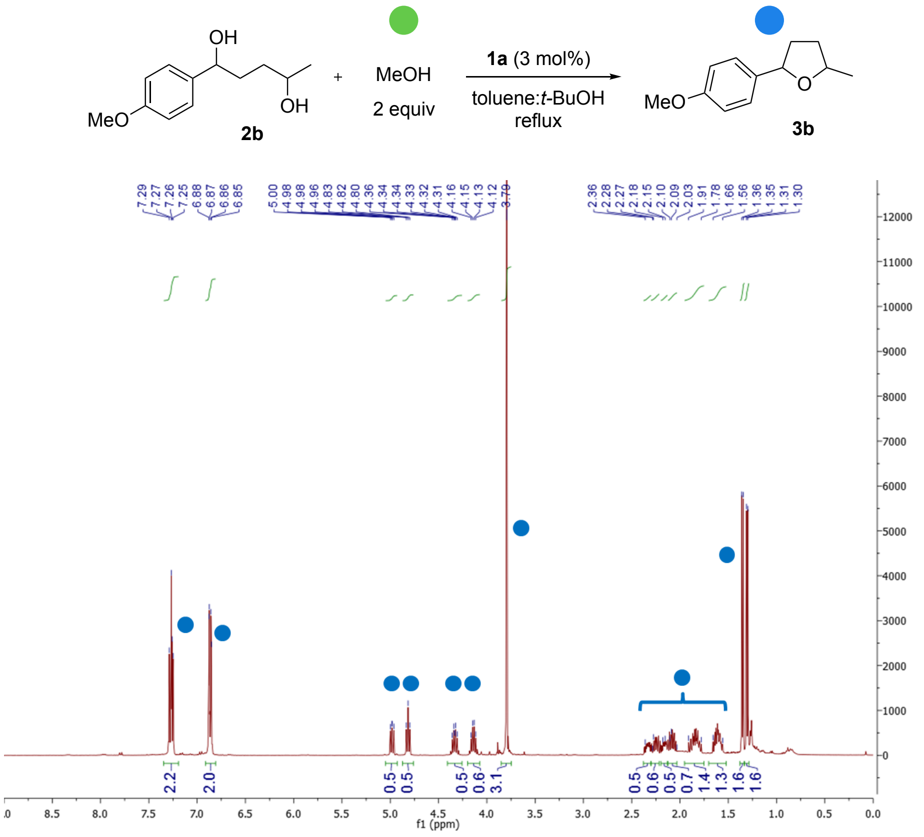

Figure S4. Crude ^{1}H NMR spectrum of reaction of **2b** with methanol as external nucleophile after 24 h reaction time. Methanol was removed *in vacuo*.

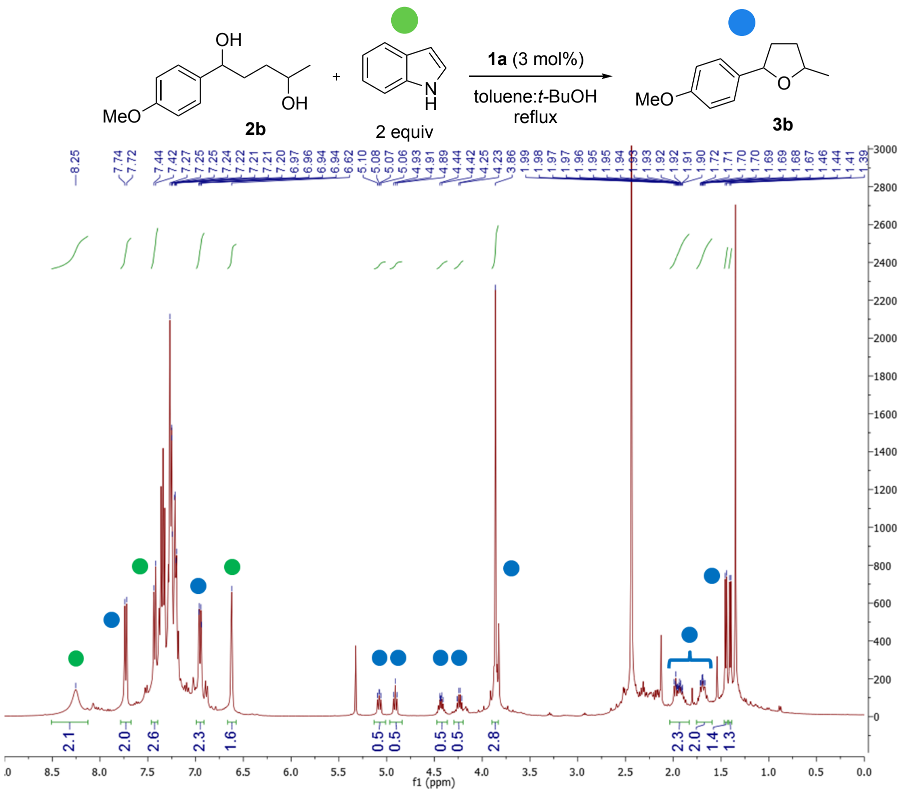

Figure S5. Crude ^{1}H NMR spectrum of reaction of **2b** with indole as external nucleophile after 24 h reaction time.

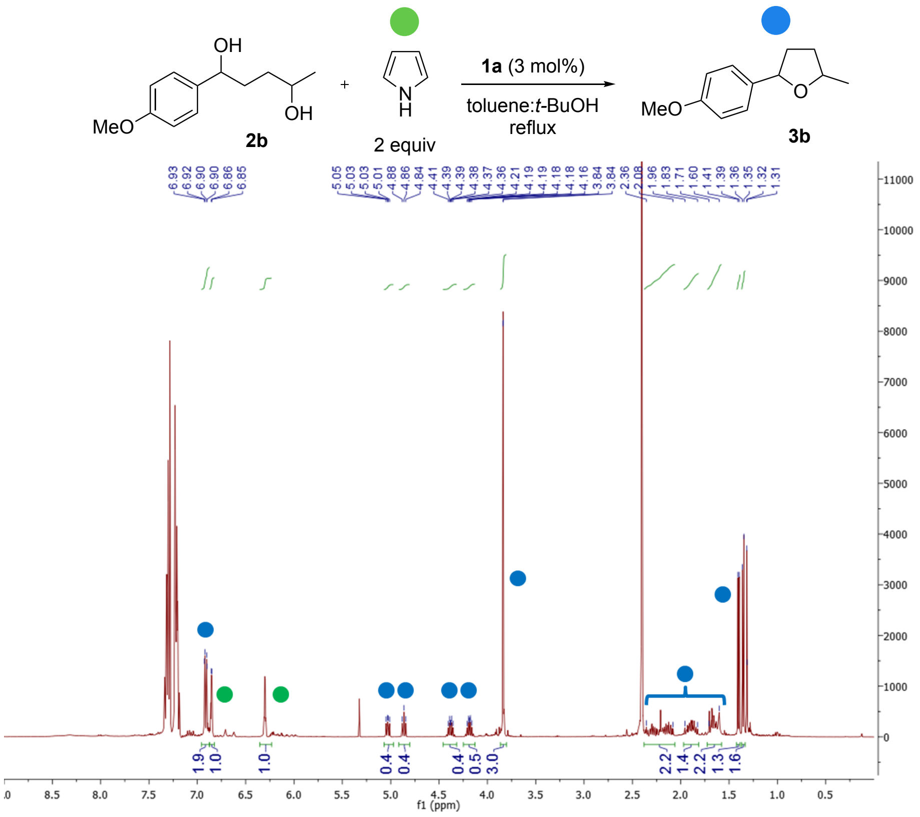

Figure S6. Crude ^{1}H NMR spectrum of reaction of **2b** with pyrrole as external nucleophile after 24 h reaction time.

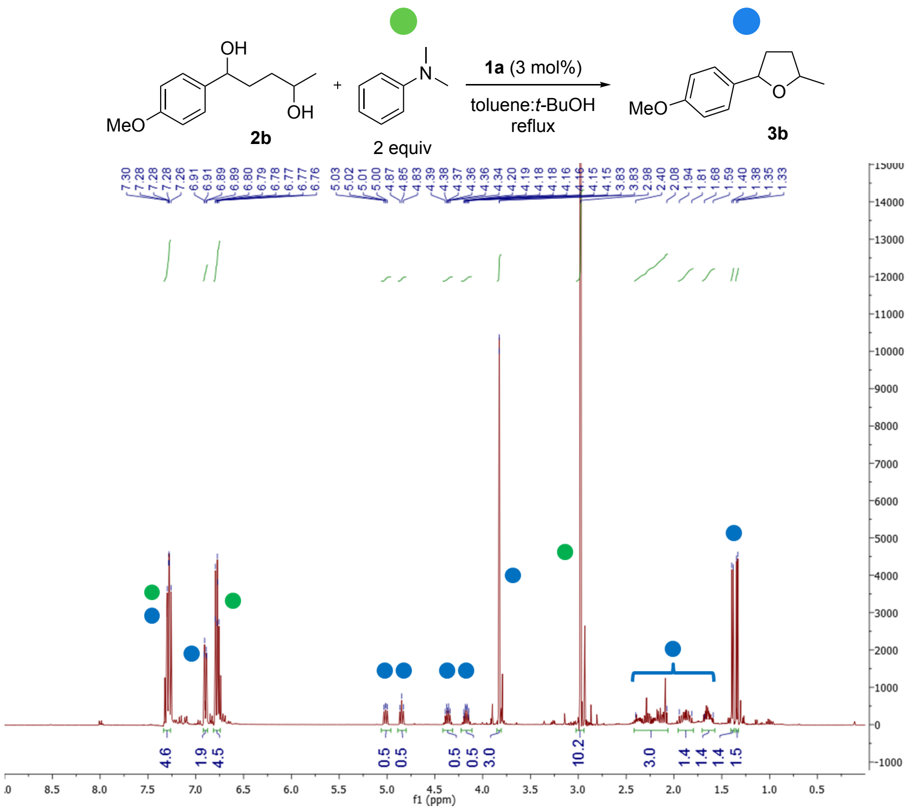

Figure S7. Crude ^{1}H NMR spectrum of reaction of **2b** with *N*,*N*-dimethylaniline as external nucleophile after 24 h reaction time.

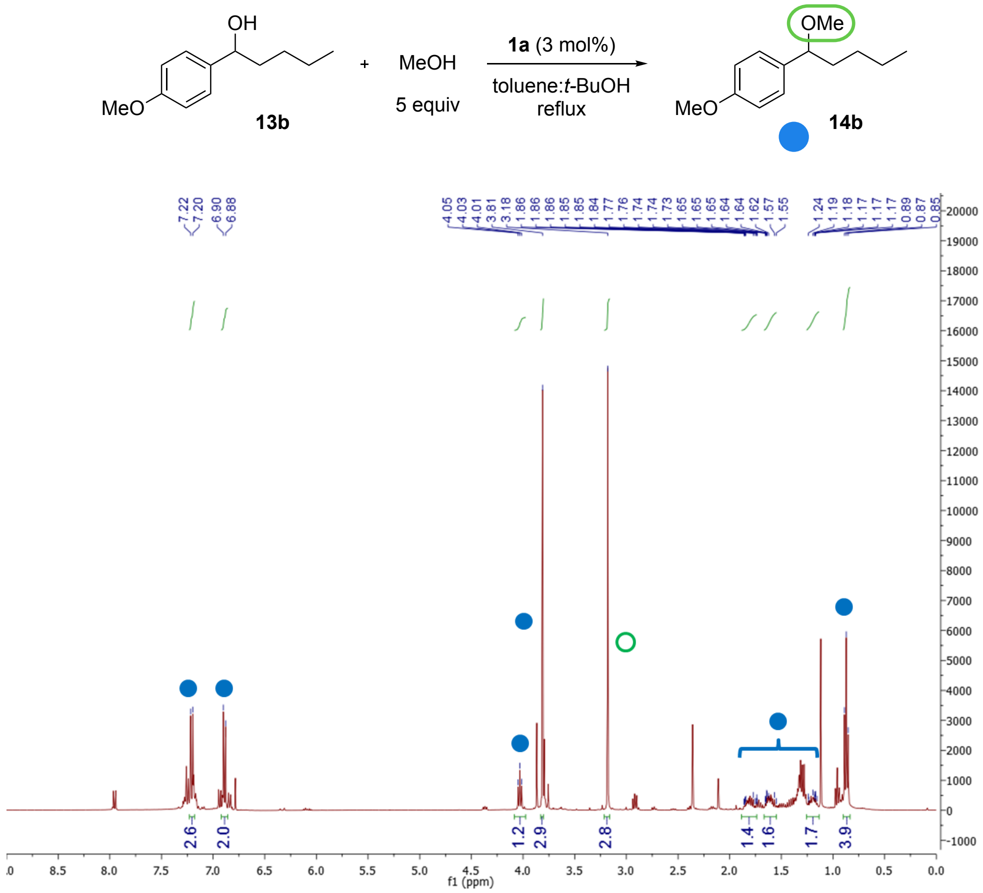

Figure S8. Crude ^{1}H NMR spectrum of product **14b** from the reaction of **13b** with methanol as external nucleophile after 24 h reaction time.

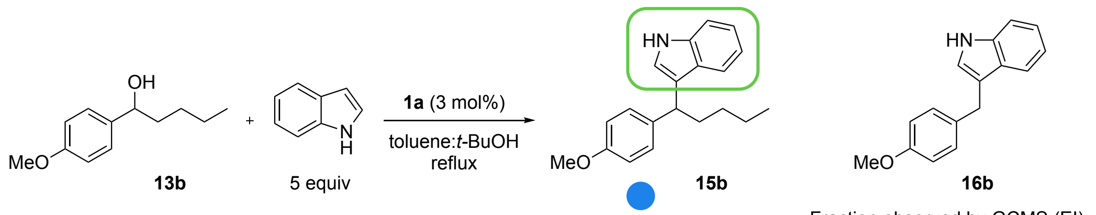

Fraction observed by GCMS (EI):
Calculated: 236.11, found: 236

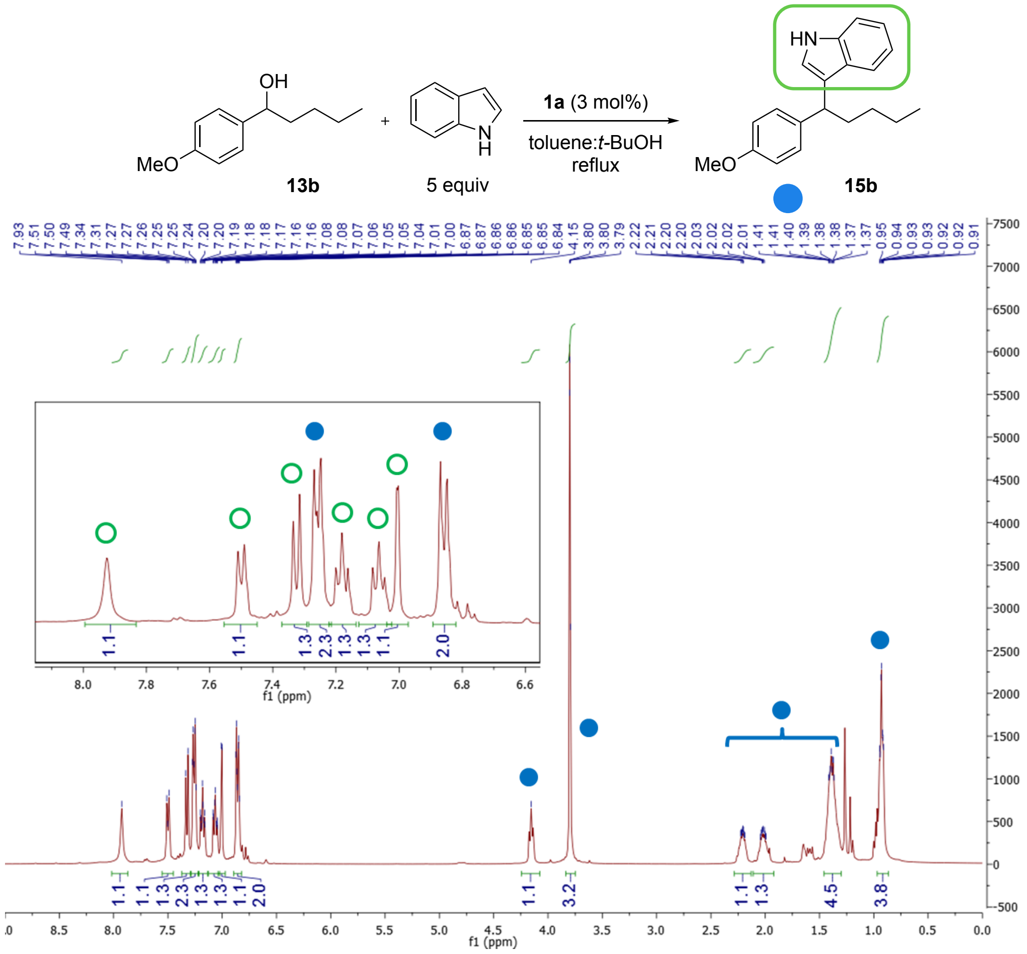

Figure S9. Pure ^{1}H NMR spectrum of product **15b** from the reaction of **13b** with indole as external nucleophile after 24 h reaction time. Excess of indole previously removed by column chromatography.

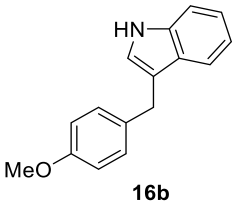

16b

Fraction observed by GCMS (EI):
Calculated: 236.11, found: 236

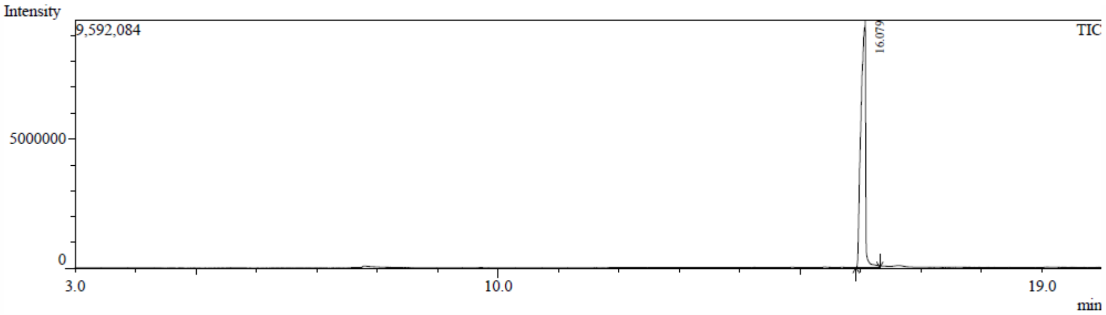

Peak Report TIC

Peak#	R.Time	I.Time	F.Time	Area	Area%	Height	Height%	A/H	Mark	Name
1	16.079	15.928	16.320	51513735	100.00	9558375	100.00	5.39	MI	
				51513735	100.00	9558375	100.00			

Spectrum

Peak#:1 R.Time:16.079(Scan#:7848)
MassPeaks:257
RawMode:Averaged 16.077-16.080(7847-7849)
BG Mode:Calc. from Peak Group 1 - Event 1 Scan

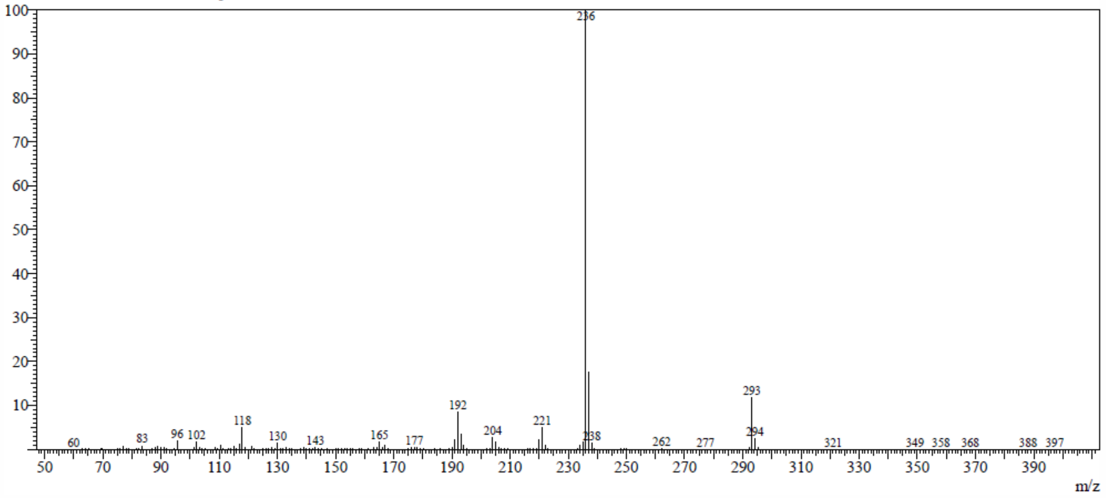

Figure S10. LRMS spectrum of **16b**.

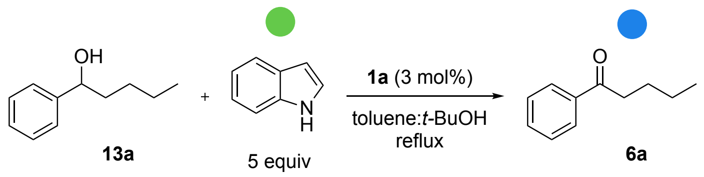

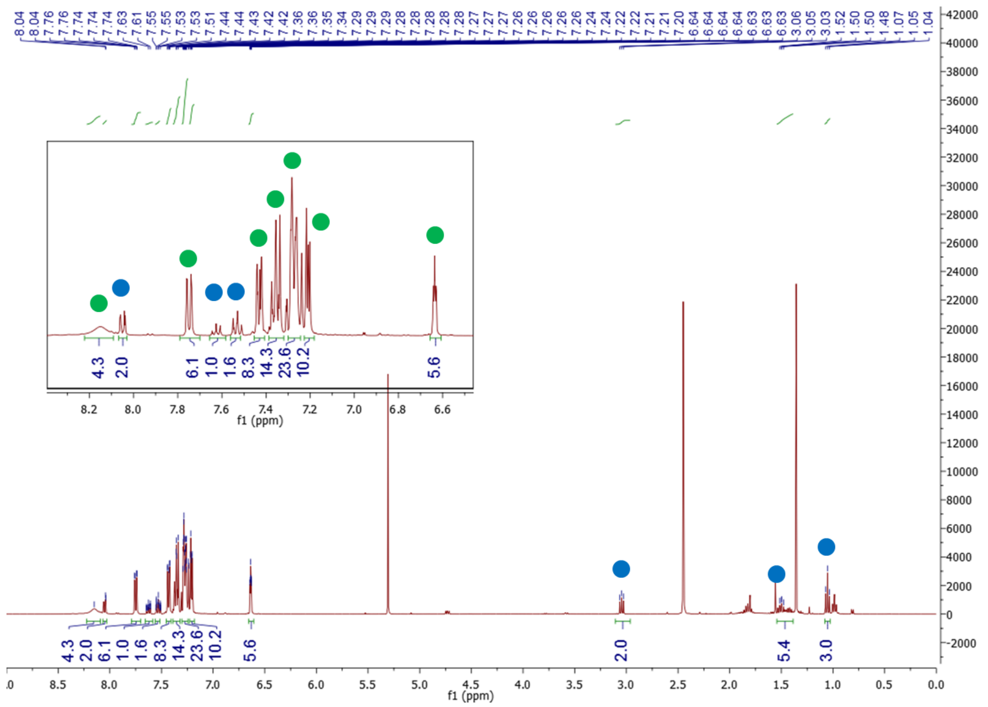

Figure S11. Crude ^{1}H NMR spectrum of product **6a** from the reaction of **13a** with indole as external nucleophile after 24 h reaction time.

15. NMR spectra of synthesized substrates and products

15.1. 1-Phenylpentane-1,4-diol (2a)

^{1}H NMR (400 MHz, $CDCl_3$, 298 K)

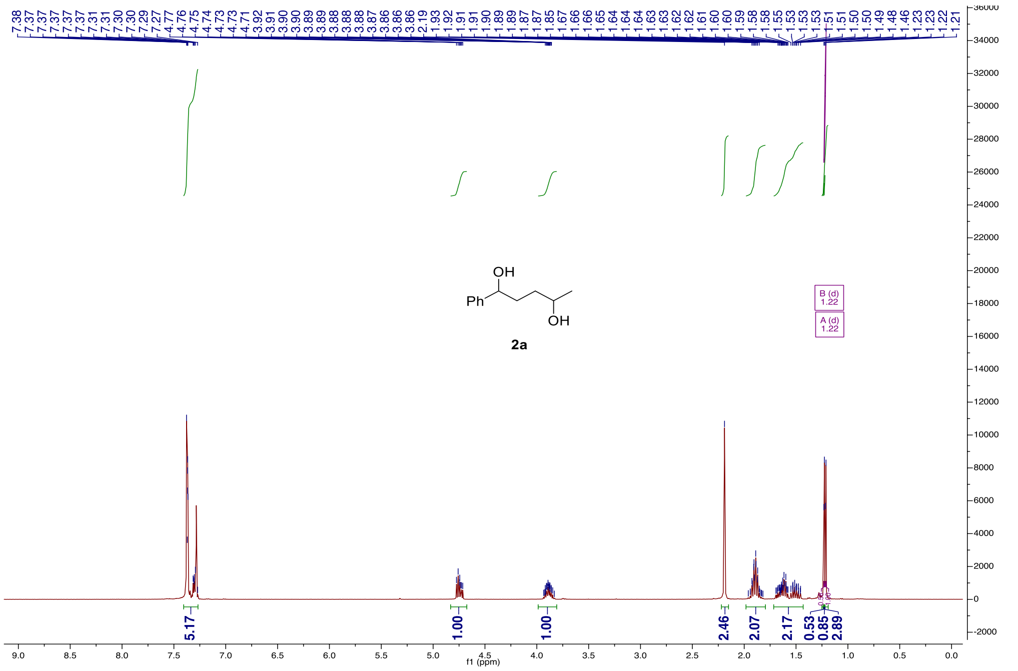

15.2. 1-(*p*-Methoxyphelyl)pentane-1,4-diol (2b)

^{1}H NMR (400 MHz, $CDCl_3$, 298 K)

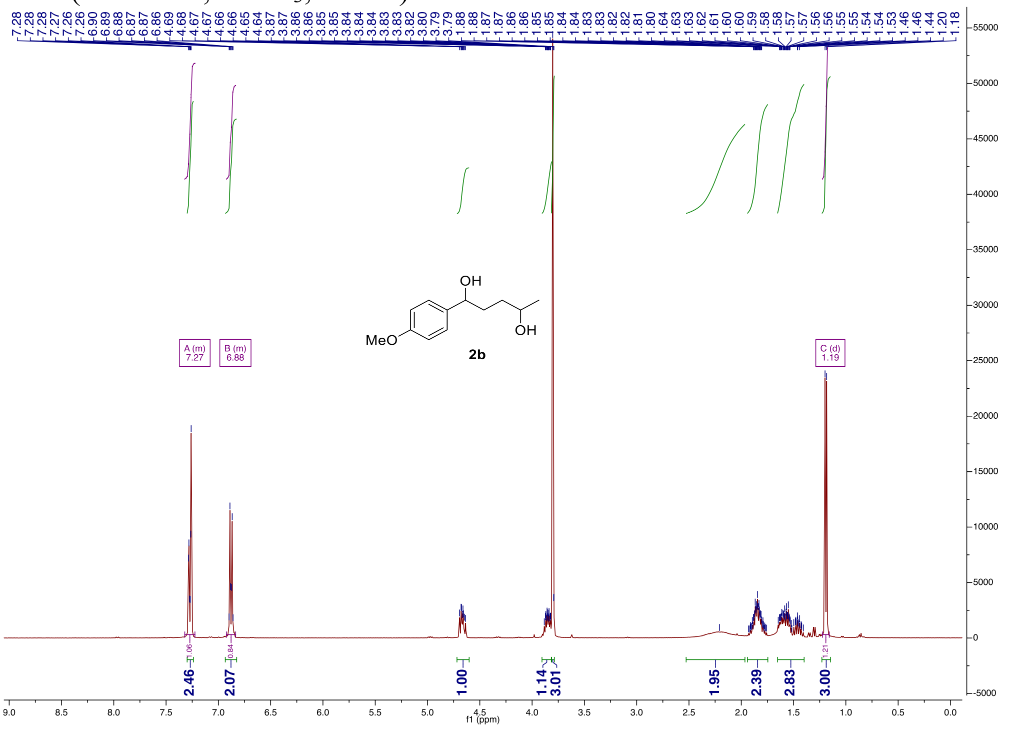

$^{13}C\{^{1}H\}$ NMR (100 MHz, $CDCl_3$, 298 K)

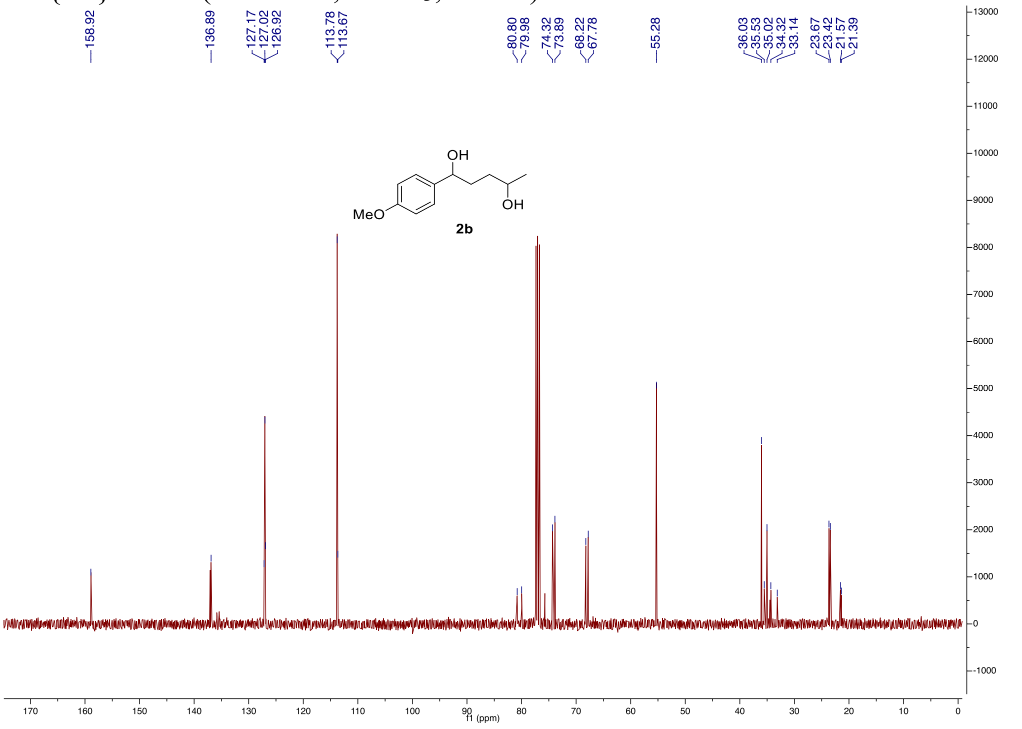

15.3. 1-(*p*-*tert*-Butylphenyl)pentane-1,4-diol (2c)

^{1}H NMR (400 MHz, $CDCl_3$, 298 K)

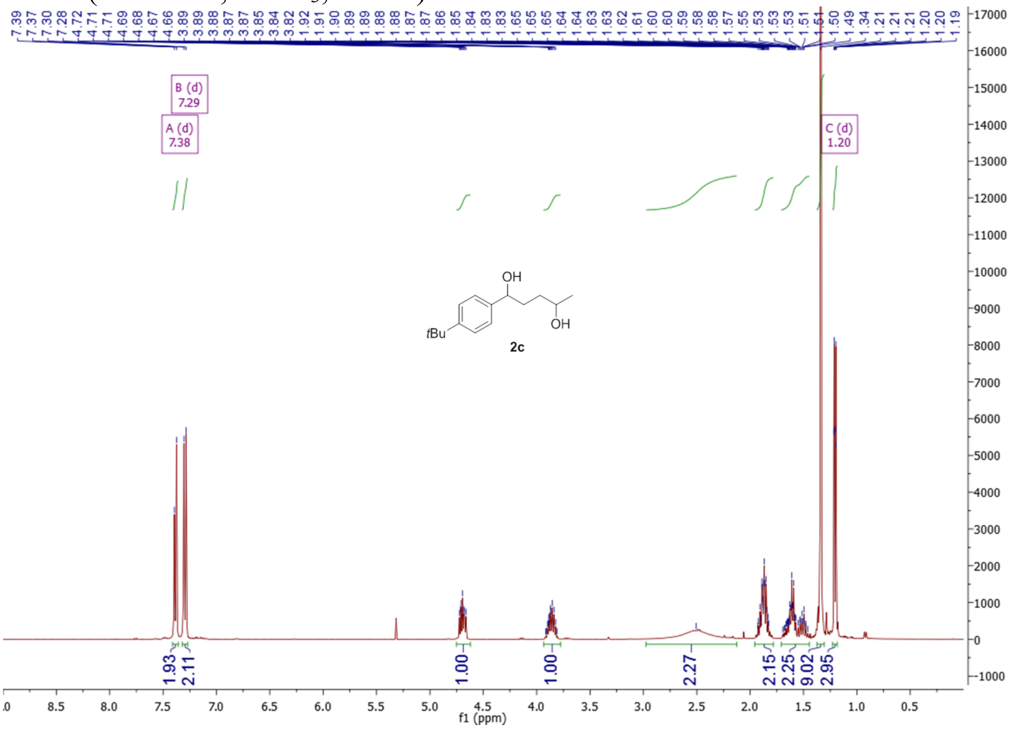

^{13}C{^{1}H} NMR (100 MHz, $CDCl_3$, 298 K)

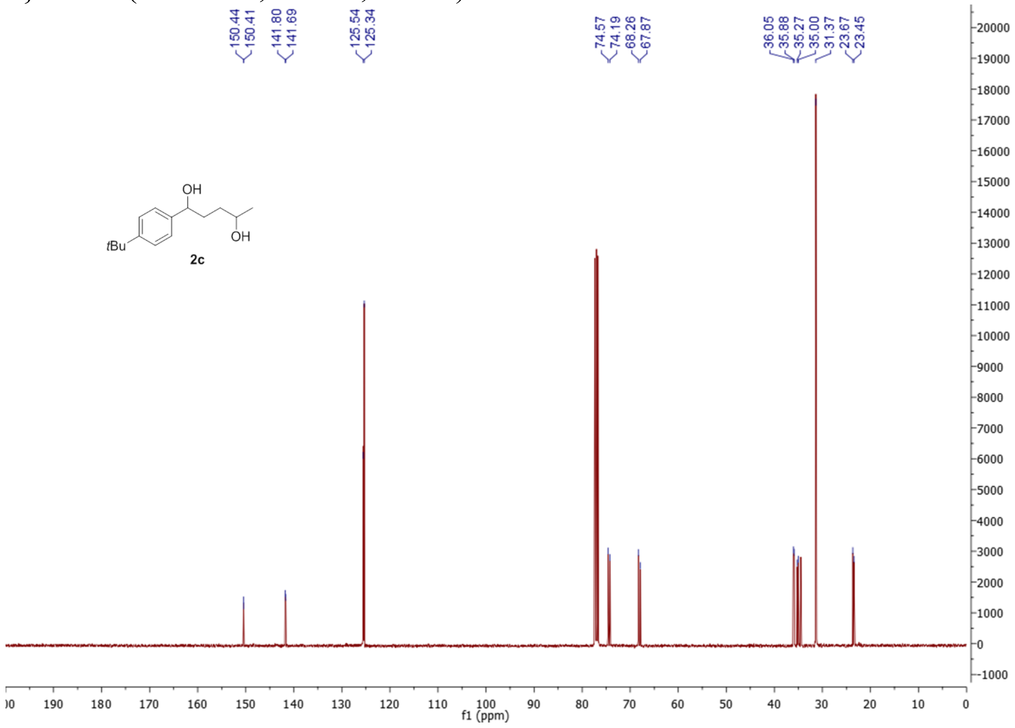

15.4. 1-(*p*-Tolyl)pentane-1,4-diol (2d)

^{1}H NMR (400 MHz, $CDCl_3$, 298 K)

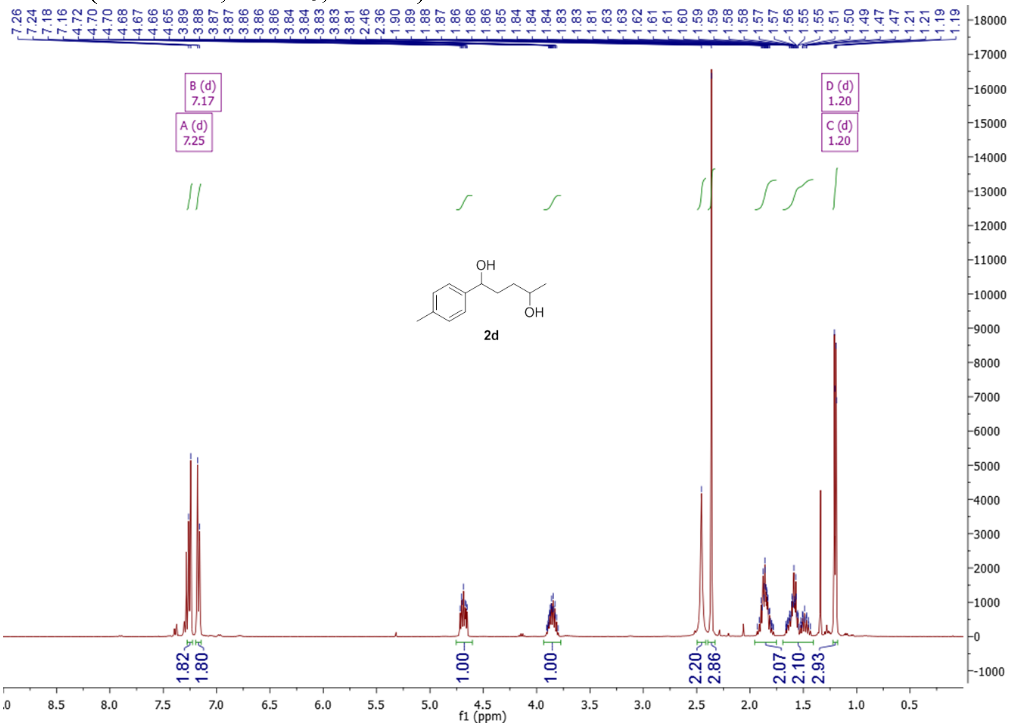

^{13}C{^{1}H} NMR (100 MHz, $CDCl_3$, 298 K)

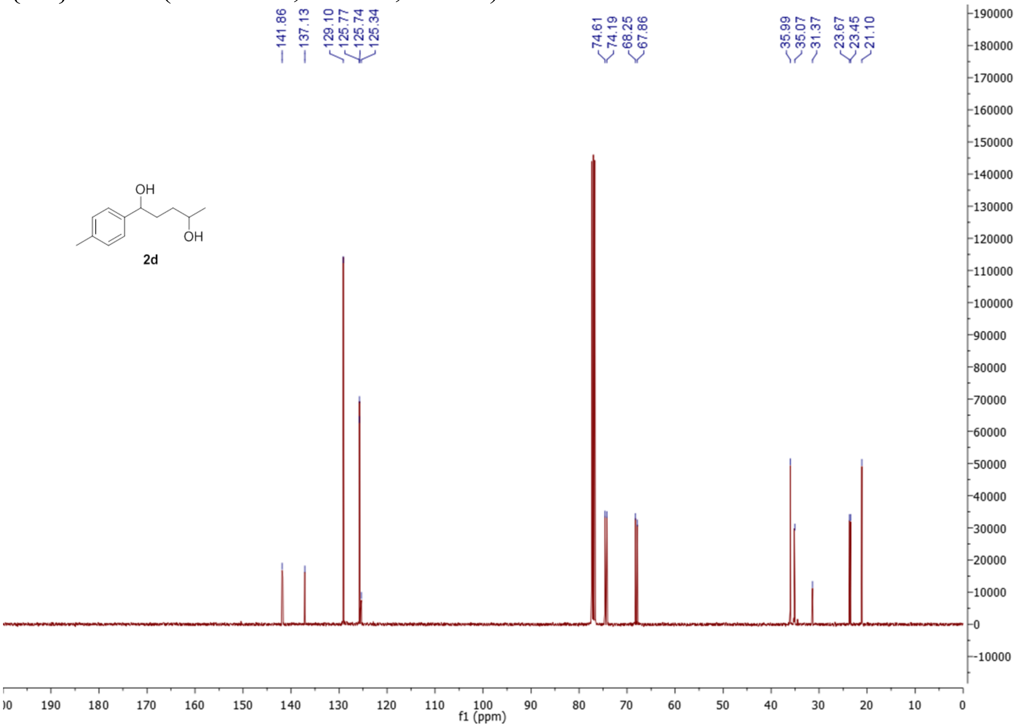

15.5. 1-(*p*-Chlorophenyl)pentane-1,4-diol (2e)

^{1}H NMR (400 MHz, $CDCl_3$, 298 K)

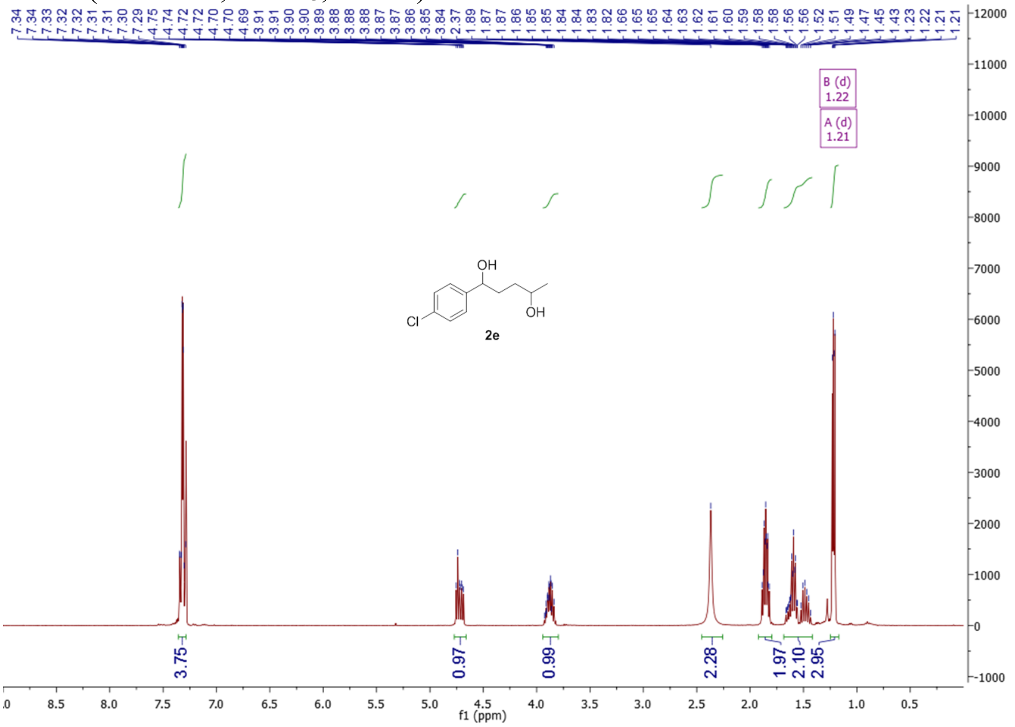

$^{13}C\{^{1}H\}$ NMR (100 MHz, $CDCl_3$, 298 K)

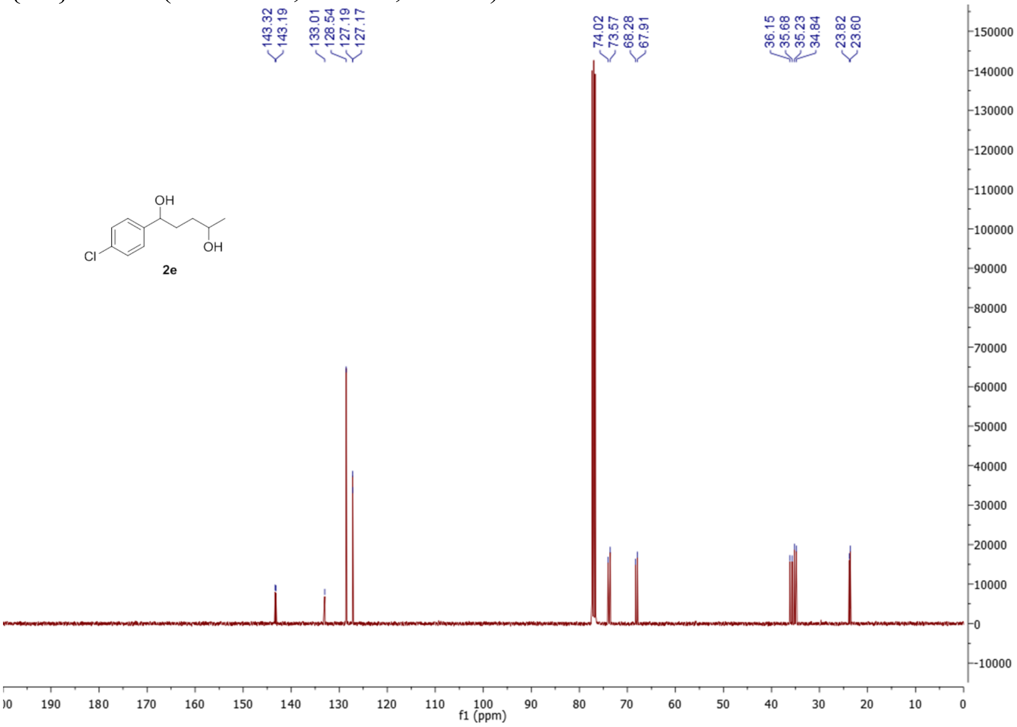

15.6. 1-(*p*-Fluorophenyl)pentane-1,4-diol (2f)

^{1}H NMR (400 MHz, $CDCl_3$, 298 K)

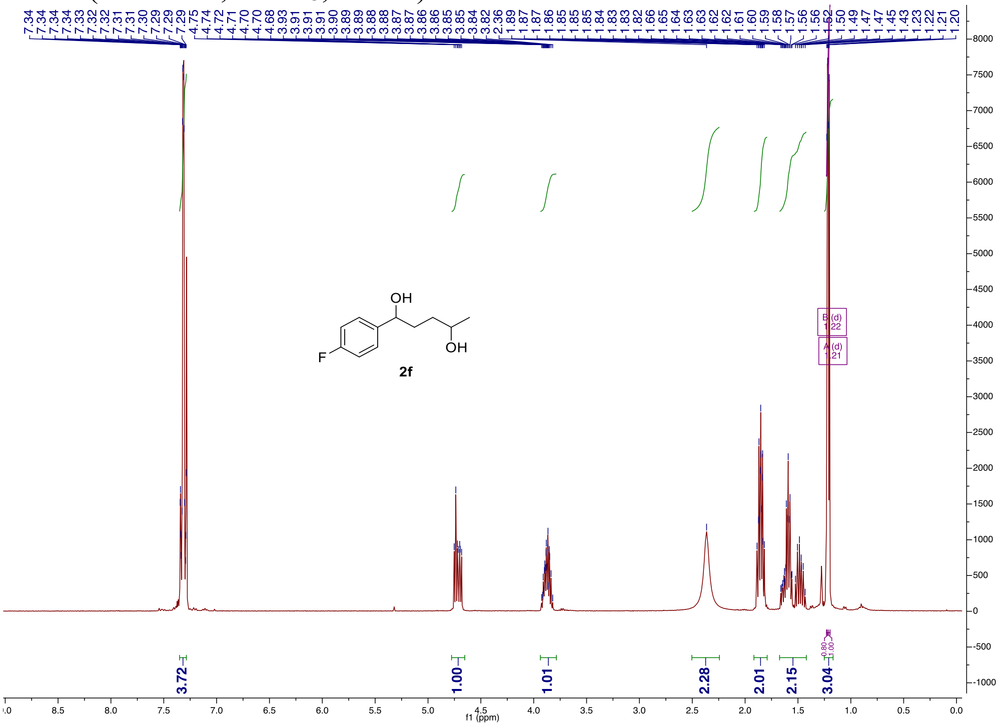

$^{13}C\{^{1}H\}$ NMR (100 MHz, $CDCl_3$, 298 K)

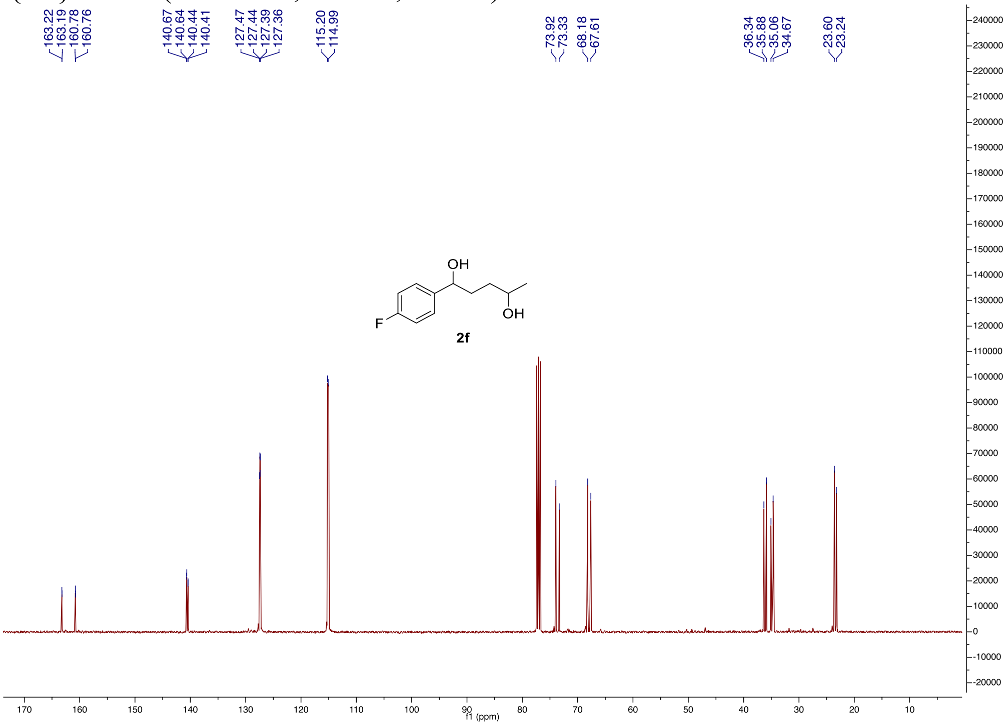

15.7. 1-(*m*-Fluorophenyl)pentane-1,4-diol (2g)

^{1}H NMR (400 MHz, $CDCl_3$, 298 K)

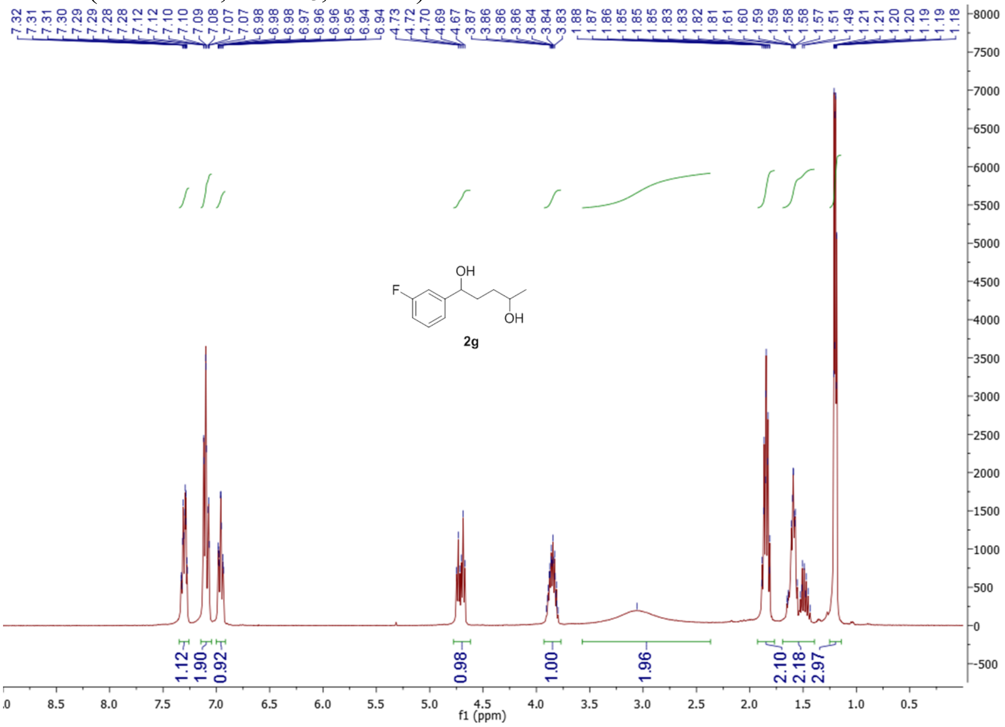

$^{13}C\{^{1}H\}$ NMR (100 MHz, $CDCl_3$, 298 K)

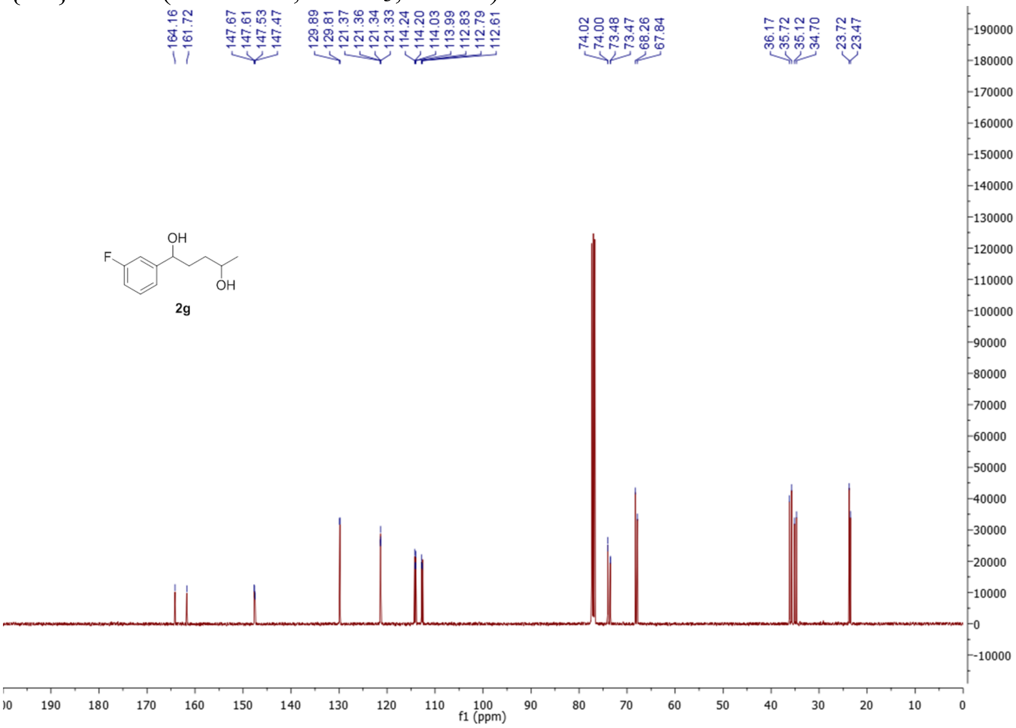

15.8. 1-(*p*-Bromophenyl)pentane-1,4-diol (2h)

^{1}H NMR (400 MHz, $CDCl_3$, 298 K)

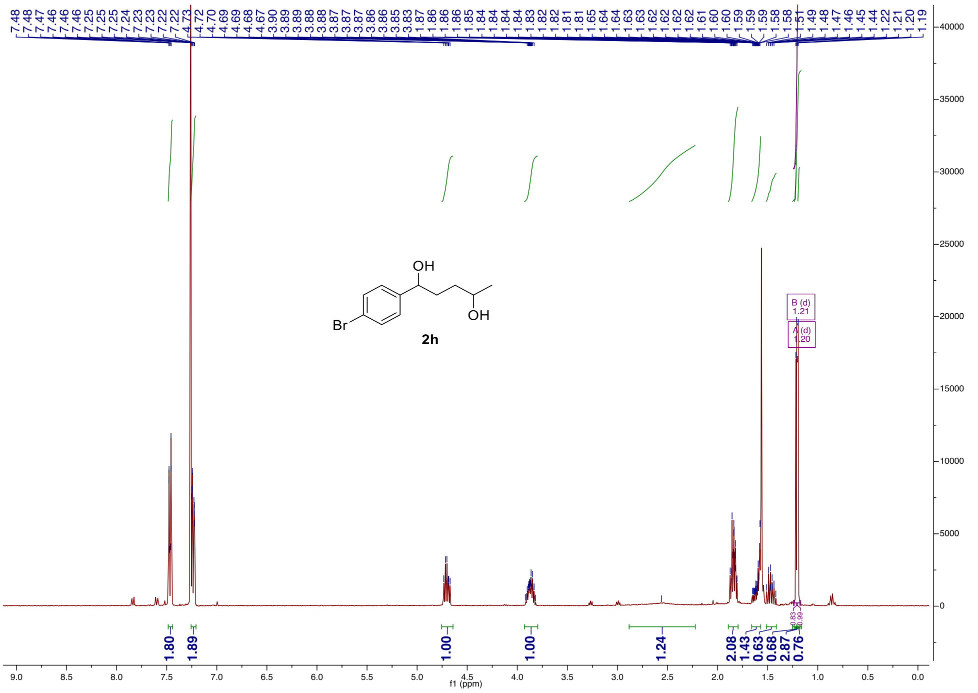

^{13}C{^{1}H} NMR (100 MHz, $CDCl_3$, 298 K)

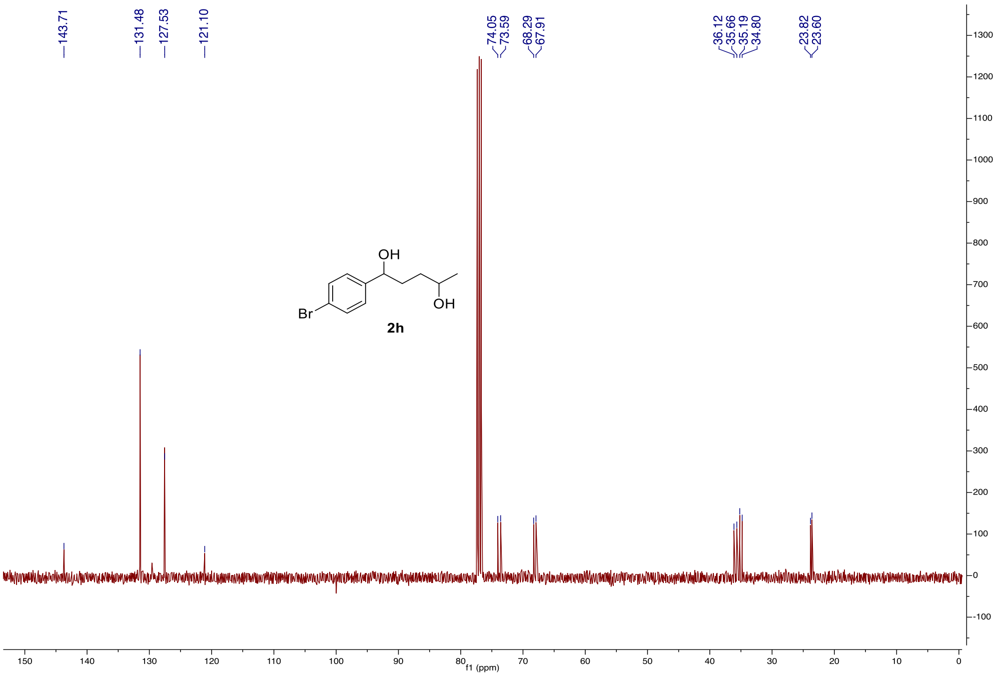

15.9. 1-(2-Naphtalenyl)pentane-1,4-diol (2i)

^1H NMR (400 MHz, $CDCl_3$, 298 K)

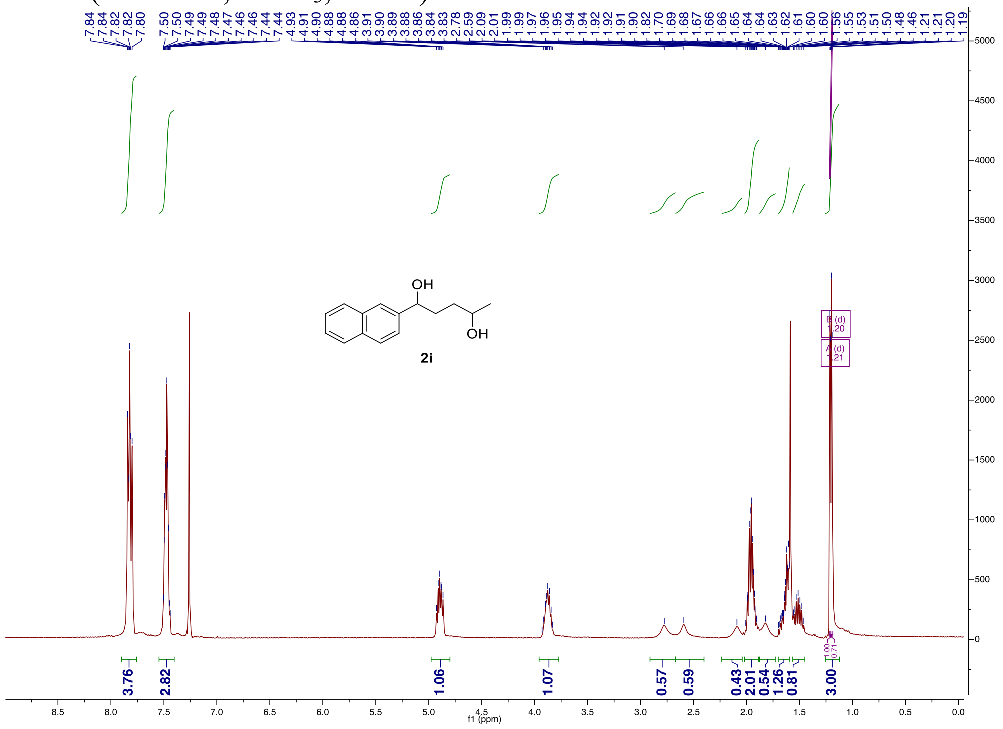

$^{13}C\{^1H\}$ NMR (100 MHz, $CDCl_3$, 298 K)

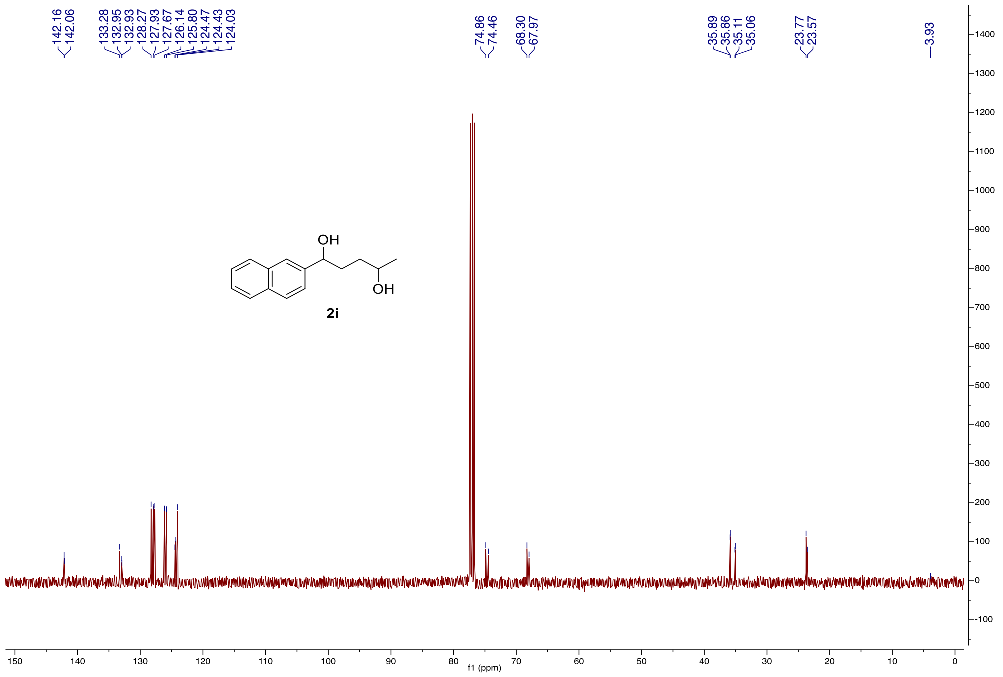

15.10. 1,4-Diphenylbutane-1,4-diol (2j)

^{1}H NMR (400 MHz, $CDCl_3$, 298 K)

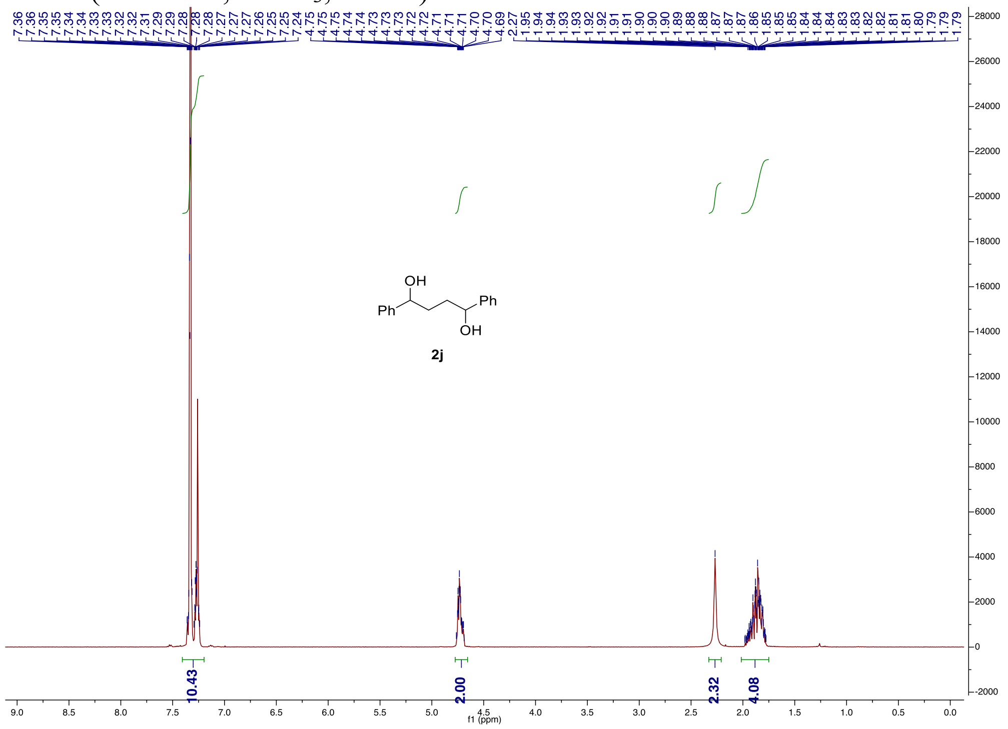

$^{13}C\{^{1}H\}$ NMR (100 MHz, $CDCl_3$, 298 K)

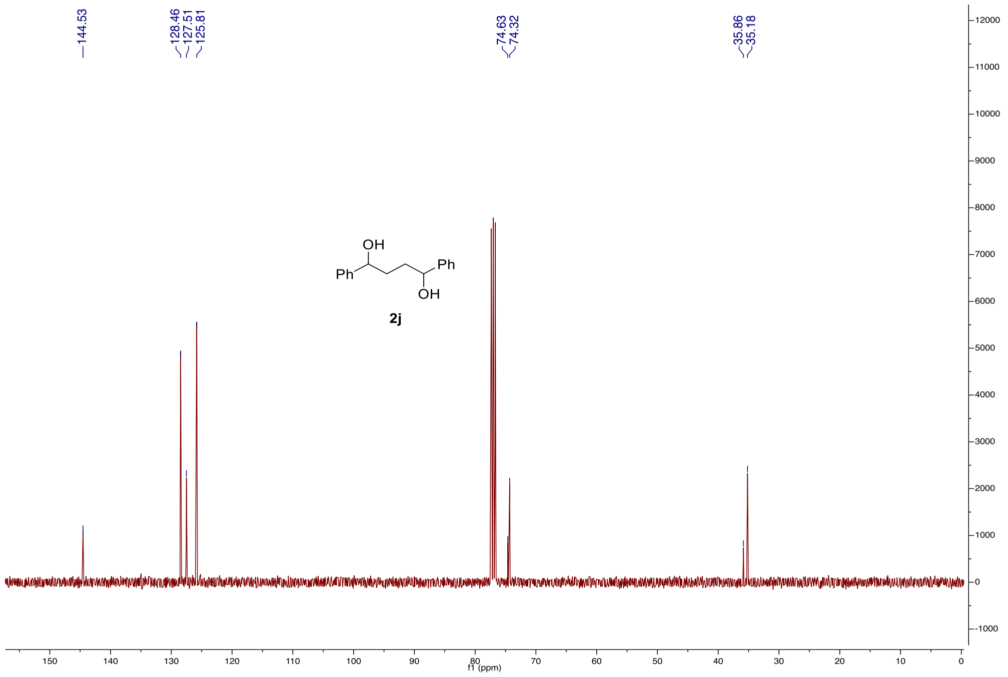

15.11. 1-Phenylhexane-1,5-diol (2m)

^{1}H NMR (400 MHz, $CDCl_3$, 298 K)

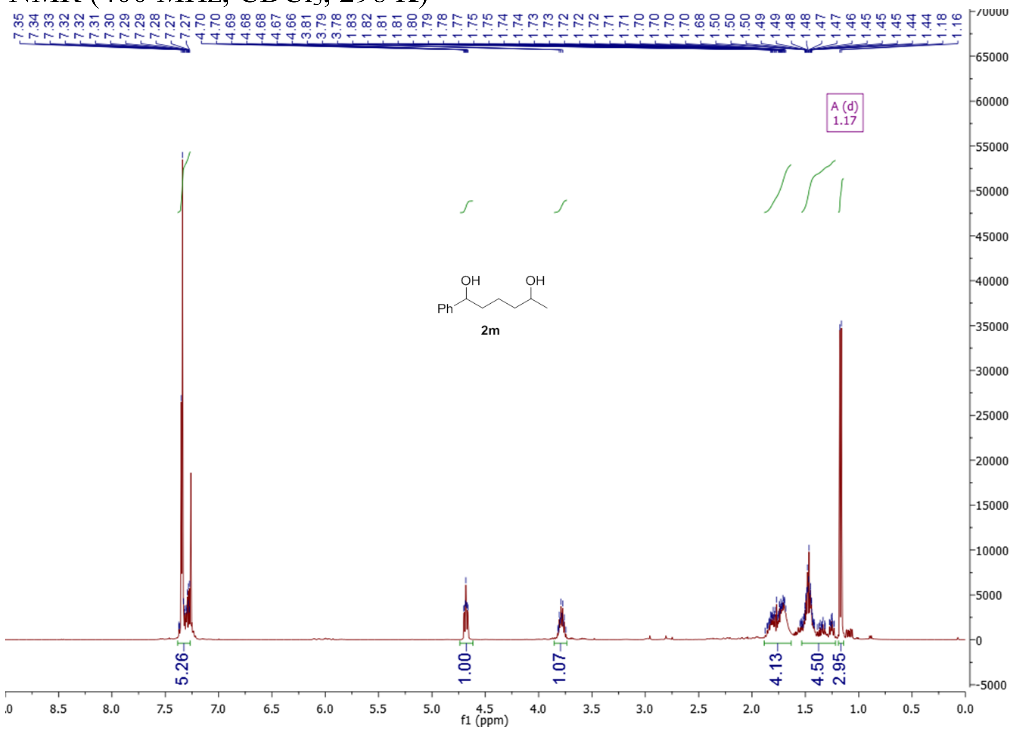

$^{13}C\{^{1}H\}$ NMR (100 MHz, $CDCl_3$, 298 K)

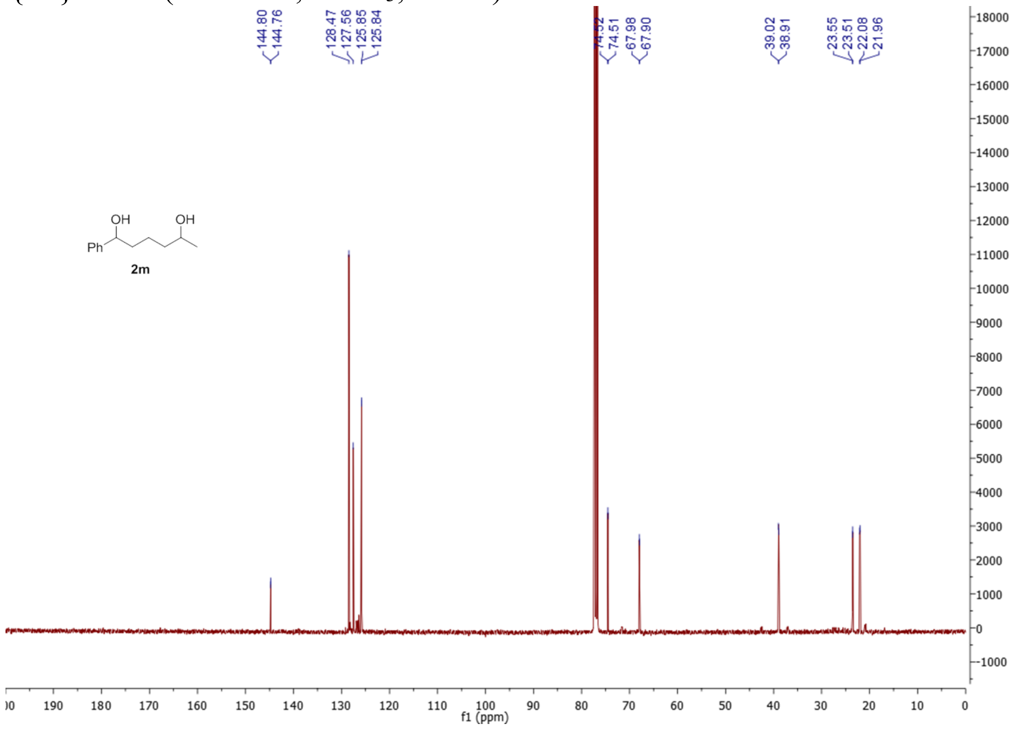

15.12. 1,5-Diphenylpentane-1,5-diol (2n)

^{1}H NMR (400 MHz, $CDCl_3$, 298 K)

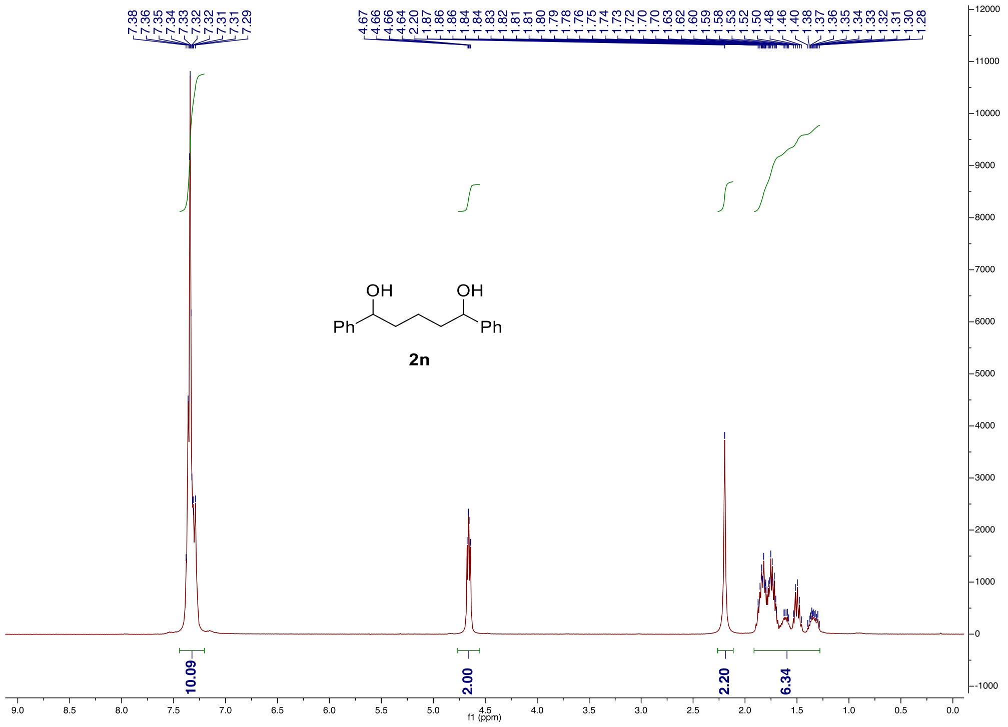

$^{13}C\{^{1}H\}$ NMR (100 MHz, $CDCl_3$, 298 K)

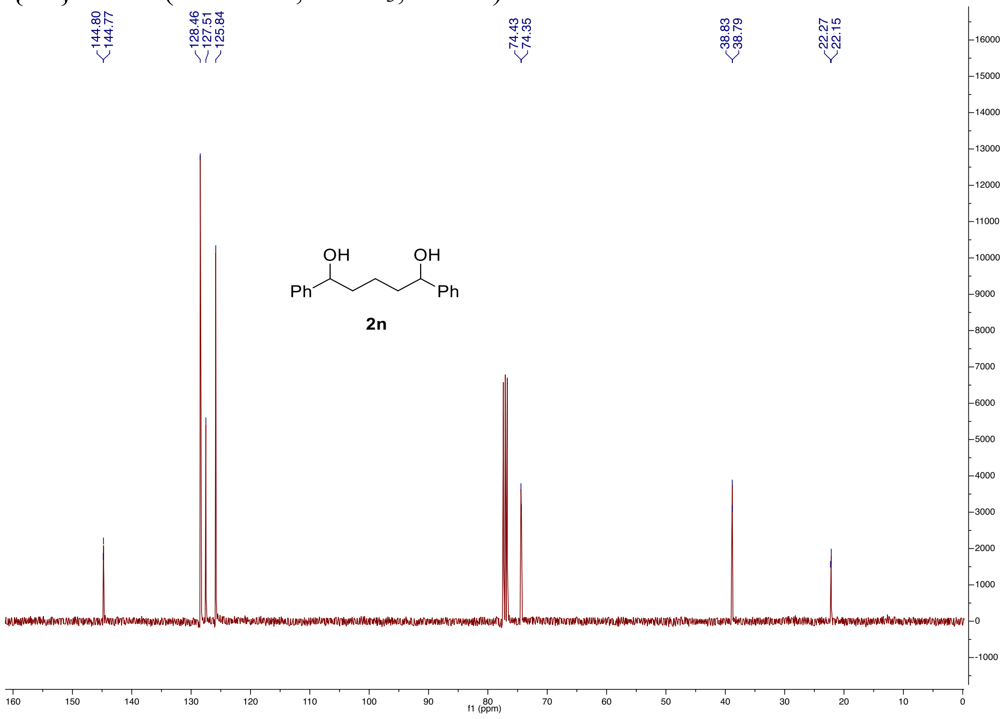

15.13. 1-Phenylpentane-1,4-d_2-1,4-diol (2a-d_2)

^{1}H NMR (400 MHz, $CDCl_3$, 298 K)

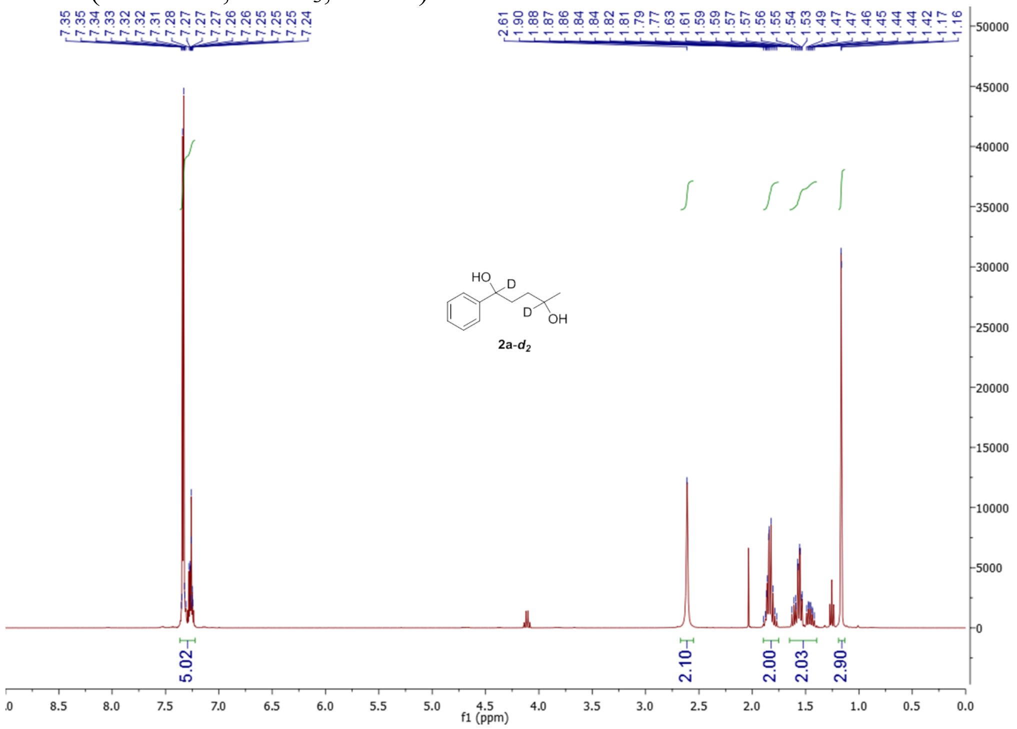

$^{13}C\{^{1}H\}$ NMR (100 MHz, $CDCl_3$, 298 K)

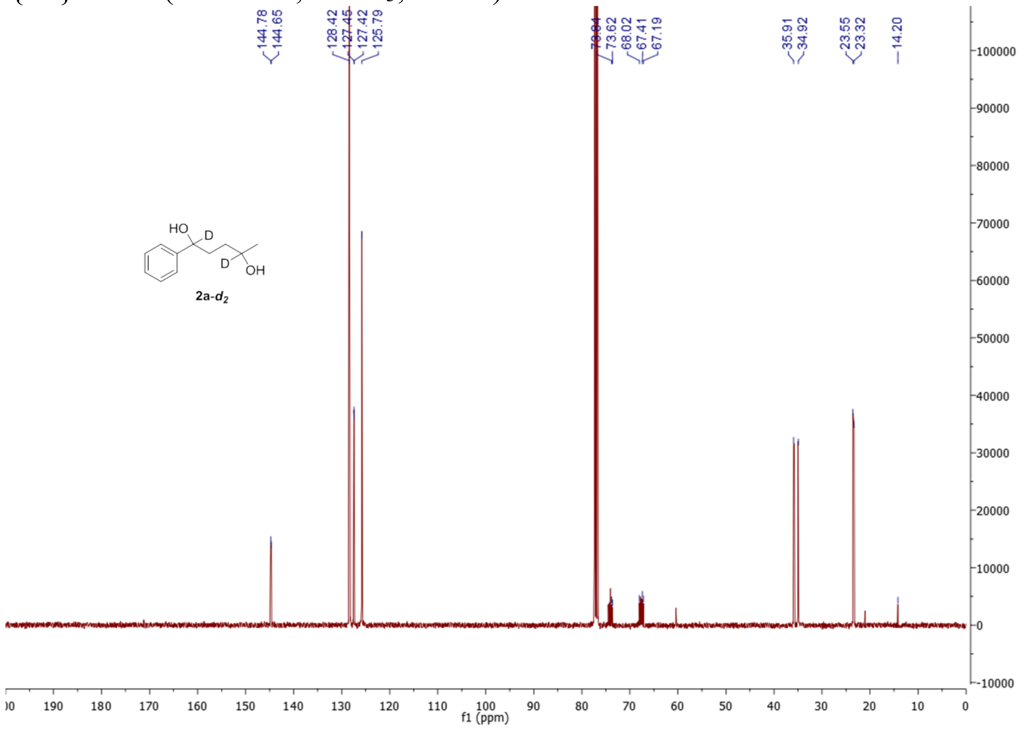

15.14. 1-(*p*-Methoxyphenyl)pentane-1,4-d2-1,4-diol (2b-*d*$_2$)

^{1}H NMR (500 MHz, $CDCl_3$, 298 K)

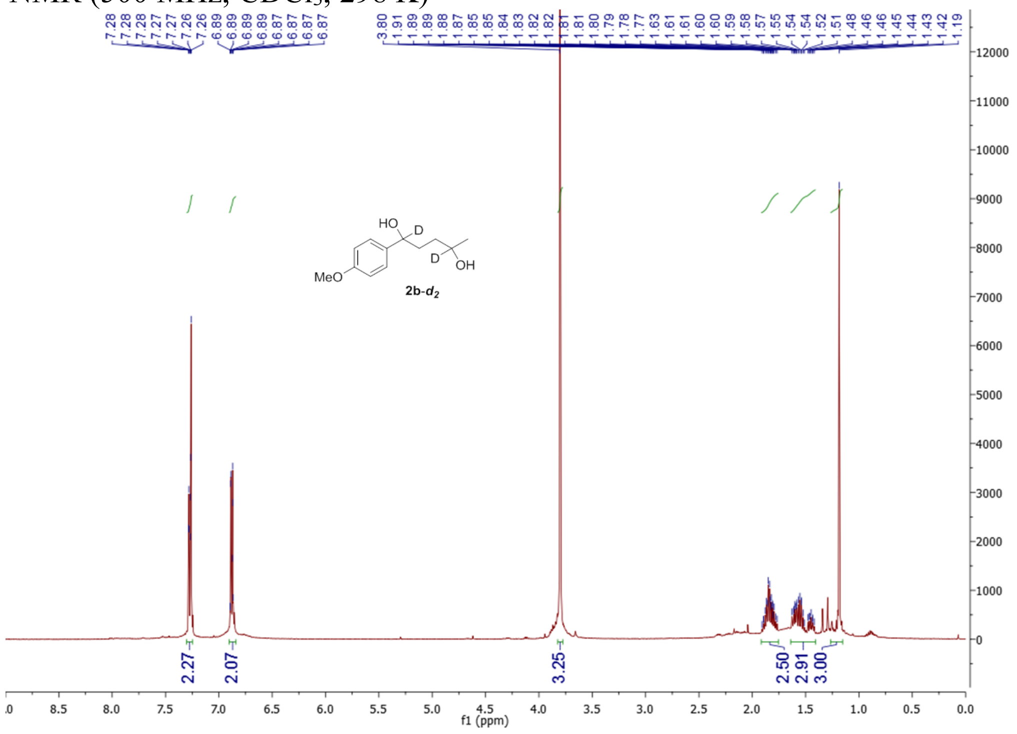

^{13}C{^{1}H} NMR (125 MHz, $CDCl_3$, 298 K)

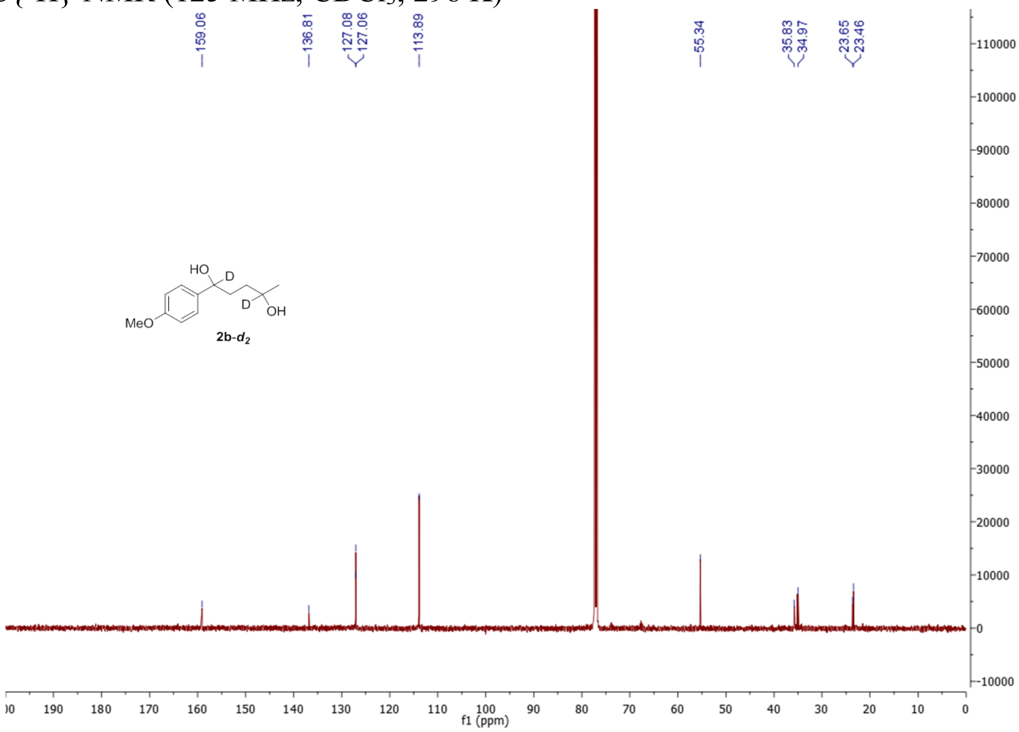

15.15. 2-Phenyl-5-methyltetrahydrofuran (3a)

^{1}H NMR (400 MHz, $CDCl_3$, 298 K)

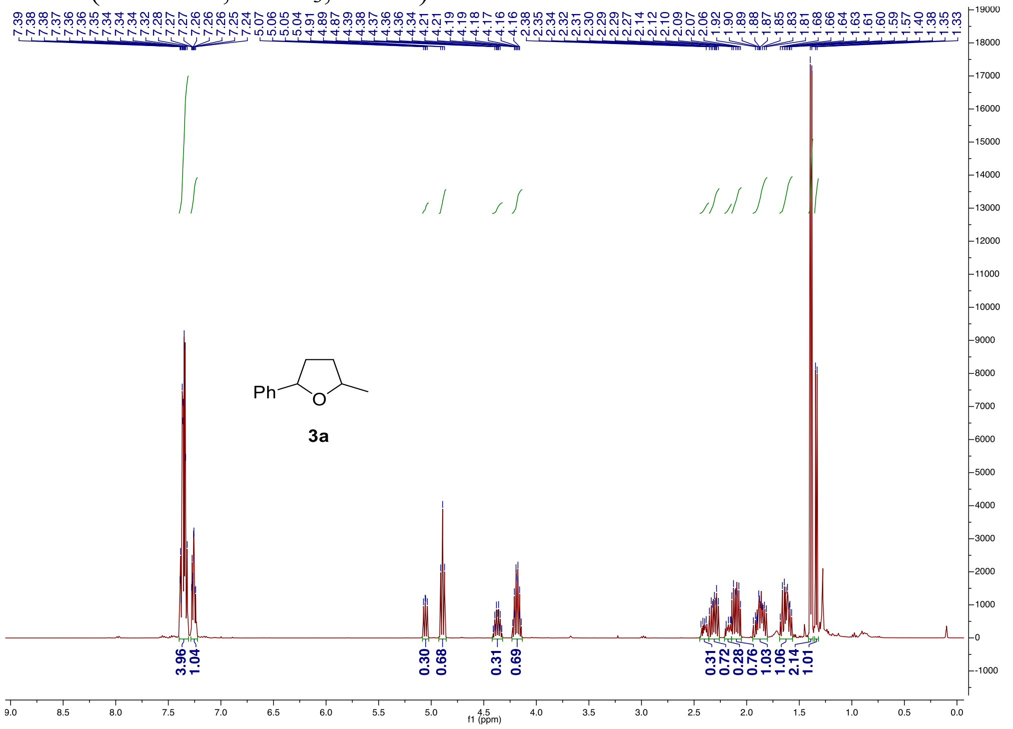

^{13}C{^{1}H} NMR (100 MHz, $CDCl_3$, 298 K)

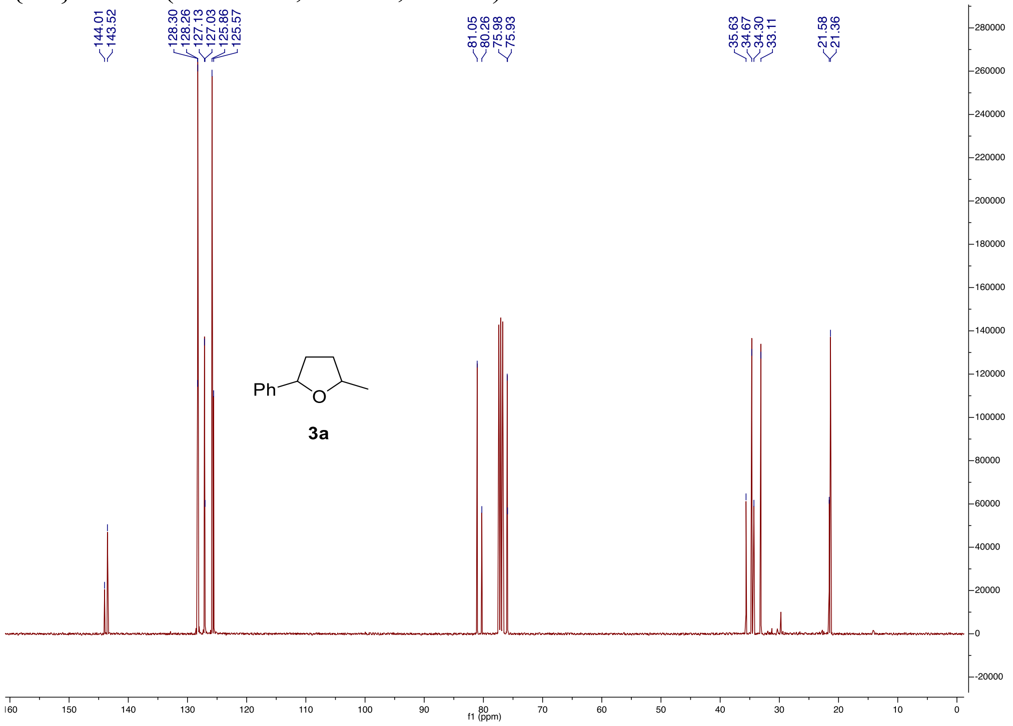

15.16. 2-(*p*-Methoxyphenyl)-5-methyltetrahydrofuran (3b)

^{1}H NMR (400 MHz, $CDCl_3$, 298 K)

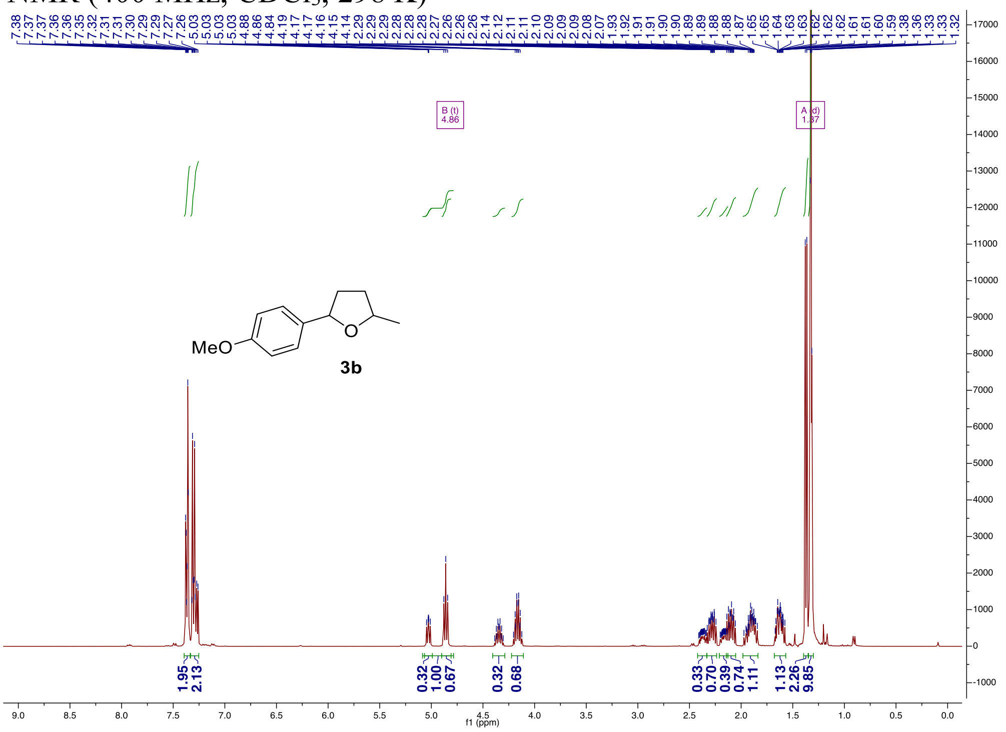

15.17. 2-(*p-tert*-Butylphenyl)-5-methyltetrahydrofuran (3c)

^{1}H NMR (400 MHz, $CDCl_3$, 298 K)

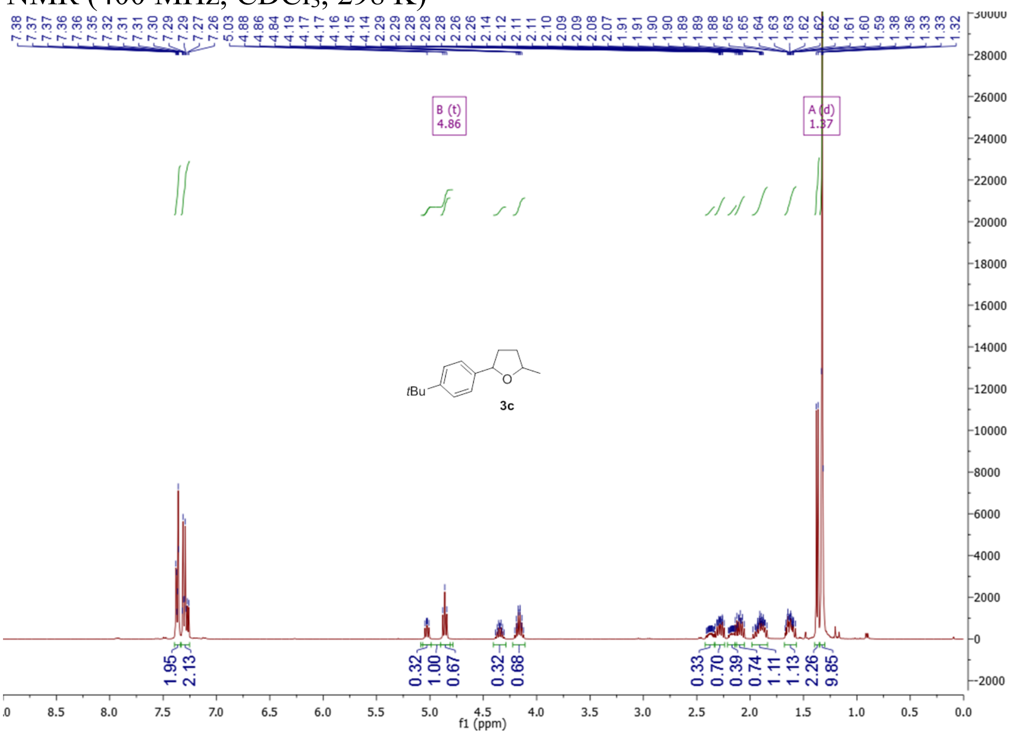

$^{13}C\{^{1}H\}$ NMR (100 MHz, $CDCl_3$, 298 K)

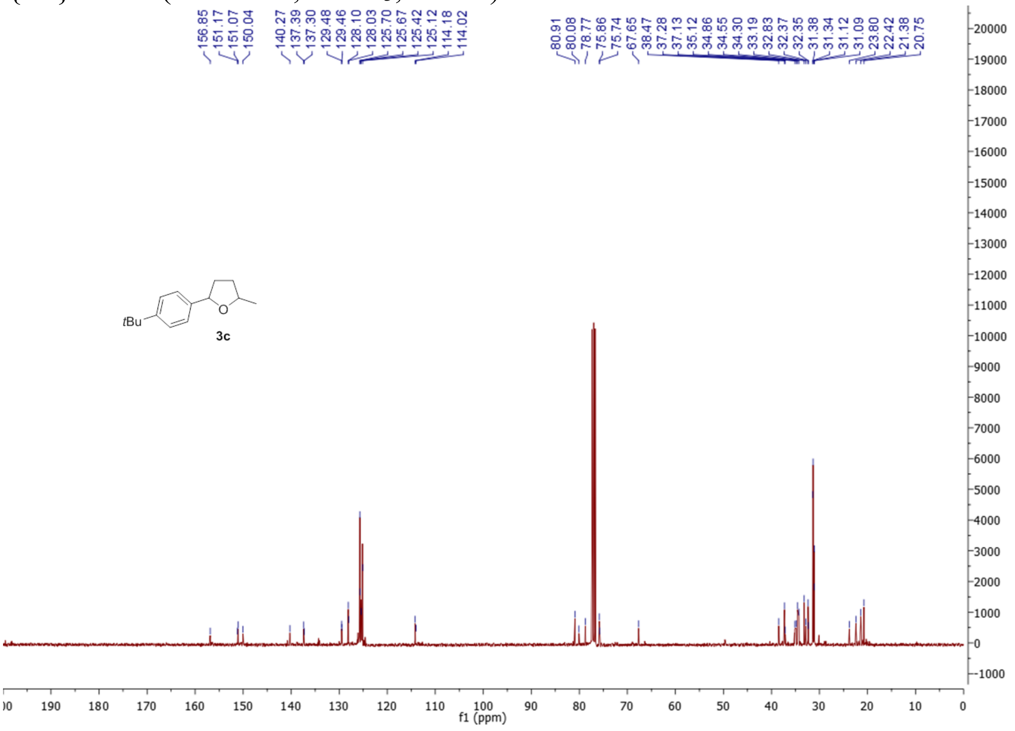

15.18. 2-(*p*-Methylphenyl)-5-methyltetrahydrofuran (3d)

^{1}H NMR (400 MHz, $CDCl_3$, 298 K)

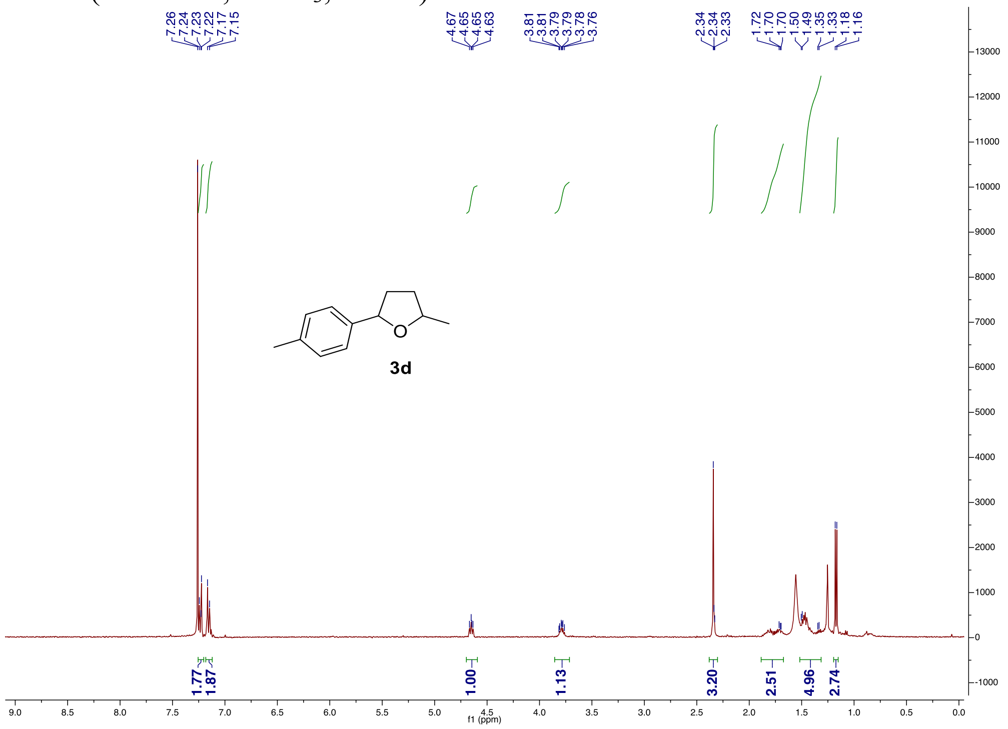

^{13}C{^{1}H} NMR (100 MHz, $CDCl_3$, 298 K)

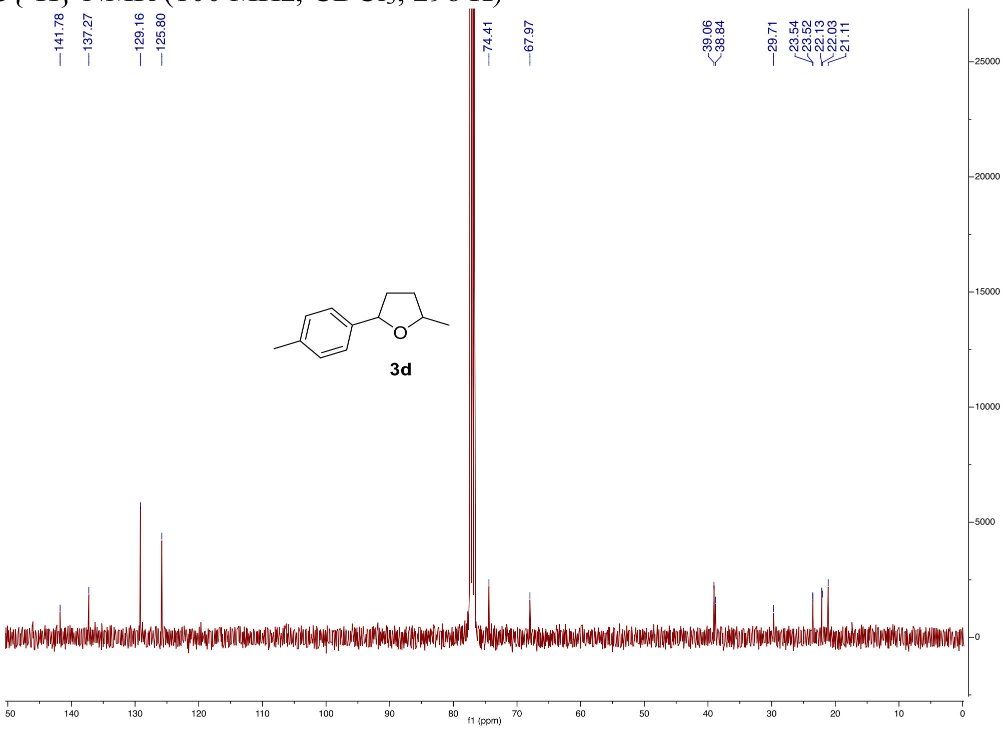

15.19. 2-(*p*-Chlorophenyl)-5-methyltetrahydrofuran (3e)

^{1}H NMR (400 MHz, $CDCl_3$, 298 K)

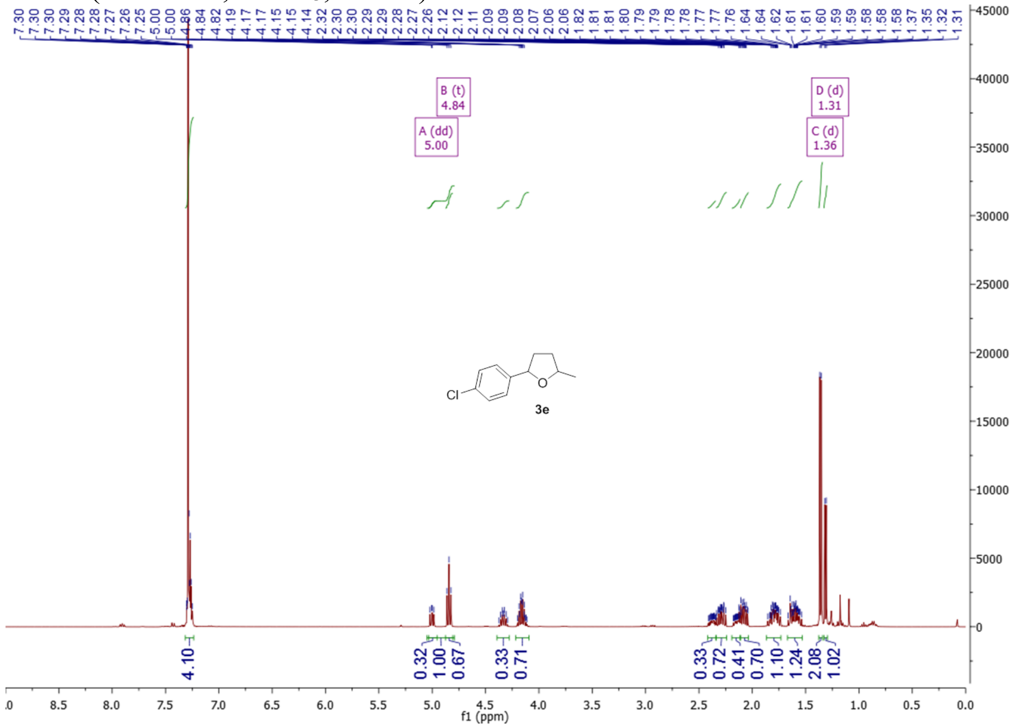

$^{13}C\{^1H\}$ NMR (100 MHz, $CDCl_3$, 298 K)

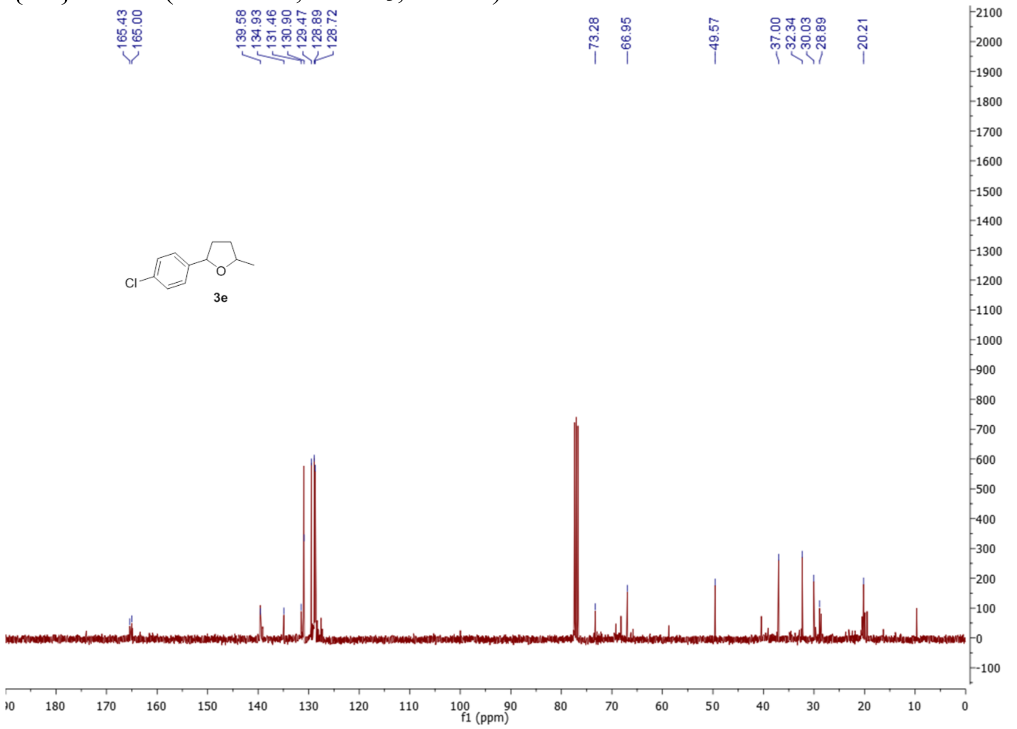

15.20. 2-(*p*-Fluorophenyl)-5-methyltetrahydrofuran (3f)

^{1}H NMR (400 MHz, $CDCl_3$, 298 K)

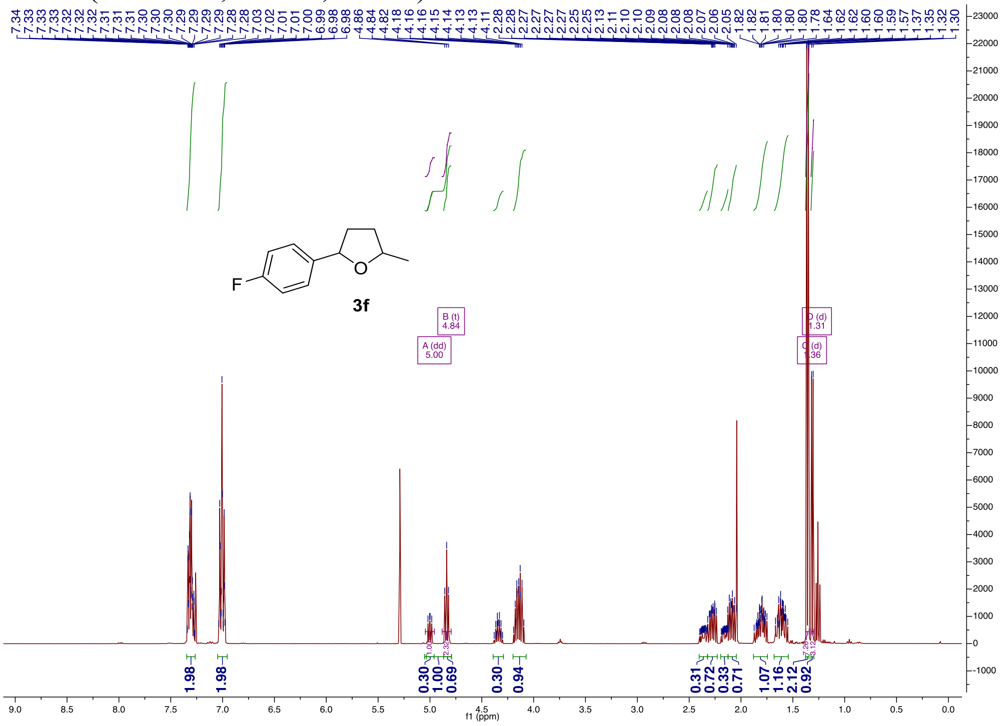

$^{13}C\{^{1}H\}$ NMR (100 MHz, $CDCl_3$, 298 K)

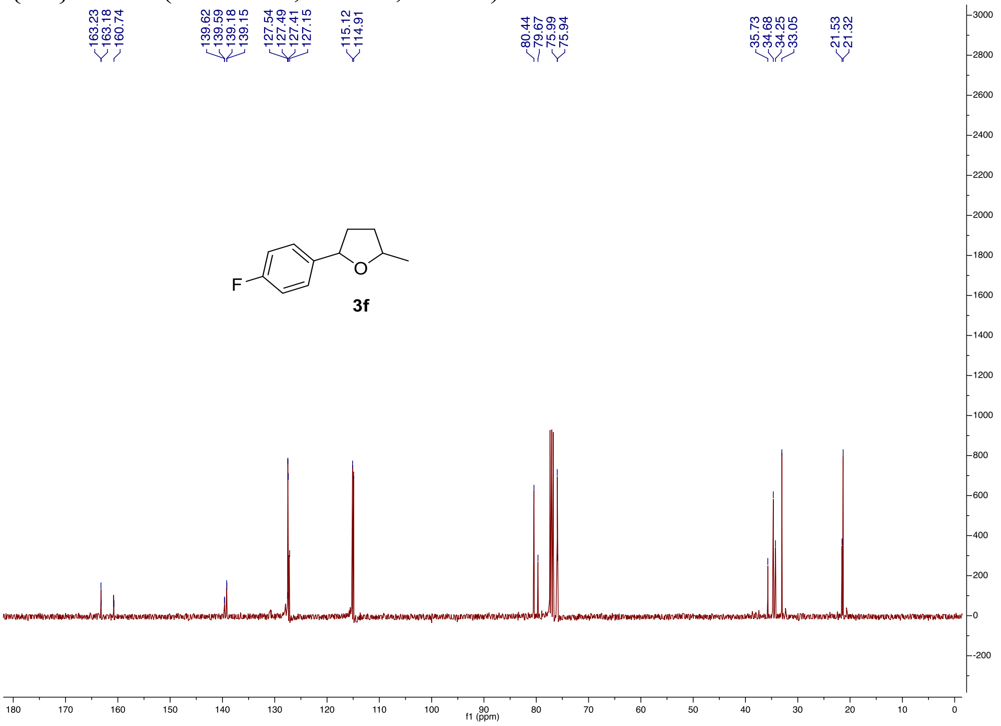

15.21. 2-(*m*-Fluorophenyl)-5-methyltetrahydrofuran (3g)

^{1}H NMR (400 MHz, $CDCl_3$, 298 K)

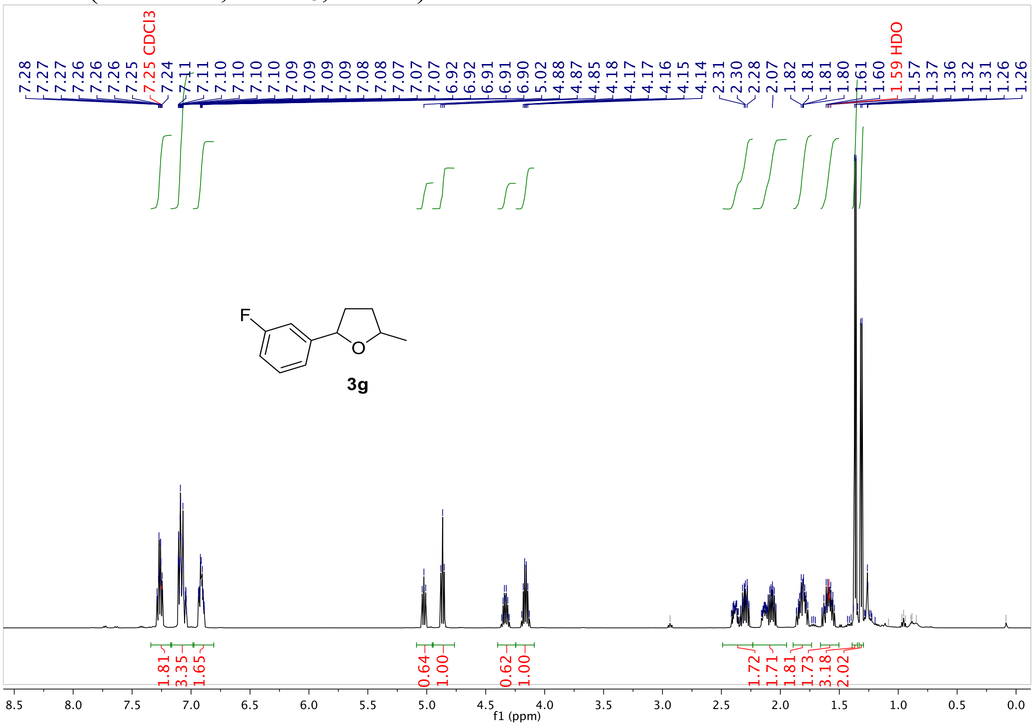

$^{13}C\{^{1}H\}$ NMR (100 MHz, $CDCl_3$, 298 K)

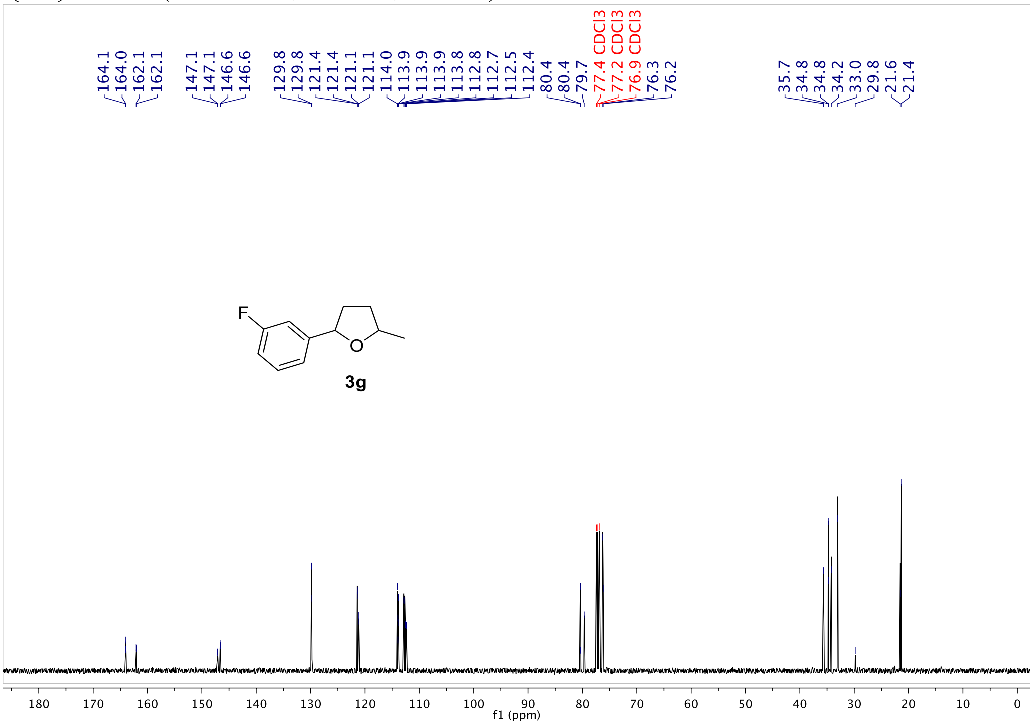

15.22. 2-(*p*-Bromophenyl)-5-methyltetrahydrofuran (3h)

^{1}H NMR (400 MHz, CDCl$_3$, 298 K)

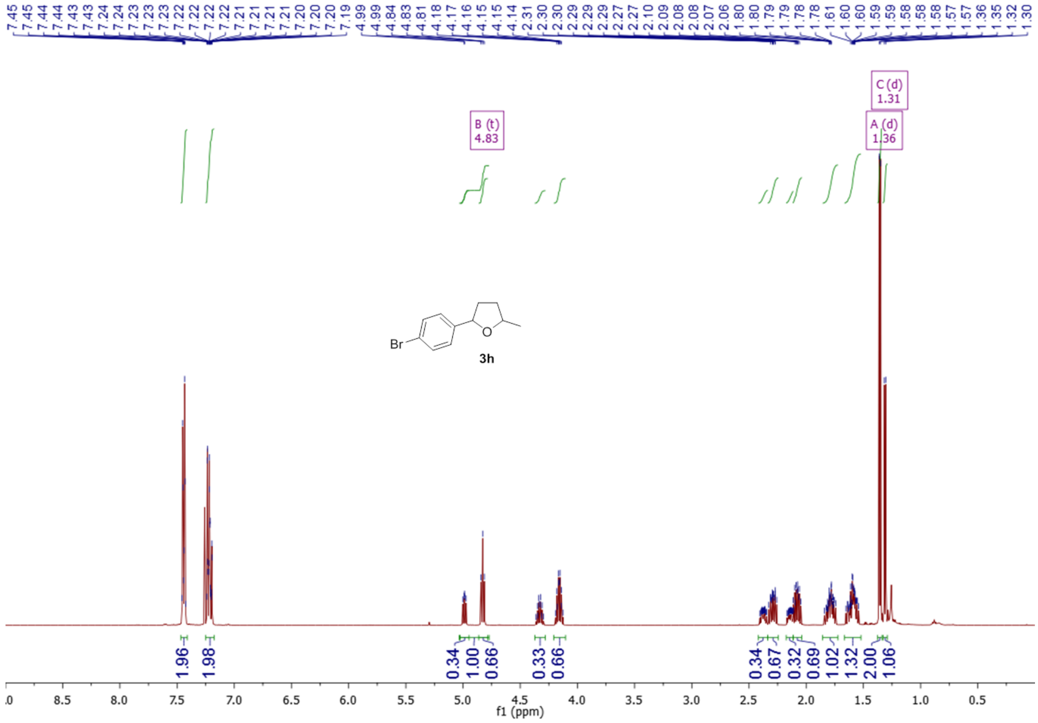

^{13}C{^{1}H} NMR (100 MHz, CDCl$_3$, 298 K)

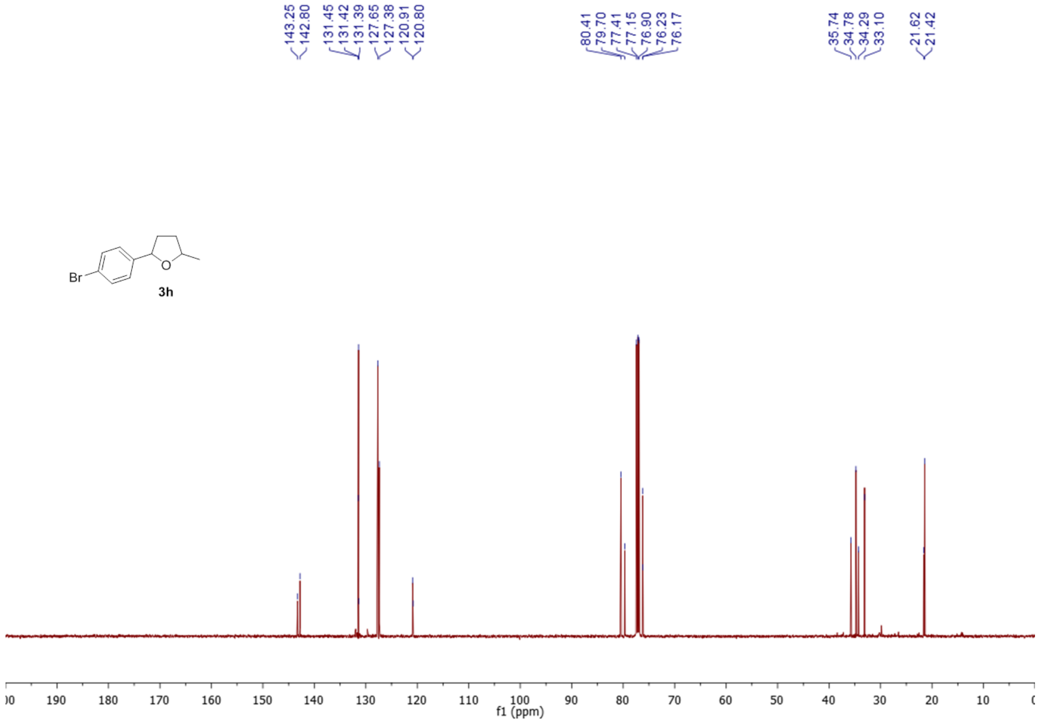

15.23. 2-(2-Naphtalenyl)-5-methyltetrahydrofuran (3i)

^{1}H NMR (400 MHz, $CDCl_3$, 298 K)

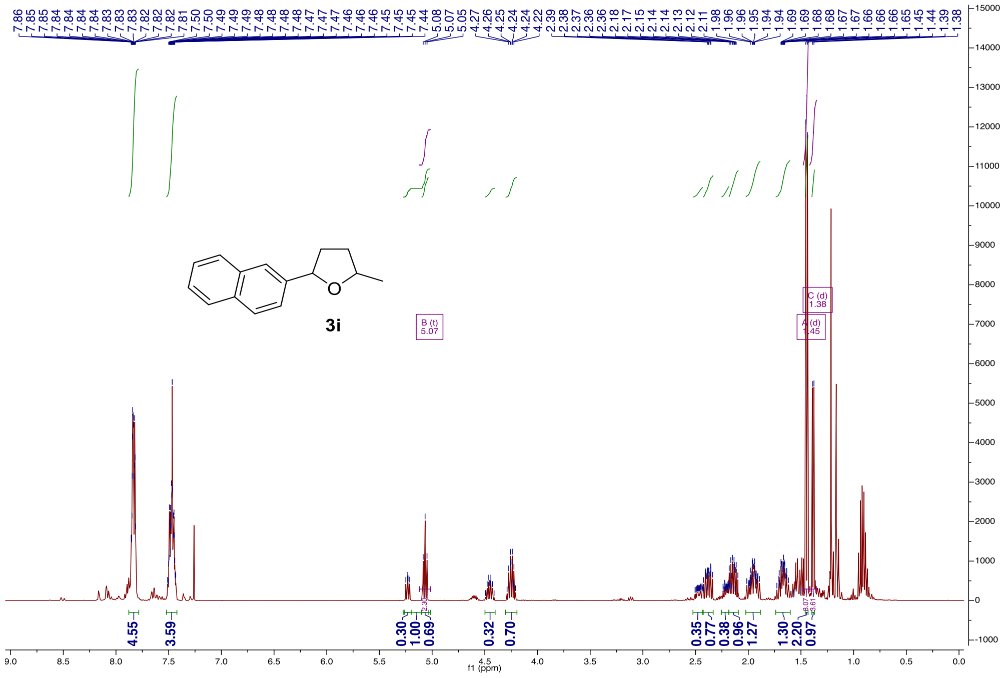

$^{13}C\{^1H\}$ NMR (100 MHz, $CDCl_3$, 298 K)

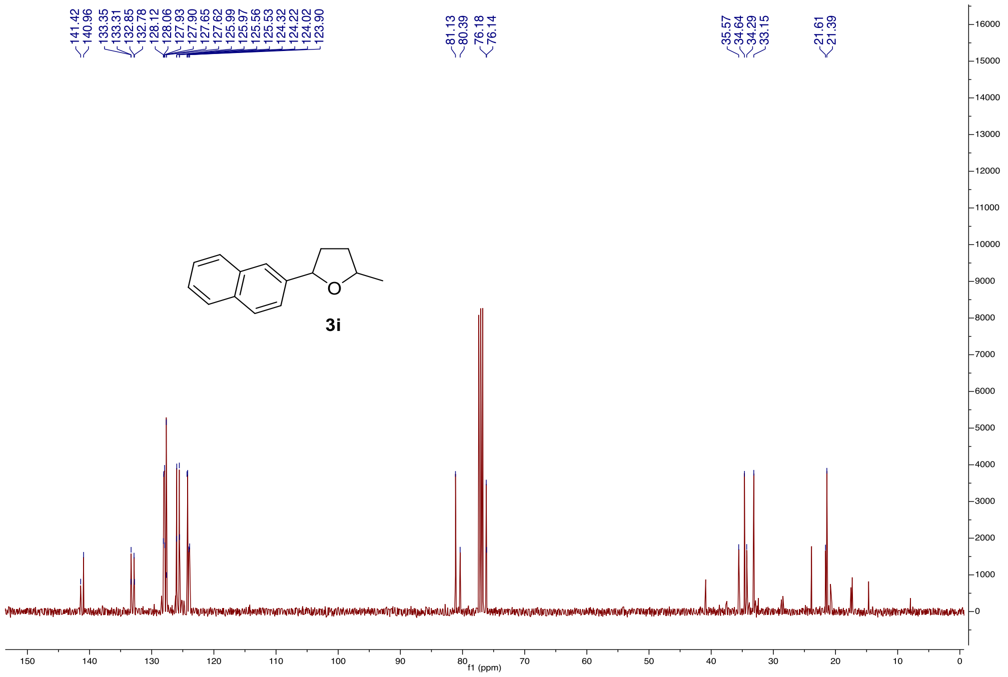

15.24. 2,5-Dipethyltetrahydrofuran (3j)

^{1}H NMR (400 MHz, $CDCl_3$, 298 K)

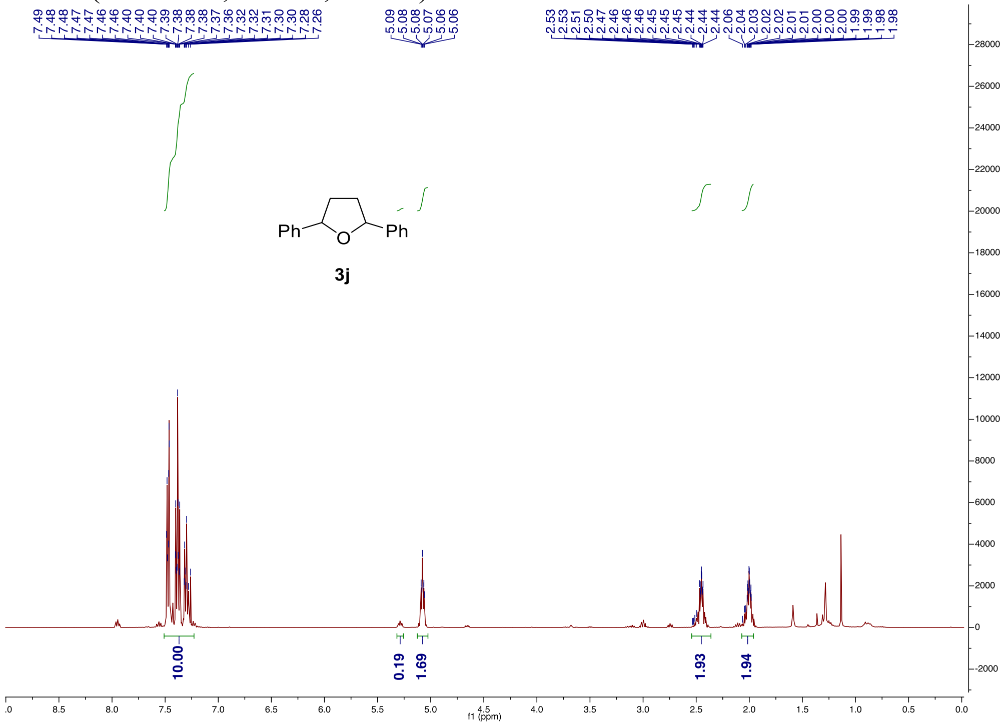

^{13}C{^{1}H} NMR (100 MHz, $CDCl_3$, 298 K)

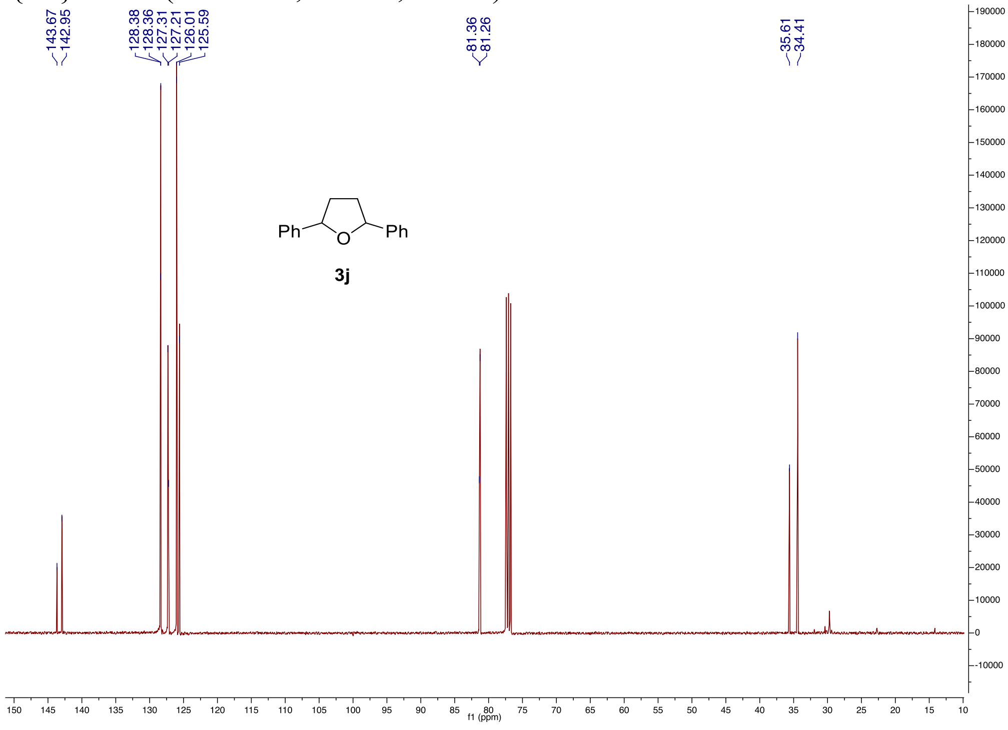

15.25. 2-Phenyltetrahydrofuran (3l)

^{1}H NMR (400 MHz, $CDCl_3$, 298 K)

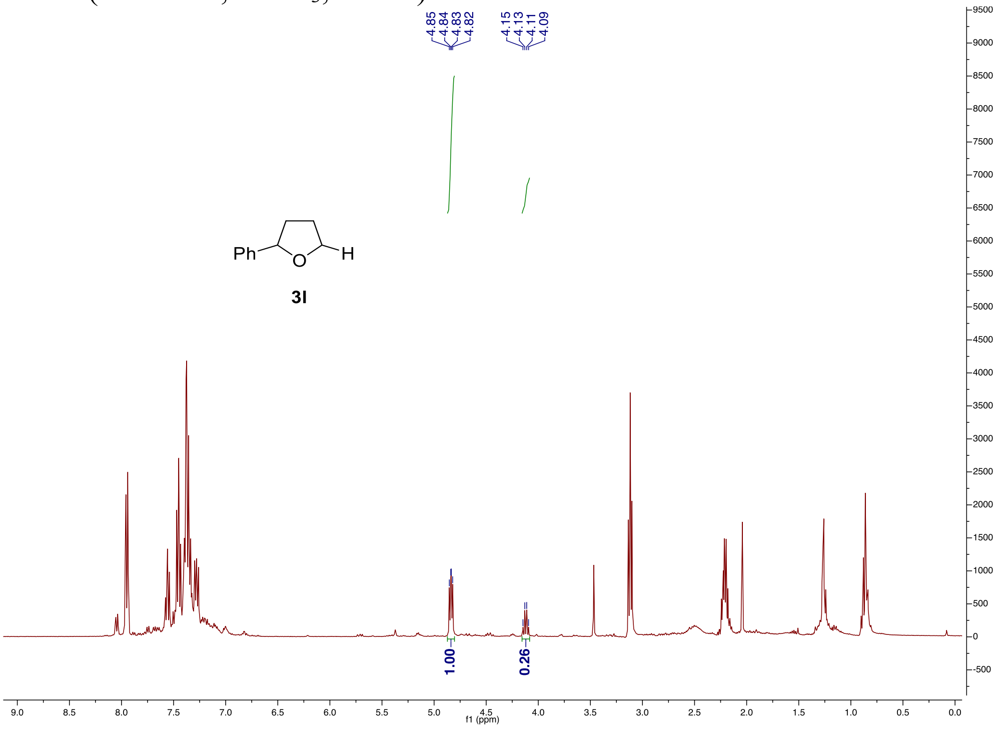

15.26. 2-Methyl-5-phenyl-methyltetrahydrofuran-2,5-d_2 (3a-d_2)

^{1}H NMR (400 MHz, $CDCl_3$, 298 K)

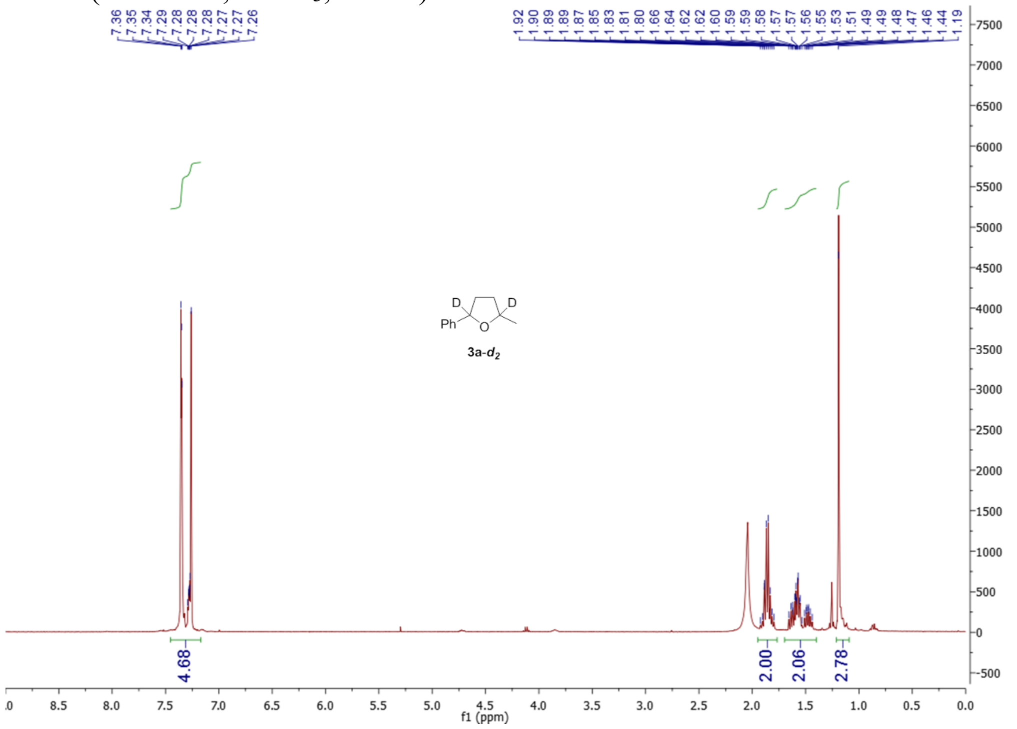

^{13}C{^{1}H} NMR (100 MHz, $CDCl_3$, 298 K)

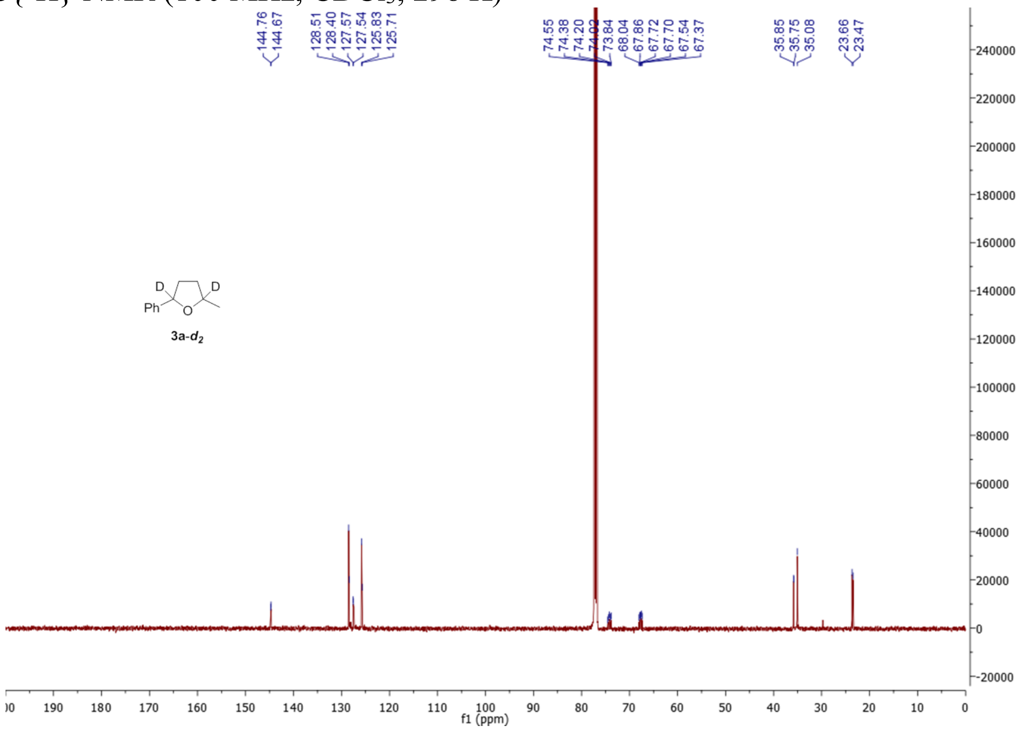

15.27. 2-(*p*-Methoxyphenyl)-5-methyltetrahydrofuran-2,5-d_2 (3b-d_2)

^{1}H NMR (400 MHz, $CDCl_3$, 298 K)

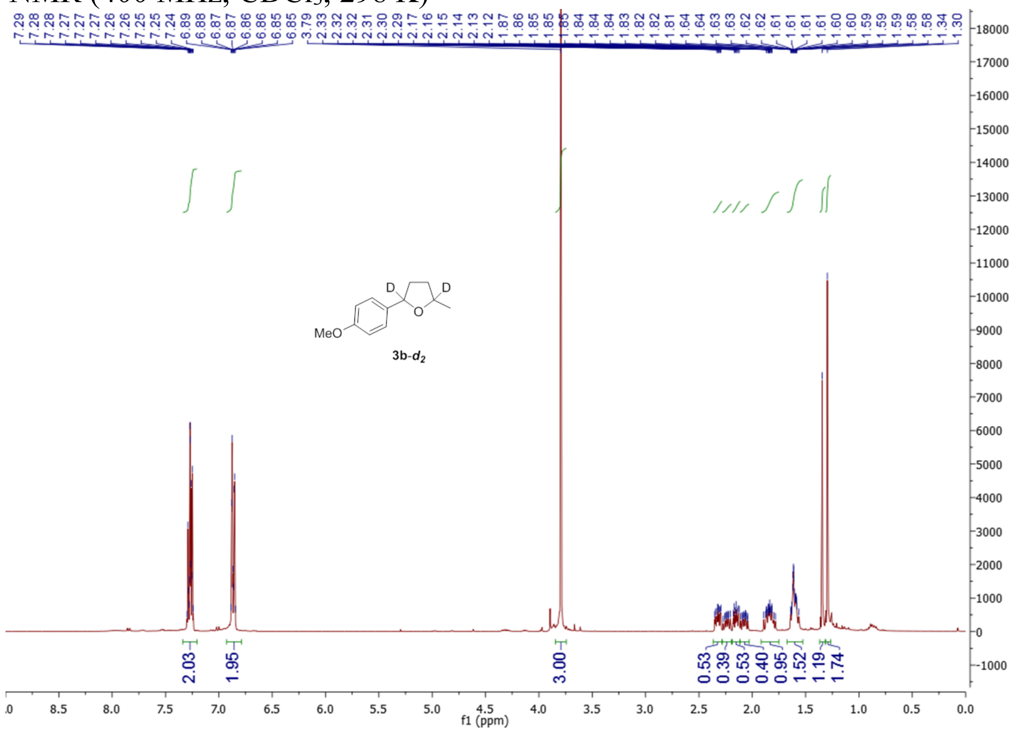

$^{13}C\{^1H\}$ NMR (100 MHz, $CDCl_3$, 298 K)

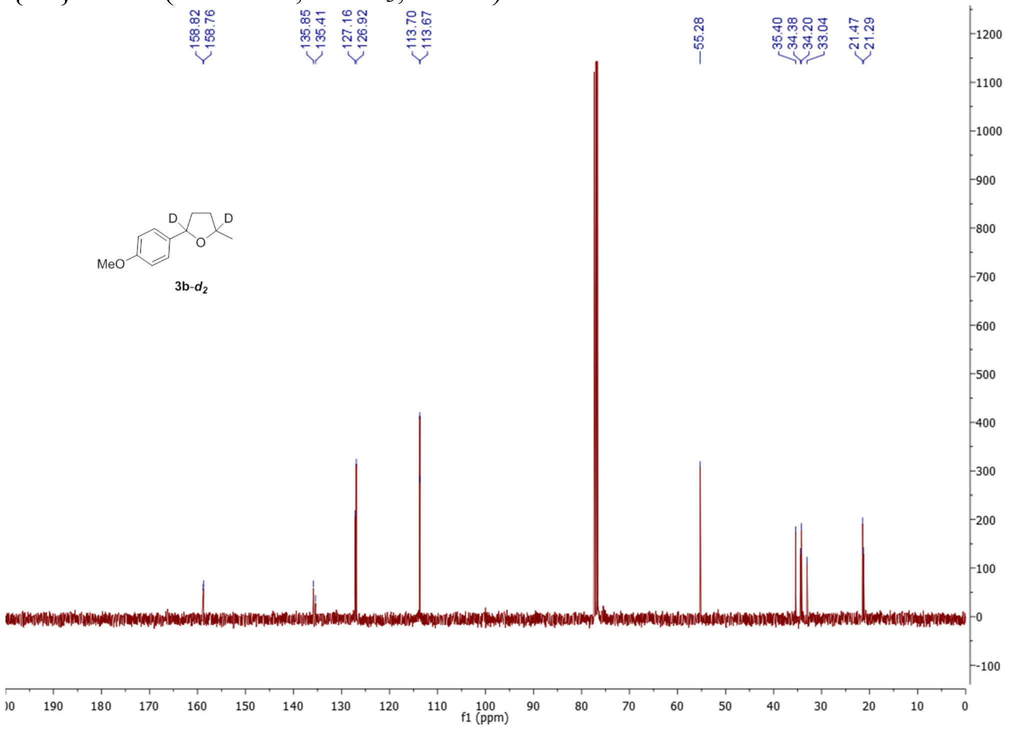

15.28. 2-Phenyl-6-methyltetrahydropyran (3m)

^{1}H NMR (400 MHz, $CDCl_3$, 298 K)

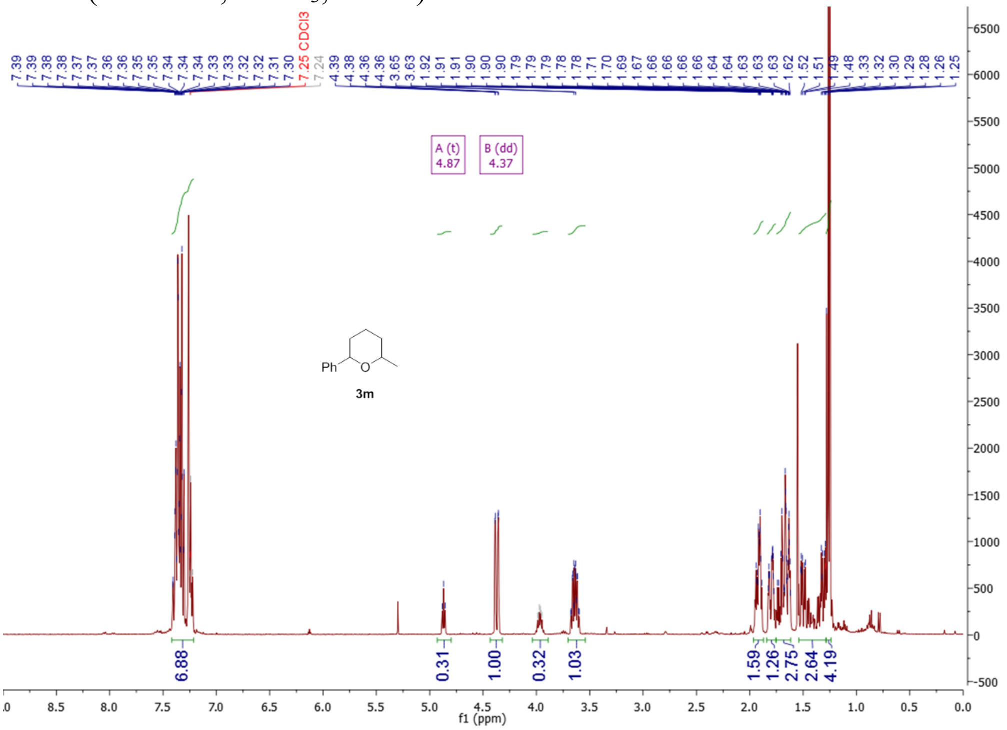

^{13}C{^{1}H} NMR (100 MHz, $CDCl_3$, 298 K)

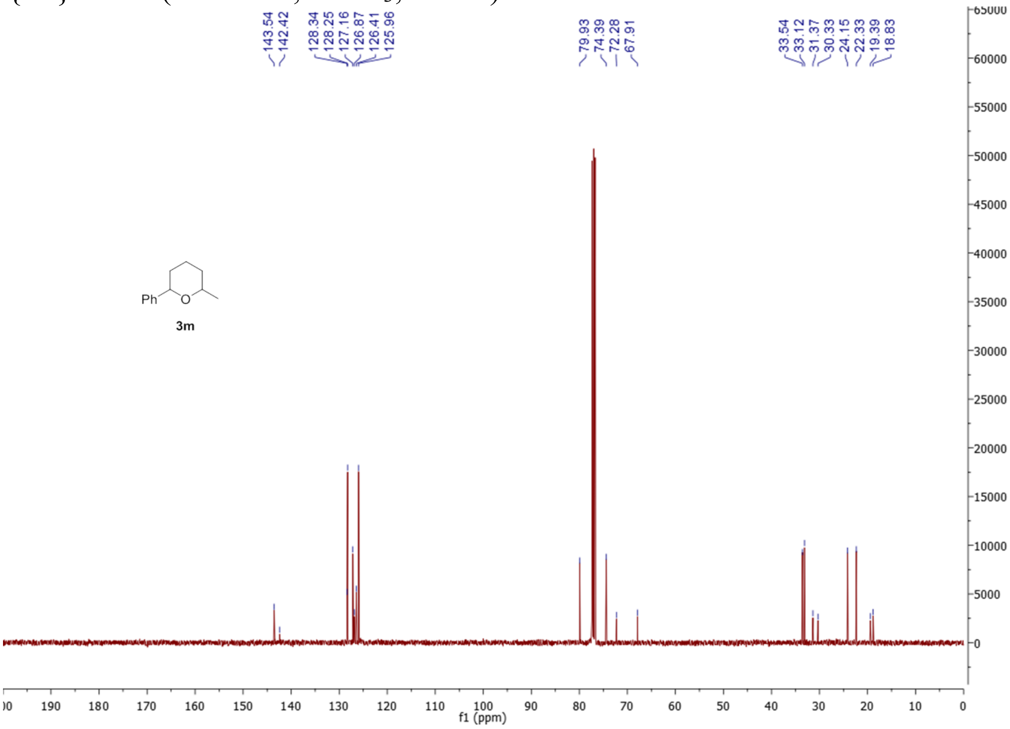

15.29. 2,6-Diphenyl-tetrahydropyran (3n), 2,6-diphenyl-2,3-dihydropyran (3n')

^{1}H NMR (400 MHz, $CDCl_3$, 298 K)

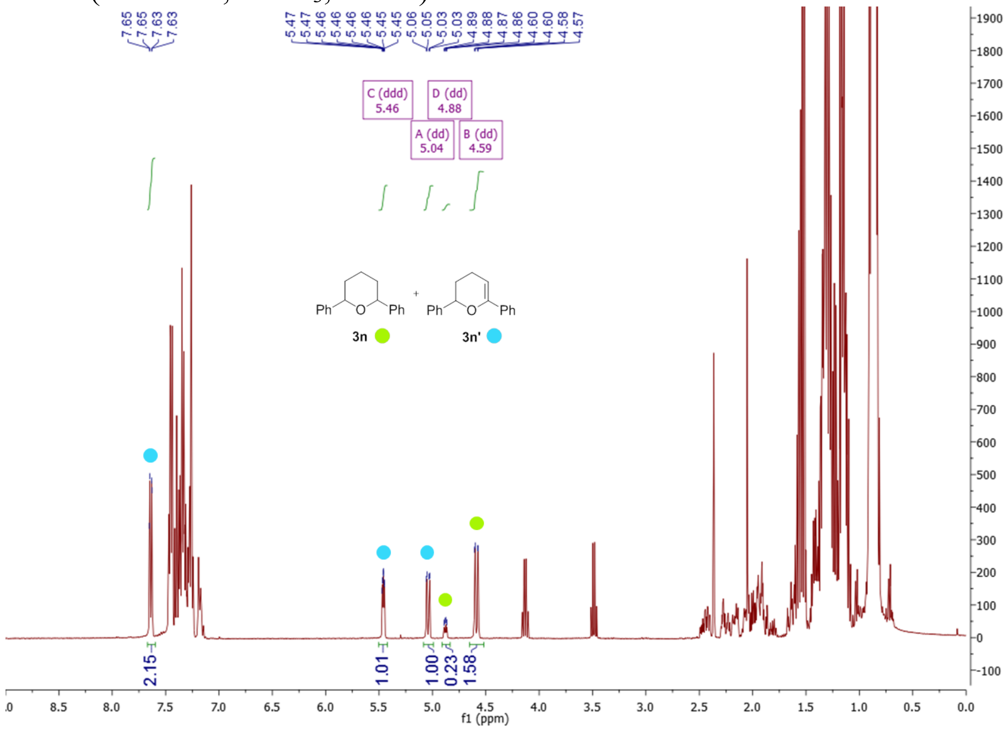

[1] Bartoszewicz, A.; Marcos, R.; Sahoo, S.; Inge, A. K.; Zou, X.; Martín-Matute, B. *Chem. Eur. J.* **2012**, *18*, 14510−14519.

[2] González Miera, G.; Martínez-Castro, E.; Martín-Matute, B. *Organometallics* **2018**, *37*, 636–644.

[3] Lan, X.-W.; Wang, N.-X.; Zhang, W.; Wen, J.-L.; Bai, C.-B.; Xing, Y.; Li, Y.-H. *Org. Lett.* **2015**, *17*, 4460–4463.

[4] Poce, G.; Cocozza, M.; Alfonso, S.; Consalvi, S.; Venditti, G.; Fernandez-Menendez, R.; Bates, R. H.; Barros Aguirre, D.; Ballell, L.; De Logu, A.; Vistoli, G.; Biava, M. *Eur. J. Med. Chem.* **2018**, *145*, 539–550.

[5] Deuri, S.; Phukan, P. *J. Phys. Org. Chem*. **2012**, *25*, 1228–1235.

[6] Martín-Matute, B.; Bäckvall, J.-E. *J. Org. Chem.* **2004**, *69*, 9191–9195.

[7] Call, A.; Casadevall, C.; Acuña-Parés, F.; Casitas, A.; Lloret-Fillol, J. *Chem. Sci.* **2017**, *8*, 4739–4749.

[8] Shibata, T.; Fujiwara, R.; Ueno, Y. *Synlett* **2005**, *1*, 0152–0154.

[9] Li, W.; Yang, C.; Gao, G.-L.; Xia, W. *Synlett.* **2016**, *27*, 1391−1396.

[10] Gharpure, S. J.; Vishwakarma, D. S.; Nanda, S. K. *Org. Lett.* **2017**, *19*, 6534−6537.

[11] Shi, H.; Liu, H.; Bloch, R.; Mandville, G. *Tetrahedron* **2001**, *57*, 9335−9341.

[12] Wysocki, J.; Ortega, N.; Glorius, F. *Angew. Chem. Int. Ed.* **2014**, *53*, 8751−8755.

[13] Reddy, A. R.; Zhou, C.-Y.; Guo, Z.; Wei, J.; Che, C.-M. *Angew. Chem. Int. Ed.* **2014**, *53*, 14175−14180.

[14] Dzudza, A.; Marks, T. J. *Chem. Eur. J.* **2010**, *11*, 3403−3422.

[15] Gharpure, S. J.; Vishwakarma, D. S.; Nanda, S. K. *Org. Lett.* **2017**, *19*, 6534−6537.

[16] Jiang, X.; London, E. W.; Morris, D. J.; Clarkson, G. J.; Wills, M. *Tetrahedron* **2010**, *66*, 9828−98344.

[17] Yang, B.; Lihammar, R.; Bäckvall, J.-E. *Chem. Eur. J.* **2014**, *20*, 13517−13521.

[18] Lan, X.-W.; Wang, N.-X.; Zhang, W.; Wen, J.-L.; Bai, C.-B.; Xing, Y.; Li, Y.-H. *Org. Lett*. **2015**, *17*, 4460−4463.

[19] Nugent, J.; Schwartz, B. D. *Org. Lett*. **2016**, *18*, 3834−3837.

[20] Grigorjeva, L.; Kinens, A.; Jirgensons, A. *J. Org. Chem.* **2015**, *80*, 920–927.

[21] Ortiz, R.; Koukouras, A.; Marqués-López, E.; Herrera, R. P. *Arabian J. Chem.* **2018**, DOI: 10.1016/j.arabjc.2018.01.022.
